# Late Na^+^ current and protracted electrical recovery are critical determinants of the aging myopathy

**DOI:** 10.1038/ncomms9803

**Published:** 2015-11-06

**Authors:** Sergio Signore, Andrea Sorrentino, Giulia Borghetti, Antonio Cannata, Marianna Meo, Yu Zhou, Ramaswamy Kannappan, Francesco Pasqualini, Heather O'Malley, Mark Sundman, Nikolaos Tsigkas, Eric Zhang, Christian Arranto, Chiara Mangiaracina, Kazuya Isobe, Brena F. Sena, Junghyun Kim, Polina Goichberg, Matthias Nahrendorf, Lori L. Isom, Annarosa Leri, Piero Anversa, Marcello Rota

**Affiliations:** 1 Departments of Anesthesia and Medicine and Division of Cardiovascular Medicine, Brigham and Women’s Hospital, Harvard Medical School, 20 Shattuck Street, Boston, Massachusetts 02115, USA; 2 Department of Pharmacology, University of Michigan, Ann Arbor, Michigan 48109, USA; 3 Center for Systems Biology, Massachusetts General Hospital, Harvard Medical School, Boston, Massachusetts 02114, USA

## Abstract

The aging myopathy manifests itself with diastolic dysfunction and preserved ejection fraction. We raised the possibility that, in a mouse model of physiological aging, defects in electromechanical properties of cardiomyocytes are important determinants of the diastolic characteristics of the myocardium, independently from changes in structural composition of the muscle and collagen framework. Here we show that an increase in the late Na^+^ current (*I*_NaL_) in aging cardiomyocytes prolongs the action potential (AP) and influences temporal kinetics of Ca^2+^ cycling and contractility. These alterations increase force development and passive tension. Inhibition of *I*_NaL_ shortens the AP and corrects dynamics of Ca^2+^ transient, cell contraction and relaxation. Similarly, repolarization and diastolic tension of the senescent myocardium are partly restored. Thus, *I*_NaL_ offers inotropic support, but negatively interferes with cellular and ventricular compliance, providing a new perspective of the biology of myocardial aging and the aetiology of the defective cardiac performance in the elderly.

## Abstract (web summary)

The aging myopathy is characterized by diastolic dysfunction of unknown aetiology. Rota *et al*. show that increased late Na^+^ current (*I*_NaL_) underlies diastolic dysfunction in the aged heart, and that inhibiting *I*_NaL_ improves diastolic indices and corrects the kinetics of cardiomyocyte contraction and relaxation in aged mice.

## Graphical abstract

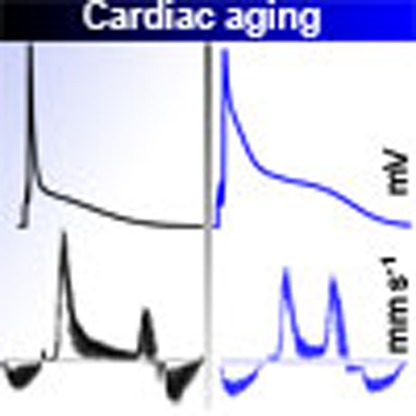

The aging myopathy typically manifests itself with diastolic dysfunction and preserved ejection fraction (EF)^1^. More than 50% of patients with heart failure have normal or near normal EF and the incidence and prevalence of this condition increases with age. Although the claim is commonly made that age-associated physiological changes predispose older adults to develop heart failure with normal EF, the aetiology of diastolic heart failure is unknown. The difficulty in defining myocardial aging and the mechanisms involved further complicates the recognition of the cellular processes underlying impaired diastolic relaxation. In the current study, we raised the possibility that, in a mouse model of physiological aging, defects in the electromechanical properties of cardiomyocytes are critical determinants of the altered cardiac performance, independently from changes in the structural composition of the myocardium and collagen framework. Cardiomyocytes constitute nearly 90% of the myocardium and alterations in the mechanisms of cell excitation, force generation and kinetics of contraction may be critical in conditioning the abnormalities of the old heart. Aging alters the pattern of electrical activation, resulting in prolonged myocardial repolarization^1^,^2^, which enhances the risk of ventricular arrhythmias and sudden death^3^. Moreover, prolongation of the time of repolarization and perturbations of intracellular ionic balance may influence the contractile and relaxation dynamics of the aging heart, impacting on diastolic and systolic function^4^.

Impaired diastolic function includes abnormalities in muscle relaxation, distensibility and ventricular filling pattern^5^. The increase in myocardial stiffness has been ascribed to changes in the composition of the extracellular matrix and/or to intrinsic characteristics of cardiomyocytes^6^. Switches in titin-isoforms and post-translational modifications of this cytoskeletal protein may affect the passive mechanical properties of myocytes, but whether defective electrical activation contributes to the process remains to be defined.

The mechanical activity of the myocardium is initiated by the electrical excitation of the tissue, which acts as a functional syncytium in propagating membrane depolarization in cardiomyocytes. The upstroke of the action potential (AP) triggers Ca^2+^ entry via L-type channels, which results in cytosolic Ca^2+^ rise by the translocation of Ca^2+^ from the sarcoplasmic reticulum (SR) to the cytoplasm, initiating cell contraction^7^. The repolarization phase of the AP is a relevant variable of the kinetics of Ca^2+^ entry; it controls the pattern of Ca^2+^ influx via L-type channels and Na^+^/Ca^2+^ exchanger (NCX) in a time- and voltage-dependent manner^7^,^8^,^9^,^10^,^11^,^12^. Thus, transmembrane Ca^2+^ fluxes and Ca^2+^-induced Ca^2+^ release modulate the temporal dynamics of [Ca^2+^]_i_ (refs 8, 9, 10, 11, 12), providing an important link between electrical activity, on the one hand, and myocyte and myocardial contractility and relaxation, on the other hand. This possibility has been tested here to document whether defects in this tightly controlled system become apparent in the senescent heart contributing to the manifestations of the aging myopathy and impaired diastolic function.

Here we show that aging leads to an increase in the late Na^+^ current (*I*_NaL_), which contributes to the prolongation of the AP duration (APD) and protracted electrical recovery of the aged myocardium. The remodelled electrical properties of cardiomyocytes provide inotropic support to the senescent muscle, but negatively interfere with cellular and myocardial relaxation. Inhibition of *I*_NaL_ partly corrects the defective electrical and diastolic properties of the aged heart. These findings offer a new perspective of the cellular and molecular mechanisms that define the aetiology of the aging myopathy and may have important biological and clinical implications.

## Results

### Aging impairs ventricular performance

**Figure 1:**
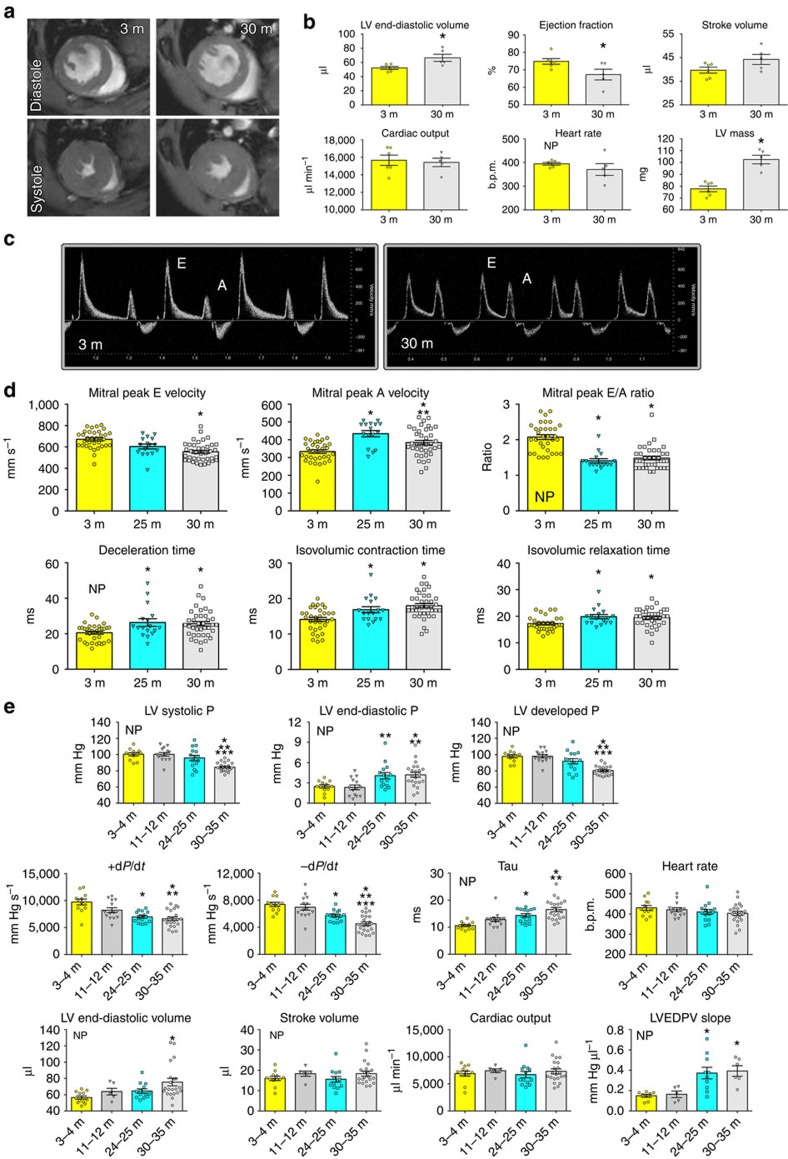
Aging is associated with a progressive deterioration of cardiac function. (**a**) Short axis views by MRI of young (3 months, 3 m) and old (30 months, 30 m) male mouse hearts. (**b**) Quantitative data obtained by MRI imaging in 3 months (3 m, *n*=6) and 30 months (30 m, *n*=5) male mice are shown as mean±s.e.m. and scatter plots. *P<0.05 versus 3 m (Student’s *t*-test and Mann–Whitney rank sum test); NP, non-parametric analysis. (**c**) Transmitral flow Doppler echocardiograms obtained in young (3 months, 3 m) and old (30 months, 30 m) male mice show early passive filling (*E*) and active filling (*A*) waves. (**d**) Quantitative data in male mice at 3 months (3 m, *n*=33), 25 months (25 m, *n*=19) and 30 months (30 m, *n*=38) are shown as mean±s.e.m. and scatter plots. *P<0.05 versus 3 m, **P<0.05 versus 25 m (one-way ANOVA with Bonferroni’s *post hoc* test and Kruskal–Wallis one-way ANOVA on ranks with Dunn’s *post hoc* test); NP, non-parametric analysis. (**e**) Haemodynamic and volumetric parameters obtained by pressure–volume (PV) catheterization in male mice. Data in animals at 3–4 months (3–4 m, *n*=12), 11–12 months (11–12 m, *n*=14), 24–25 months (24–25 m, *n*=15) and 30–35 months (30–35 m, *n*=19) are shown as mean±s.e.m. and scatter plots. LVEDPV, LV end-diastolic PV relation; P, pressure. *P<0.05 versus 3–4 m, **P<0.05 versus 11–12 m, ***P<0.05 versus 24–25 m (one-way ANOVA with Bonferroni’s *post hoc* test and Kruskal–Wallis one-way ANOVA on ranks with Dunn’s *post hoc* test); m, months; NP, non-parametric analysis.

To determine the effects of aging on left ventricular (LV) function, echocardiographic measurements, including speckle-tracking and Doppler protocols, were combined with magnetic resonance imaging (MRI) and invasive haemodynamics. Mice from 3 to 35 months of age were studied. Systolic blood pressure, evaluated by the tail-cuff plethysmography, was comparable in unanaesthetized mice at 3, 25 and 30 months of age, indicating that systemic blood pressure did not increase with aging in this model (Supplementary Fig. 1). EF was preserved at 24 months, when an increase in LV mass was observed. EF slightly decreased at 30 months together with an increase in cavitary volume, whereas stroke volume and cardiac output were maintained (Supplementary Fig. 2). These properties were confirmed by cardiac MRI^13^ (Fig. 1a,b). In addition, speckle-tracking strain echocardiographic imaging demonstrated that radial and longitudinal strain and strain rates were altered in old mice (Supplementary Fig. 3). Importantly, following blockade of the autonomous nervous system^14^, differences in EF observed between mice at 3–4 and 30–31 months of age were attenuated (Supplementary Fig. 4), indicating that alterations in cardiac performance with age are partly dictated by an imbalance in autonomic nerve activity, as reported previously^15^,^16^.

To define the diastolic properties of the LV, transmitral flow Doppler echocardiography was employed^17^. Mice at 25 months of age and older presented reduced passive filling velocity (*E*-wave), increased active filling (*A*-wave), lower *E*/*A* ratio and prolonged timing parameters (Fig. 1c,d). Haemodynamically, an increase in end-diastolic pressure occurred at 24–25 months and preceded the deterioration in systolic performance; in fact, LV systolic pressure, developed pressure and +*d*P/*d*t decreased significantly in mice at 30–35 months of age, although stroke volume and cardiac output were preserved (Fig. 1e). In contrast, reduced −*d*P/*d*t, prolonged time constant of pressure decay (*τ*), and increased end-diastolic pressure were present at 24–25 months, documenting a decline in diastolic function in the absence of systolic defects. In addition, occlusion of the inferior vena cava or aortic arch, which alters LV loading, showed that the slope of the LV end-diastolic pressure–volume (PV) relation was 2.5-fold steeper in old than in young animals, pointing to alterations in ventricular compliance with age (Fig. 1e). The deterioration of diastolic and systolic haemodynamic indices in mice ≥30 months was also observed following complete autonomic blockade (Supplementary Fig. 5). Thus, these *in vivo* data emphasize the defective kinetics of contraction and relaxation, and the impaired diastolic filling of the old heart, which precede, chronologically, the compromise of systolic function.

LV weight was similar in mice at 3 and 11 months of age, and increased modestly at 23–25 months. Myocardial hypertrophy became apparent in the senescent heart at 28–34 months (Supplementary Fig. 6a), when cardiac performance was depressed. Structurally, cardiomyocytes were 34% larger in senescent mice; cellular hypertrophy was associated with changes in the expression of fibrotic markers^18^ and a relatively modest increase in myocardial interstitial fibrosis, from 0.2% at 2–3 months of age to 0.3% at 29–30 months (Supplementary Fig. 6b–e). Collectively, these results support the notion that the aging myopathy is characterized by an initial decline of diastolic function, dictated by protracted kinetics of contraction and relaxation. These alterations are later accompanied by intervening defects in systolic performance, and myocyte and myocardial hypertrophy.

### Aging delays myocardial and myocyte electrical recovery

**Figure 2:**
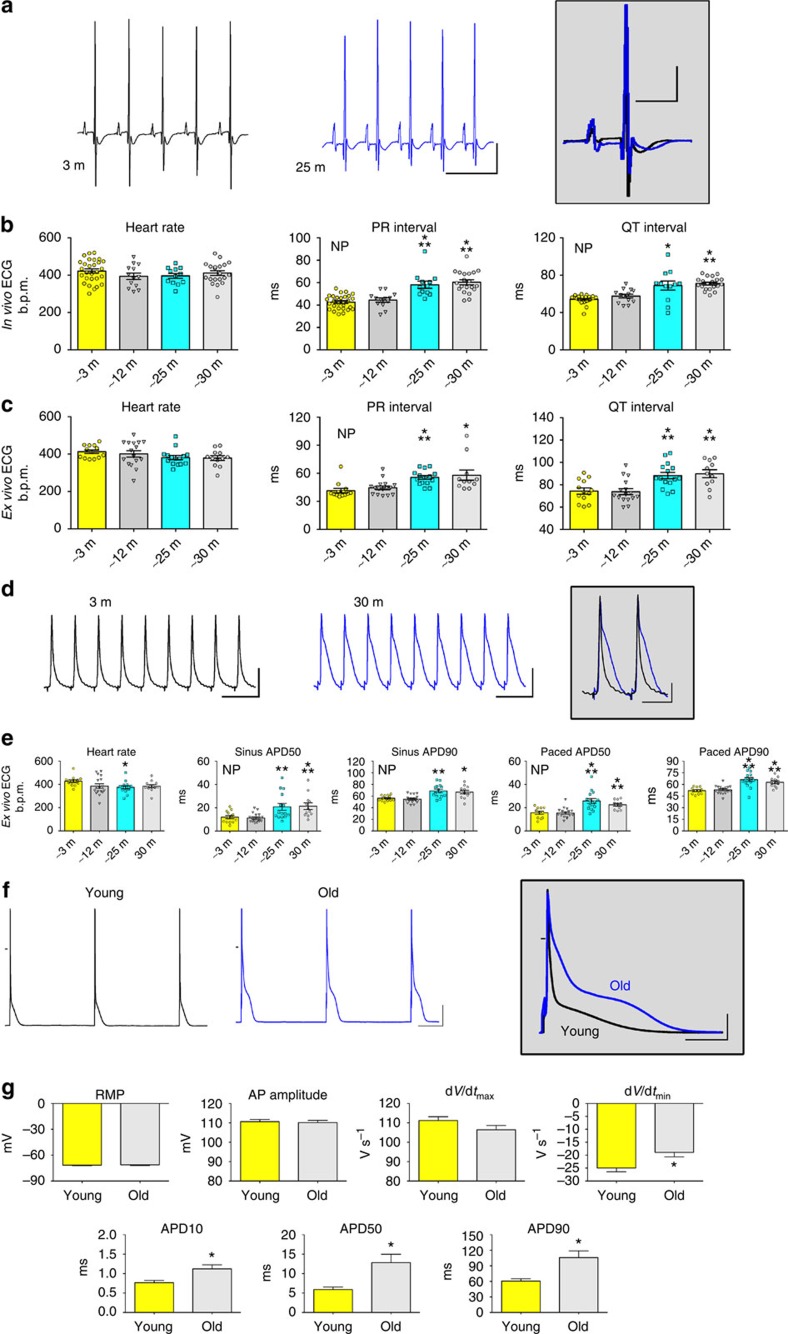
Aging leads to electrical abnormalities. (**a**) ECGs from a young (3 months, 3 m; black traces) and old (25 months, 25 m; blue traces) mouse. Scale bars, 200 ms, 0.2 mV. Superimposed traces are shown in the inset. Scale bars, 50 ms, 0.2 mV (**b**,**c**) Electrocardiographic parameters in anaesthetized male mice (**b**) and explanted hearts (**c**) at 3–4 (∼3 m, *n*=28,13), 11–13 (∼12 m, *n*=13,16), 24–27 (∼25 m, *n*=12,15) and 30–34 months (∼30 m, *n*=20,12) are mean±s.e.m. and scatter plots. *P<0.05 versus ∼3 m, **P<0.05 versus ∼12 m (one-way ANOVA with Bonferroni’s *post hoc* test and Kruskal–Wallis one-way ANOVA on ranks with Dunn’s *post hoc* test); NP, non-parametric analysis. (**d**) Epicardial monophasic action potentials (MAPs) in perfused hearts from male mice at 3 (3 m; black traces) and 30 months (30 m; blue traces). Scale bars, 200 ms, 4 mV. Superimposed traces are shown in the inset. Scale bars, 100 ms, 2 mV (**e**) Data at sinus rhythm (Sinus) or 8 Hz stimulation (Paced) in hearts from male mice at 3–4 (∼3 m, *n*=11), 11–13 (∼12 m, *n*=16), 24–27 (∼25 m, *n*=15) and 30 months (30 m, *n*=11) are mean±s.e.m. and scatter plots. APD50, APD90: MAP duration at 50 and 90% repolarization, respectively. *P<0.05 versus ∼3 m, **P<0.05 versus ∼12 m (one-way ANOVA with Bonferroni’s *post hoc* test and Kruskal–Wallis one-way ANOVA on ranks with Dunn’s *post hoc* test); NP, non-parametric analysis. (**f**) APs recorded in LV myocytes from young (black traces) and old (blue traces) mice. Scale bars, 300 ms, 20 mV. Superimposed traces are shown in the inset. Scale bars, 50 ms, 20 mV. (**g**) AP parameters of myocytes from male mice at 3 (Young, *n*=50 cells from 18 hearts) and 26–30 months (Old, *n*=42 cells from 23 hearts) are mean±s.e.m. RMP, resting membrane potential. *P<0.05 versus Young (Mann–Whitney rank sum test). Scattered plots are reported in Supplementary Fig. 10. m, months.

Surface electrocardiogram (ECG) was employed to assess the electrical properties of the heart in mice at 3, 11–13, 25 and 30–34 months of age. PR and QT intervals were prolonged in mice ≥25 months (Fig. 2a,b), indicating that delays in atrioventricular conduction and ventricular repolarization occurred with age. These changes were also observed by telemetry in conscious animals and persisted following autonomic blockade (Supplementary Figs 7 and 8). To exclude the confounding effects of circulating neurohumoral factors, an isolated organ preparation was employed^12^. Again, old hearts showed delayed PR and QT intervals (Fig. 2c). In addition, epicardial monophasic APs (MAPs), which reflect local cellular depolarization and repolarization, were recorded on the LV free wall^12^. APD was prolonged in old hearts (Fig. 2d,e), suggesting that defects in the electrical properties of the aging myocardium were mediated by alterations at the cellular level. Since repolarization delays are associated with electrical disturbances^3^,^12^, the propensity of the aging heart to develop arrhythmias was assessed by a protocol of programmed electrical stimulation (PES)^12^. Arrhythmic events were 2.6-fold more frequent in hearts ≥24 months of age (Supplementary Fig. 9).

The electrical properties of isolated LV myocytes were established by the patch-clamp technique^11^,^12^. The duration of the AP, measured at 90% (APD_90_) of repolarization, was 1.8-fold longer in 26–30-month-old myocytes (Fig. 2f,g and Supplementary Fig. 10). The changes in APD detected in old myocytes were consistent with the 24% reduction in the maximal negative slope during repolarization (d*V*/d*t*_min_). However, resting membrane potential, AP amplitude and d*V*/d*t*_max_ were comparable in myocytes at all ages. These electrical parameters, obtained at 1 Hz pacing rate, were maintained at higher stimulation frequency (Supplementary Fig. 11). Collectively, myocardial aging is characterized by electrical disturbances and the prolongation of the AP in myocytes contributes to the delayed electrical recovery of the old heart.

### Aging alters transmembrane ionic currents

**Figure 3:**
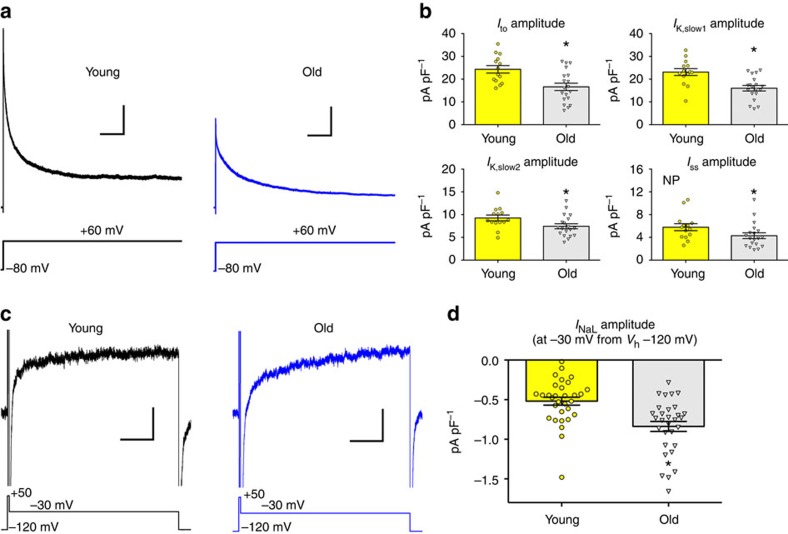
Aging alters the ionic current profile of LV cardiomyocytes. (**a**) Whole-cell voltage-gated K^+^ currents recorded in voltage-clamp in a young (black traces) and an old (blue traces) LV myocyte. Scale bars, 3 s, 10 pA pF^−1^. Voltage-clamp protocol is reported in the lower traces. (**b**) Quantitative data for the amplitude of Kv current components in myocytes from mice at 3–4 months (Young, *n*=14 cells from 5 hearts) and 31–32 months (Old, *n*=19 cells from 4 hearts) are shown as mean±s.e.m. *P<0.05 versus Young (Student’s *t*-test and Mann–Whitney rank sum test); NP, non-parametric analysis. (**c**) Whole-cell voltage-gated Na^+^ currents recorded in voltage-clamp in a young (black traces) and an old (blue traces) LV myocyte. Scale bars, 100 ms, 0.6 pA pF^−1^. The voltage-command protocol, which is shown in the lower traces, comprised a preconditioning pulse to +50 mV to prevent Na^+^ influx and minimize loss of voltage control. Alternative voltage-clamp protocols were also employed (see Supplementary Figs 13 and 14). (**d**) Quantitative data in myocytes from male mice at 3 months (Young, *n*=31 cells from 4 hearts) and 30 months (Old, *n*=30 cells from 4 hearts) are shown as mean±s.e.m. and scatter plots. *P<0.001 versus Young (Mann–Whitney rank sum test).

Voltage-gated outward K^+^ Kv currents are primary determinants of the AP repolarization and major factors in the electrophysiological remodelling occurring in myocytes during maturation, cardiac pathology and aging^8^,^19^,^20^,^21^,^22^,^23^. Kv currents comprise components with rapid activation and fast (*I*_to_), intermediate (*I*_K,slow1_) and slow (*I*_K,slow2_) kinetics of inactivation, together with a sustained (non-inactivating) component (*I*_ss_)^21^,^24^. By voltage-clamp protocols^24^, Kv currents were reduced in LV myocytes from old mice, at 31–32 months of age (Fig. 3a,b and Supplementary Fig. 12). Thus, reduction of Kv currents in old myocytes contributes partly to the prolonged repolarization of the AP.

Next, we tested whether changes in the slowly inactivating late Na^+^ (*I*_NaL_) and L-type Ca^2+^ (*I*_CaL_) inward currents were implicated in the protracted AP. *I*_NaL_ current density was 1.6-fold higher in 30-month-old cardiomyocytes than in 3-month-old cells (Fig. 3c,d). In addition, *I*_NaL_ maximal conductance was increased, whereas voltage activation and steady-state inactivation were comparable in young and old cells (Supplementary Fig. 13). Moreover, we dissected pharmacologically *I*_NaL_ in young and old myocytes using tetrodotoxin (TTX) and found that the TTX-sensitive slowly inactivating current was increased in old cardiomyocytes (Supplementary Fig. 14). The fast inactivating Na^+^ current (*I*_Na_) that controls the upstroke of the AP was not altered with aging (Supplementary Fig. 15), a result which is consistent with the preservation of the amplitude and upstroke velocity of the AP. Similarly, the density and properties of *I*_CaL_ were comparable in young and old cells (Supplementary Fig. 16).

**Figure 4:**
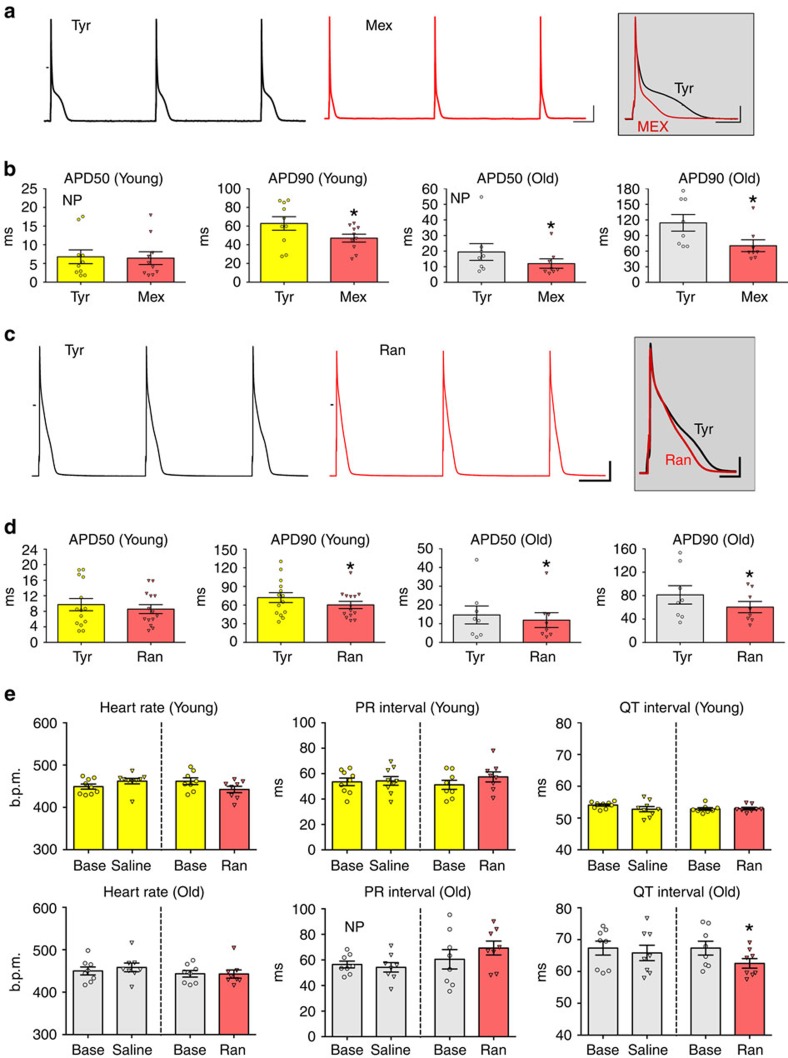
Inhibition of *I*_NaL_ shortens the repolarization properties of the old heart. (**a**) APs recorded in an old myocyte before (Tyrode, black traces) and after exposure to the Na^+^ channel inhibitor mexiletine (Mex, red traces). Scale bars, 200 ms, 20 mV. Superimposed traces are reported in the inset. Scale bars, 500 ms, 20 mV. (**b**) Quantitative data in myocytes from male mice at 3 months (Young, *n*=10 cells from 5 hearts) and 27–30 months (Old, *n*=8 cells from 5 hearts) before (Tyr) and after exposure to 10 μM mexiletine (Mex) are shown as mean±s.e.m. and scatter plots. Tyr, Tyrode solution. *P<0.05 versus Tyr (paired *t*-test and Wilcoxon signed rank test); NP, non-parametric analysis. Additional parameters are reported in Supplementary Fig. 18. (**c**) APs recorded in an old myocyte before (Tyrode, black traces) and after exposure to *I*_NaL_ inhibitor ranolazine (Ran, red traces). Scale bars, 300 ms, 20 mV. Superimposed traces are reported in the inset. Scale bars, 50 ms, 20 mV. (**d**) Quantitative data in myocytes from mice at 3–6 months (Young, *n*=14 cells from 4 hearts) and 27–33 months (Old, *n*=8 cells from 5 hearts) before (Tyr) and after exposure to 10 μM ranolazine (Ran) are shown as mean±s.e.m. and scatter plots. *P<0.05 versus Tyr (paired *t*-test). Additional parameters are reported in Supplementary Fig. 18. (**e**) Quantitative data for electrocardiographic parameters obtained in male mice at 3–4 months (Young) and at 30–31 months (Old) at baseline (Base) and 1 h after treatment with saline (Young, *n*=9; Old *n*=8) or ranolazine (30 mg per kg body weight, i.p.) (Young, *n*=8; Old *n*=8). Data are shown as mean±s.e.m. and scatter plots. *P<0.05 versus Base (paired *t*-test and Wilcoxon signed rank test); NP, non-parametric analysis.

The role of *I*_NaL_ in myocyte repolarization was determined by measuring the AP before and after inhibition of this current. We employed ranolazine, a selective inhibitor of *I*_NaL_ (refs 25, 26, 27), and mexiletine, a blocker of the late Na^+^ current that, as previously reported^28^,^29^, minimally affects peak *I*_Na_ (Supplementary Fig. 17). Reduction of *I*_NaL_ shortened the intermediate and late repolarization phases of the AP in old myocytes, but had an attenuated impact on young cells (Fig. 4a–d and Supplementary Fig. 18). Moreover, the duration of LV MAP measured at 90% repolarization was reduced following mexiletine perfusion by 14 and 22% in 5- and 24–30-month-old hearts, respectively (Supplementary Fig. 19), indicating that inhibition of *I*_NaL_ abbreviates the repolarization phase of the AP.

To establish whether *I*_NaL_ was implicated in the prolongation of the QT interval in the senescent heart (see Fig. 2b), mice were treated systemically with an inhibitor of *I*_NaL_, and ECGs were recorded at baseline and 1 h after administration of the inocula. In young animals, inhibition of *I*_NaL_ had no major consequences on the electrical recovery of the myocardium. Conversely, blockade of *I*_NaL_ in old mice with ranolazine or mexiletine shortened the QT interval by 7% and 8%, respectively (Fig. 4e and Supplementary Fig. 20a). These results were confirmed in conscious mice by telemetry (Supplementary Fig. 20b). Thus, alterations in *I*_NaL_ prolong myocyte AP and electrical recovery of the senescent myocardium, and inhibition of *I*_NaL_ partially corrects this defect, restoring *in vivo* a younger cardiac phenotype.

### Electrical activity modulates Ca^2+^ transients in myocytes

**Figure 5:**
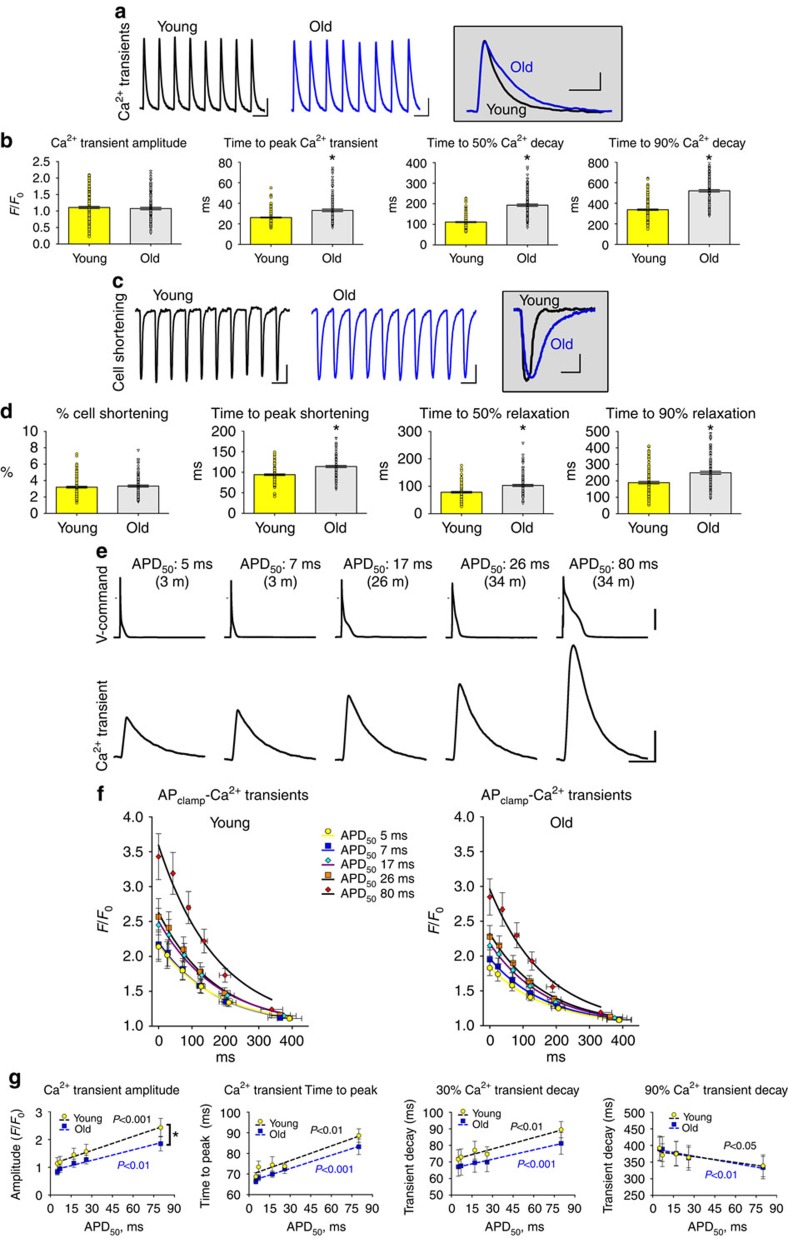
Electromechanical properties of aging LV myocytes. (**a**) Ca^2+^ transients of young (black traces) and old (blue traces) myocytes. Scale bars, 1 s, 0.25 *F*/*F*_0_. Superimposed traces are reported in the inset. Scale bars, 250 ms, 0.25 *F*/*F*_0_. (**b**) Quantitative data for Ca^2+^ transients obtained in myocytes from mice at 3 months (Young) and 29–30 months (Old) (Young, *n*=195 cells from 16 hearts; Old, *n*=131 cells from 11 hearts); data are shown as mean±s.e.m. and scatter plots. *P<0.05 versus Young (Mann–Whitney rank sum test). (**c**) Cell shortening of young (black traces) and old (blue traces) myocytes. Scale bars, 1 s, 1 μm. Superimposed traces are reported in the inset. Scale bars, 250 ms, 1 μm (**d**) Quantitative data for cell shortening properties obtained in myocytes from mice at 3 months (Young) and 29–30 months (Old) (Young, *n*=129 cells from 8 hearts; Old, *n*=117 cells from 7 hearts); data are shown as mean±s.e.m. and scatter plots. *P<0.05 versus Young (Mann–Whitney rank sum test). (**e**) AP profiles (upper traces) obtained from myocytes at different age (m, months) were applied as voltage-clamp command (AP-clamp) in an old myocyte to elicit Ca^2+^ transients (lower traces). Scale bars, 300 ms, 1 *F*/*F*_0_, 40 mV. Ionic currents recorded are omitted. (**f**) Decay of Ca^2+^ transients elicited by five different AP-clamp protocols obtained in myocytes from male mice at 3–5 months (Young) and 28–33 months (Old) are shown as mean±s.e.m. (Young, *n*=12 cells from 5 hearts; Old, *n*=9 cells from 2 hearts). Levels of the Ca^2+^ fluorescence signal from the peak of the Ca^2+^ transient to 90% of the decay are reported. Data were fitted with mono-exponential functions (solid lines). Fitting parameters are reported in Supplementary Table 3. (**g**) Bivariate plot for APD50 of the AP used as *V*_clamp_ command and Ca^2+^ transient properties obtained from young and old myocytes reported in **f**. Data were fitted with linear regressions (dashed lines). Fitting parameters are reported in Supplementary Table 3.

The repolarization of the AP controls the transmembrane Ca^2+^ influx via L-type channels and the NCX, which, in turn, activates the release of Ca^2+^ from the SR^7^,^8^,^9^,^10^,^11^,^12^. Thus, we tested whether the protracted electrical repolarization of the AP in senescent myocytes was associated with alterations in Ca^2+^ cycling and cell contractility. Ca^2+^ transients and unloaded cell shortening were evaluated in young, 3 months, and old, 29–30 months, myocytes. At 1 Hz, Ca^2+^ transient amplitude and cell shortening were similar in both groups of myocytes, but time to peak Ca^2+^ transient was delayed by 26%, and time to peak shortening was prolonged by 21% in old cells. Moreover, Ca^2+^ decay and myocyte relengthening at 90% were, respectively, 54% and 32% longer in old myocytes. The delay in the timing parameters was maintained at higher stimulation frequencies, although a reduction in developed Ca^2+^ was observed (Fig. 5a–d, Supplementary Fig. 21).

To establish a causative link between the prolongation of the AP in old cardiomyocytes and the delayed kinetics and preserved amplitude of Ca^2+^ transients, AP-clamp experiments were performed. Two short APs (APD_50_: 5 and 7 ms) collected from two young myocytes and three longer APs (APD_50_: 17, 26 and 80 ms) obtained from three old cells were employed as voltage-clamp command while monitoring intracellular Ca^2+^ levels. As previously reported^11^,^12^,^30^, longer APD led to a progressive increase in Ca^2+^ transient amplitude, but the slope of the positive APD-inotropic relationship was attenuated in old myocytes (Fig. 5e–g). In young and old cardiomyocytes, increases in APD resulted in delayed time to peak Ca^2+^ and Ca^2+^ decay at 30%; however, the late decay, measured at 90%, negatively correlated with APD. When the same AP shape was imposed to young and old myocytes, the timing parameters of Ca^2+^ transients were comparable in young and old cells, although Ca^2+^ transient amplitude tended to be reduced in senescent myocytes (Fig. 5f and Supplementary Fig. 22). These results suggest that (i) prolongation of the AP increases the amplitude of Ca^2+^ transients in cardiomyocytes, but the efficiency of the process is attenuated in old cells; (ii) longer durations of the AP delay time to peak and early decay of Ca^2+^ transients; and (iii) experimental conditions, including cell dialysis, abolish the slower Ca^2+^ transient kinetics observed in old cells with field stimulation.

**Figure 6:**
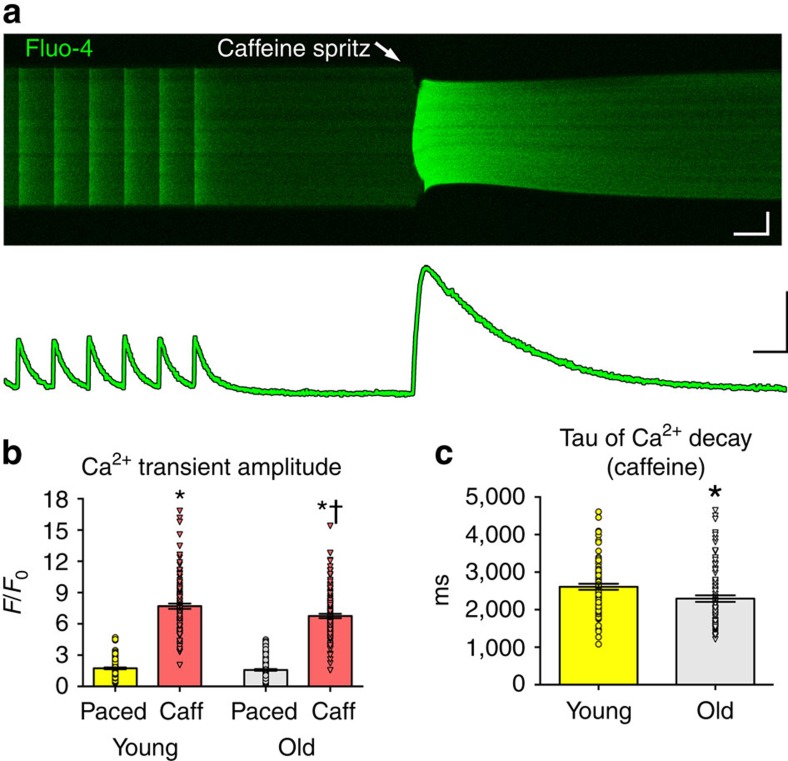
SR Ca^2+^ load of aging LV myocytes. (**a**) Line scan image of a young myocyte loaded with the Ca^2+^ indicator Fluo-4 (green). Ca^2+^ transients were elicited by electrical pacing or by 20 mM caffeine spritz (arrow). Scale bars, 1 s, 20 μm. Traces are reported in the lower panel. Scale bars, 1 s, 30 a.u. (**b**) Quantitative data for myocytes from male mice at 3–6 months (Young, *n*=120 cells from 4 hearts) and 27 months (Old, *n*=131 cells from 5 hearts) are shown as mean±s.e.m. and scatter plots. Caff, caffeine. *P<0.001 versus Paced (Wilcoxon Signed Rank Test); †*P*<0.01 versus Young (Mann–Whitney rank sum Test). (**c**) Quantitative data for the caffeine-induced Ca^2+^ transient decay obtained from a subset of cells represented in **b** (Young, *n*=90 cells from 4 hearts; Old, *n*=86 cells from 5 hearts) are shown as mean±s.e.m. and scatter plots. *P<0.05 versus Tyr (Mann–Whitney rank sum test).

The lack of potentiated Ca^2+^ transient amplitude in old myocytes with protracted APD raised the possibility that SR Ca^2+^ load was affected with age, a factor that may limit Ca^2+^ release following electrical excitation^31^. Field-stimulated myocytes were rapidly exposed to caffeine to assess SR Ca^2+^ content^11^,^12^,^32^. The amplitude of the AP-induced Ca^2+^ transient was comparable in young and old cells but the caffeine-induced Ca^2+^ release was reduced in old myocytes (Fig. 6a,b), suggesting a defective SR Ca^2+^ load. The decay of the caffeine-induced Ca^2+^ transient that reflects NCX activity^33^ was faster in old myocytes (Fig. 6c). Thus, a reduced SR Ca^2+^ load in old myocytes may condition the attenuated amplitude of the Ca^2+^ transients in spite of a prolongation of the AP.

### *I*_NaL_ and modulation of myocyte mechanics and Ca^2+^ transients

**Figure 7:**
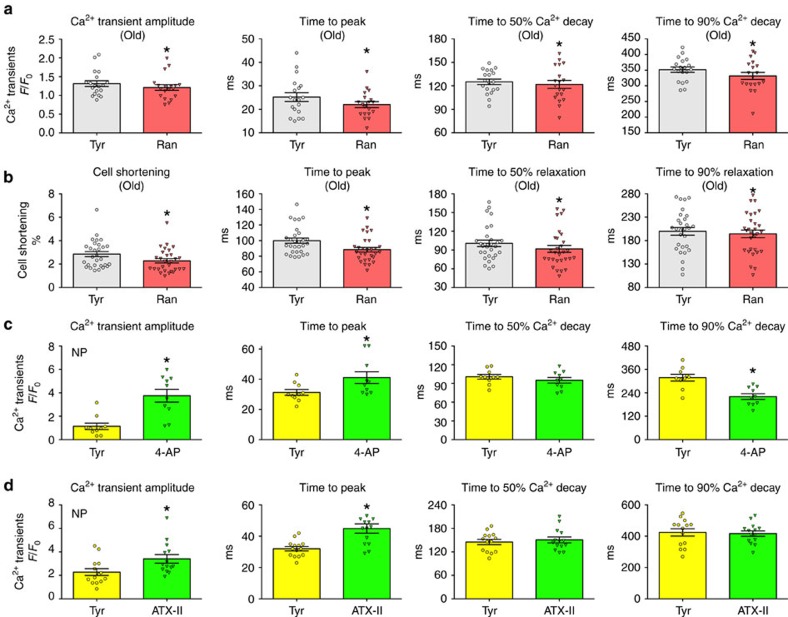
*I*_NaL_ influences the mechanical properties of LV myocytes. (**a**) Quantitative data for Ca^2+^ transients in myocytes from male mice at 30–33 months, before (Tyr) and after exposure to 10 μM ranolazine (Ran) (*n*=19 cells from 5 hearts); data are shown as mean±s.e.m. and scatter plots. *P<0.05 versus Tyr (paired *t*-test). (**b**) Quantitative data for unloaded cell shortening properties in myocytes from male mice at 30–33 months, before (Tyr) and after exposure to 10 μM ranolazine (Ran) (*n*=29 cells from 4 hearts); data are shown as mean±s.e.m. and scatter plots. *P<0.05 versus Tyr (paired *t*-test). (**c**) Quantitative data for Ca^2+^ transients in myocytes from male mice at 3–6 months, before (Tyr) and after exposure to 0.5 mM 4-aminopyridine (4-AP) (*n*=10 cells from 2 hearts); data are shown as mean±s.e.m. and scatter plots. *P<0.01 versus Tyr (paired *t*-test and Wilcoxon Signed Rank Test); NP, non-parametric analysis. (**d**) Quantitative data for Ca^2+^ transients in myocytes from mice at 3–9 months, before (Tyr) and after exposure to 1 nM anemonia toxin-II (ATX-II) (*n*=14 cells from 4 hearts); data are shown as mean±s.e.m. and scatter plots. *P<0.001 versus Tyr (paired *t*-test and Wilcoxon Signed Rank Test); NP, non-parametric analysis.

The prolonged APD explains only in part the defective properties of Ca^2+^ transients in old myocytes. Additional factors potentially contributing to the alterations in Ca^2+^ transient decay were considered. Enhanced Na^+^ influx via *I*_NaL_ may increase intracellular Na^+^ load in old cardiomyocytes^34^,^35^,^36^,^37^, a condition inhibiting diastolic Ca^2+^ extrusion via NCX and affecting Ca^2+^ transient decay^26^,^37^,^38^. To test this possibility, *I*_NaL_ was inhibited in field-stimulated myocytes while monitoring Ca^2+^ transients and cell shortening. Consistent with the shortening of the duration of the AP, this intervention decreased the amplitude and time to peak Ca^2+^ transients and cell twitching. However, the kinetics of Ca^2+^ decay and myocyte relengthening were reduced in old cells, in contrast with the changes observed with AP-clamp tests (Fig. 7a,b and Supplementary Fig. 23). Therefore, 4-aminopyridine (4-AP) or anemonia toxin-II (ATX-II) were employed to prolong the duration of the AP (Supplementary Fig. 24) by selectively inhibiting Kv currents^11^,^22^,^24^ or by enhancing *I*_NaL_ (refs 29, 39), respectively. In both cases, the prolongation of the AP increased the amplitude of Ca^2+^ transients and delayed time to peak of Ca^2+^. However, 4-AP led to a faster Ca^2+^ transient decay, whereas the enhanced *I*_NaL_ with ATX-II failed to shorten the kinetics of Ca^2+^ decay (Fig. 7c,d). Collectively, these findings suggest that enhanced Na^+^ influx opposes the faster Ca^2+^ decay associated with increases in APD.

### *I*_NaL_, myocardial and cardiac function

**Figure 8:**
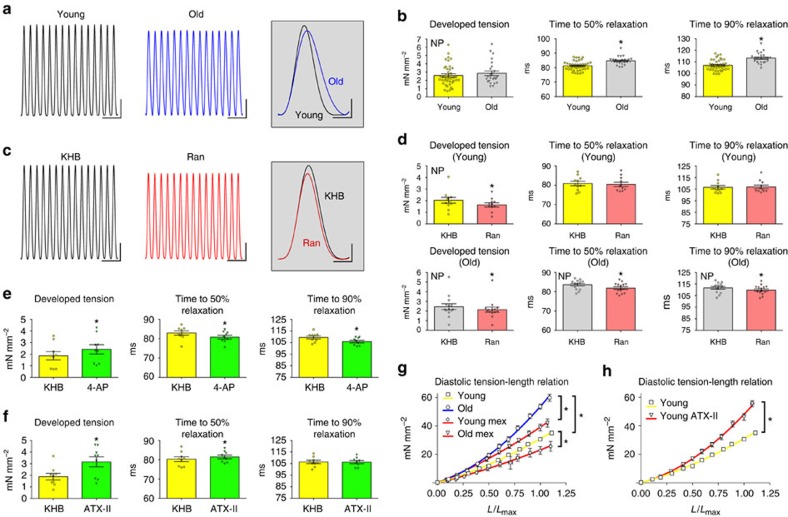
*I*_NaL_ alters myocardial function. (**a**) Isometric contraction for young and old muscles. Scale bars, 500 ms, 0.5 mN mm^−2^. Traces are superimposed in the inset. Scale bars, 50 ms, 0.5 mN mm^−2^ (**b**) Properties of papillary muscles from male mice at 3–6 (Young, *n*=41) and 30–33 months (Old, *n*=24) are shown as mean±s.e.m. and scatter plots. *P<0.001 versus Young (Student’s *t*-test); NP, non-parametric analysis. (**c**) Contraction for an old muscle prior (black traces) and following ranolazine exposure (Ran, red traces). Scale bars, 500 ms, 0.6 mN mm^−2^. Traces are superimposed in the inset. Scale bars, 50 ms, 0.6 mN mm^−2^. (**d**) Data for muscles of male mice at 3–5 (Young, *n*=11) and 30–33 months (Old, *n*=14) before and after exposure to 10 μM ranolazine are mean±s.e.m. and scatter plots. *P<0.001 versus KHB (Wilcoxon signed rank test); NP, non-parametric analysis. (**e**) Data for muscles of male mice at 3–6 months (*n*=9) before and after exposure to 1 mM 4-AP are mean±s.e.m. and scatter plots. *P≤0.001 versus KHB (paired *t*-test). (**f**) Data for muscles of mice at 3–9 months (*n*=9) before and after exposure to 10 nM ATX-II are mean±s.e.m. and scatter plots. *P<0.05 versus KHB (paired *t*-test). (**g**) Diastolic length–tension relations for twitching muscles stimulated at 6 Hz in the absence or presence of mexiletine (Mex). Muscles were obtained from male mice at 3 (Young, *n*=22; Young-Mex, *n*=6) and 30–33 months (Old, *n*=8; Old-Mex, *n*=8); data are mean±s.e.m. Fitting: second-order polynomial function (see Supplementary Table 3). *P<0.05 between selected fittings (one-way ANOVA with Bonferroni’s *post hoc* test). (**h**) Diastolic length–tension relations of muscles from male mice at 3 months in the absence (Young, data as in **g** and presence of the *I*_NaL_ enhancer ATX-II (*n*=10); data are shown as mean±s.e.m. and fitted with a second order polynomial function. *P<0.0001 between fittings (Student’s *t*-test). Parameters are reported in Supplementary Table 3.

To determine whether alterations in *I*_NaL_ and its effects on senescent cardiomyocytes were equally operative at the level of the myocardium, LV papillary muscles from young, 3–6 months, and old, 30–33 months, hearts were studied in an isometric system. Developed tension was similar in both muscles, but twitch kinetics, measured as the time of relaxation from the beginning of contraction, was prolonged with aging (Fig. 8a,b). Inhibition of *I*_NaL_ with ranolazine or mexiletine reduced developed force and accelerated relaxation times, which were more apparent in old muscles (Fig. 8c,d and Supplementary Fig. 25). In addition, to define the adaptation of papillary muscles to the protracted electrical recovery and enhanced Na^+^ entry, 4-AP and ATX-II were employed. 4-AP had a positive inotropic effect, which was coupled with faster twitches (Fig. 8e); however, the enhanced *I*_NaL_ with ATX-II increased developed force but failed to accelerate myocardial contractile dynamics (Fig. 8f).

Subsequently, the influence of Na^+^ influx and altered twitch kinetics on diastolic tension of the myocardium was evaluated. The passive length–tension relationship was measured by progressively stretching papillary muscles stimulated at 6 Hz, from near slack length (*L*_0_) to the length at which maximal force (*L*_max_) was developed. Diastolic tension was higher in the senescent myocardium and inhibition of Na^+^ influx shifted the relation to lower passive tension (Fig. 8g). In contrast, enhancement of Na^+^ entry with ATX-II in young muscles shifted the diastolic length–tension curve to higher values (Fig. 8h).

**Figure 9:**
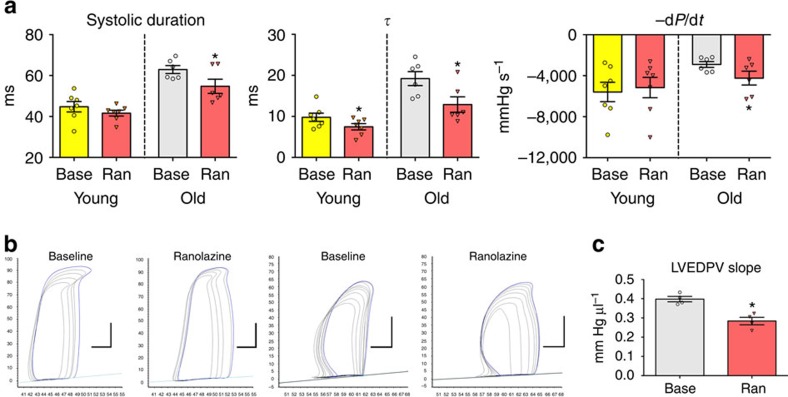
*I*_NaL_ alters LV function. (**a**) Quantitative data for LV haemodynamic parameters obtained in male mice at 5–6 months (Young) and at 29–33 months (Old) at baseline (Base) and ∼10 min following bolus infusion of ranolazine (2.5–5 mg per kg body weight, i.v.) (Young, *n*=7; Old *n*=6). Data are shown as mean±s.e.m. and scatter plots. *P<0.05 versus Base (paired *t*-test). Additional haemodynamic parameters and control experiments with saline infusion are reported in Supplementary Fig. 26. (**b**) PV loops during inferior vena cava occlusion protocol obtained in two male mice at 27 months at baseline and after bolus infusion of ranolazine (2.5 mg per kg body weight, i.v.). Scale bars, 3 μl, 20 mm Hg. (**c**) Quantitative data for LVEDPV slope obtained in male mice at 27 months at baseline (Base) and following bolus infusion of ranolazine (*n*=4). Data are shown as mean±s.e.m. and scatter plots. *P<0.05 versus Base (paired *t*-test).

To determine whether the improved muscle kinetics following inhibition of *I*_NaL_ had functional consequences on cardiac performance *in vivo*, LV haemodynamics were assessed in young and old mice prior to and after inhibition of *I*_NaL_, using an i.v. infusion protocol (Supplementary Fig. 26a). Blockade of the *I*_NaL_ with ranolazine in old mice led to faster contractile dynamics as documented by a shortened systolic duration and time constant of pressure decay, and increased −*d*P/*d*t (Fig. 9a and Supplementary Fig. 26b). These effects were attenuated in young animals. Importantly, *in vivo* infusion of ranolazine reduced the slope of the LVEDPV relationship in aged mice (Fig. 9b,c). Thus, *I*_NaL_ modulates the contractile and diastolic properties of cardiomyocytes, LV myocardium and whole heart.

### Molecular determinants of myocyte aging

**Figure 10:**
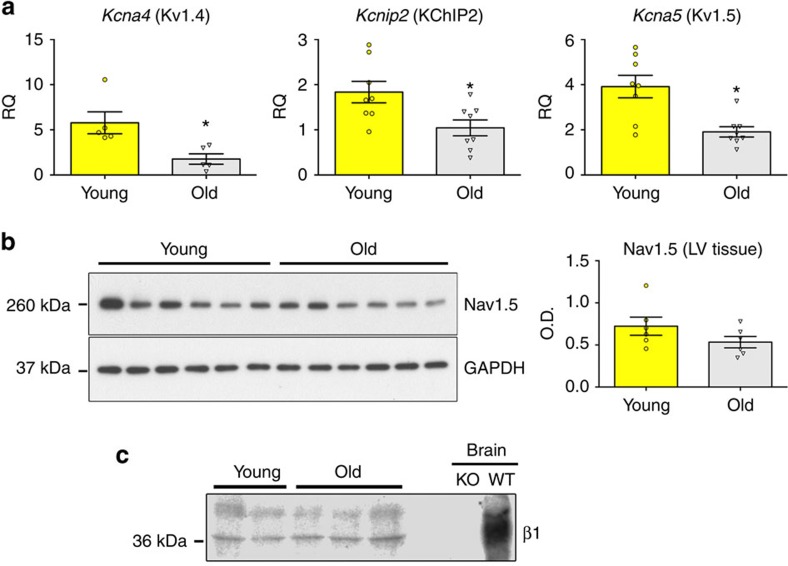
Aging and the expression of transcripts and proteins for K^+^ and Na^+^ channel subunits. (**a**) Quantitative data for the expression of genes related to channels mediating *I*_to_ (*Kcna4*, *Kcnip2*) and *I*_K,slow1_ (*Kcna5*) in myocytes from mice at 3–4 months (Young, *n*=5–8) and 27–33 months (Old, *n*=5–8) are shown as mean±s.e.m. and scatter plots. RQ, relative quantity with respect to β-2-microglobulin; *P<0.05 versus Young (Student’s *t*-test and Mann–Whitney rank sum test); NP, non-parametric analysis. (**b**) Expression of the Na^+^ channel protein subunit Nav1.5 by western blotting in the LV myocardium of mice at 3–5 months (Young, *n*=6) and 28 months (Old, *n*=6). GAPDH is the loading condition. Quantitative data are reported as mean±s.e.m. and scatter plots. (**c**) Expression of Na^+^ channel β1 subunit protein by western blotting in the LV myocardium of mice at 3–4 months (Young) and 30–31 months (Old). β1 levels in the brains of *Scn1b* null (KO) and wild-type (WT) mice were used as negative and positive controls, respectively. A total of 150 μg of LV protein lysate per lane and 25 μg of brain membrane protein per lane were loaded.

The gene expression for K^+^ and Na^+^ channels was evaluated in isolated preparations of young and old cardiomyocytes. The transcripts of *Kcnd2*, *Kcnd3* and *Kcnb1* genes, which encode, respectively, the K^+^ channel subunits Kv4.2, Kv4.3 and Kv2.1 were not affected by aging (Supplementary Fig. 27a). In contrast, the mRNAs of the *Kcna4*, *Kcna5* and *Kcnip2* genes, which encode the Kv1.4 and Kv1.5 channel subunits, and the Kv channel-interacting protein 2 or KChIP2, were decreased in old myocytes (Fig. 10a). These changes at the mRNA level are consistent with the attenuated density of Kv currents measured electrophysiologically (see Fig. 3b).

The mRNA levels of the *Scn5a*, *Scn1b*, *Scn2b* and *Scn4b* genes, which encode, respectively, the Na^+^ channel α-subunit Nav1.5 and the Na^+^ channel subunits β1, β2 and β4 did not differ significantly in young and old myocytes (Supplementary Fig. 27b). Transcripts of the isoforms of the Na^+^ channel α-subunit, typically found in the central nervous system (Nav1.1, Nav1.3, Nav1.6) and skeletal muscle (Nav1.4) were barely detectable or were comparable in young and old cardiomyocytes (Supplementary Fig. 27b). Similarly, the expression of Nav1.5 protein was preserved with age, while the levels of the β1 subunit, which modulates Na^+^ channel gating^40^,^41^,^42^, were barely detectable, with no measurable differences between young and old samples (Fig. 10b,c and Supplementary Fig. 27c). These findings support the possibility that post-translational modifications or alterations of the Na^+^ channel multiprotein complex may have contributed to the alteration of *I*_NaL_ with age^18^,^25^,^43^,^44^,^45^,^46^.

Quantitative reverse transcription–PCR (qRT–PCR) was then employed to define the expression of Ca^2+^ handling molecules in cardiomyocytes. Transcripts of the *Cacna1*, *Slc8a1*, *Ryr2* and *Pln* genes, which encode, respectively, the Ca^2+^ channel α-subunit Cav1.2, NCX, ryanodine receptor type-2 and phospholamban, were comparable in young and old myocytes. However, the mRNA of the *Atpa2a2* gene, encoding SERCA2a, was reduced in old cardiomyocytes (Supplementary Fig. 28). However, this change was no longer present at the protein level (Supplementary Fig. 29). Thus, the Ca^2+^ handling machinery is largely preserved in aging cardiomyocytes, strengthening the notion that defects in the electrical properties of these cells contribute in part to the abnormalities in Ca^2+^ cycling identified in the old heart.

## Discussion

The results of the present study document that myocardial aging is characterized by a slower electrical repolarization and protracted kinetics of contraction, which contribute to the alterations in the contractile and relaxation function of the old heart. The mechanisms involved in the modifications of ventricular compliance have been predominantly ascribed to collagen accumulation in the myocardial interstitium^1^,^4^,^6^,^47^. However, our findings indicate that physiological aging is not associated with diffuse interstitial fibrosis and/or multiple foci of tissue scarring in the myocardium, strengthening the critical role that the remodelled electromechanical characteristics of myocytes have in the diastolic and systolic properties of the aged heart.

The animal model employed here reiterates some of the features of human myocardial aging^1^,^2^. In the absence of systemic hypertension, 24–25-month-old mice show impaired diastolic function; EF and LV end-diastolic volume are preserved, but an increase in LV weight occurs with age. In contrast, in older animals at 30–35 months of age, deterioration of systolic performance, cavitary dilation and myocardial hypertrophy become noticeable, and these changes appear to be independent from the alterations in the diastolic indices observed at earlier time points. The elevated end-diastolic pressure and the perturbations in LV filling found at ∼2 years of age are coupled with protracted electrical recovery, corroborating recent observations in patients where the long QT interval has been linked to diastolic dysfunction^48^. The prolonged QT interval may be caused by lengthening of the AP and/or by the electrical heterogeneity of the ventricular myocardium, characterized by transmural voltage gradients and dispersion of repolarization^49^. However, our data, in which local epicardial recording was combined with measurements in isolated myocytes, strongly suggest that the shape of the AP is a major determinant of the abnormal electrical recovery of the senescent heart.

Changes in autonomic regulation occur with age^15^,^16^, a factor that may have attenuated the cardiac contractile reserve of the old heart. EF is reduced at 30 months and complete autonomic blockade blunts this difference in conscious animals. However, it has no effects on the protracted electrical repolarization and impaired diastolic indices of ventricular haemodynamics collected in anaesthetized mice. Caution has to be exercised when data obtained in conscious and anaesthetized animals are compared, although both the *in vivo* and *ex vivo* results tend to suggest that defects in neurohumural regulation have modest consequences on the electrical and passive mechanical properties of the senescent myocardium.

Our findings support the notion that the protracted electrical recovery with aging contributes to the alterations in myocyte relengthening and diastolic stiffness of the myocardium. Reduction in the rapidly activating Kv currents and increase of *I*_NaL_ are the ionic substrates responsible for the prolongation of the AP in aging cells. But whether gene regulatory networks and/or the activity of post-translational modulators are modified in aged cardiomyocytes, impacting on K^+^ and Na^+^ channel mutiprotein complexes, remains to be determined^18^,^25^,^43^,^44^,^45^,^46^,^50^.

The expression of Kv channel subunits is decreased in the post-infarcted heart in rodents and this condition is associated with reduced outward K^+^ current, prolonged AP and increased Ca^2+^ transient amplitude^23^. Although limited information is available in diseased mouse models with altered Na^+^ channel levels, genetic deletion of *Scn1b* in mouse cardiomyocyte results in longer APD, increased Ca^2+^ transients and slower Ca^2+^ decay; these defects, however, are reversed by low dose of TTX^51^. These observations and the current data in aging mice are consistent with the critical role that Kv and Na^+^ regulatory channel subunits have in modulating the electrical and mechanical properties of cardiomyocytes.

Reduced outward Kv currents and enhanced *I*_NaL_ contribute to the electrical recovery of old myocytes by altering, respectively, the early and late repolarization phase of the AP. Inhibition of *I*_NaL_ in old myocytes critically shortens APD90 to values found in young cells. However, this pharmacological intervention has minimal effects on the initial repolarization phase of the AP. Similarly, reduction of *I*_NaL_ *in vivo* reduces the duration of the QT interval in old mice restoring partly the electrical recovery of the myocardium. The negligible impact of ranolazine and mexiletine on the QT interval in young mice may reflect the attenuated density of *I*_NaL_ found in isolated cardiomyocytes.

In the failing heart, SERCA activity is attenuated and results in abnormal Ca^2+^ handling, which is centrally involved in the decline of cardiomyocyte performance^52^. However, SERCA protein is preserved in aging myocytes, suggesting that the cellular mechanisms implicated in the aging myopathy may be distinct from those typically found in chronic heart failure^52^. Although a potential role of SERCA with defective Ca^2+^ re-uptake has to be considered in the development of the decompensated aging heart, the increase in *I*_NaL_, together with the modifications in the electrical properties of cardiomyocytes, has to be viewed as an important variable of the progressive alterations in contractile kinetics and deterioration in diastolic and systolic properties of the senescent muscle.

Our results indicate that enhanced *I*_NaL_ mediates protracted repolarization of the AP and Na^+^ influx, potentiating the amplitude of Ca^2+^ transients and exerting a positive inotropic effect. Similarly, the decrease in Kv currents favours the prolongation of the AP promoting Ca^2+^ influx via L-type channels and reduced Ca^2+^ efflux via NCX^8^,^9^,^10^,^11^. Yet, the amplitude of Ca^2+^ transients and cell contractility are maintained with aging, in spite of prolonged APD, suggesting that the cellular defects in old myocytes condition the efficiency of the positive APD-inotropic relationship, which may be in part attributed to the reduced SR Ca^2+^ load. Thus, the changes in *I*_NaL_ and Kv currents may be interpreted as necessary responses aiming at the preservation of systolic function at the expense of a loss in cellular and ventricular compliance.

The prolonged AP in aging myocytes correlates with the delayed peak of Ca^2+^ transients and cell shortening, indicating that the slower repolarization phase sustains the Ca^2+^-induced Ca^2+^ release mechanism, increasing the translocation of Ca^2+^ from the SR to the cytoplasm. The ionic events underlying the positive inotropic action of enhanced *I*_NaL_ and the protracted AP may comprise an increased Na^+^ influx and enhanced [Na^+^]_i_ (ref. 41). The higher intracellular Na^+^ load is balanced by an increase in intracellular Ca^2+^ since the elevated Na^+^ attenuates the extrusion of Ca^2+^ via the NCX^9^,^37^,^41^. This form of inotropic support of myocyte contractility appears to be implicated in the complex adaptation of the myocardium to chronological aging. *I*_NaL_ may have a dual effect: it prolongs the AP and it influences indirectly the Ca^2+^ load. These variables affect, on the one hand, systolic Ca^2+^ release and contraction and, on the other, diastolic Ca^2+^ clearance and relaxation.

The central role of Na^+^ influx and enhanced Na^+^ load on the protracted Ca^2+^ transient decay in aged myocytes is corroborated by the findings in which the AP was prolonged by inhibition of Kv currents or enhancement of *I*_NaL_. Both approaches led to an increase in Ca^2+^ transient amplitude, but differentially modulated the timing of Ca^2+^ decay. Similarly, these interventions had a positive inotropic action on papillary muscles, but differently affected contractile dynamics: twitch kinetics were shortened with 4-AP, but were not altered with ATX-II. Importantly, when [Na^+^]_i_ was stabilized using cells dialysis and patch-clamp, differences in Ca^2+^ transient decay between young and old cells were abrogated. The faster caffeine-induced Ca^2+^ transient decay observed in old myocytes is suggestive of an increased NCX activity, which may reflect an adaptation to the altered Na^+^ load.

Inhibition of *I*_NaL_ in old myocytes shortens the AP and corrects the kinetics of Ca^2+^ transients, cell contraction and relaxation. Similarly, the repolarization and compliance of the senescent myocardium are restored, but muscle developed tension is attenuated. *I*_NaL_ modulates both the systolic and diastolic properties of the myocardium. However, in old mice, inhibition of *I*_NaL_ *in vivo* ameliorated diastolic indices and shortened systolic duration without interfering with developed pressure. Whether the blunted effects on developed pressure are due to compensatory adaptations inherent in the protocol employed requires further analysis. Thus, chronological age affects the electrical and molecular identity of cardiomyocytes, which are critical determinants of the pathological manifestations of the old heart.

## Methods

### Animals and *in vivo* studies

Male and female C57Bl/6 mice from 3 to 35 months of age were used in this study. When male or female mice were employed, gender is specified in the figure legend. Animals were maintained in accordance with the Guide for Care and Use of Laboratory Animals; animal experiments were approved by the local animal care committee (IACUC). When needed, isoflurane (1–1.5%, inhalation) was employed as a methodology of anaesthesia.

*Scn1b* WT and null littermate mice were generated and maintained in accordance with the guidelines of the University of Michigan Committee on the Use and Care of Animals. Mice were bred from *Scn1b* heterozygous animals that had been repeatedly backcrossed to C57Bl/6 mice for at least 18 generations, creating a congenic strain. Mice at postnatal day17 were anaesthetized and brains were removed for membrane preparations and subsequent western blot analysis, as previously done^51^.

In the attempt to define the effects of aging on LV function, anatomy and structure in mice, several assays were employed. The broad experimental approach was designed to obtain detailed information *in vivo* and to overcome limitations intrinsic to various experimental protocols and acquisition systems.

Blood pressure in conscious mice was analysed with a computerized, non-invasive tail-cuff plethysmography system (BP 2000 Blood Pressure Analysis System, Visitech Systems). For each mouse, 10 inflation–deflation cycles were used initially to set amplifier and instrument controls. Subsequently, systolic blood pressure was calculated by averaging readings from 40 cycles.

ECG was performed in conscious mice using a Visualsonics Vevo 2100 System equipped with a MS550D high frequency (22–55 MHz) linear transducer. By this approach, long and short axis views of the LV were analysed^32^. Diastolic function was assessed using pulsed-wave Doppler imaging of the transmitral filling pattern in the apical four-chamber view of the heart. Early transmitral filling wave (*E*-wave) and late filling wave due to atrial contraction (*A*-wave) were obtained. Isovolumic relaxation time was calculated as the time from closure of the aortic valve to the initiation of the *E*-wave, and isovolumic contraction time was calculated as the time from closure of the mitral valve to opening of the aortic valve. The deceleration time of the *E*-wave was determined by measuring the time needed for the down-slope of the peak of the *E*-wave to reach baseline^17^.

For high-frequency speckle-tracking imaging^17^, mice were anaesthetized and B-mode loops were recorded in the parasternal long- and short-axis views, while continuous ECG monitoring was obtained via limb electrodes. Three consecutive cardiac cycles were selected and analysed with Vevostrain software (Visualsonics). Strain measures were averaged over the cardiac cycles resulting in curvilinear strain and strain rates. In both the long- and short-axis views, the LV myocardium was divided into six standard anatomic segments, which were averaged for global speckle-tracking-based strain analysis throughout the cardiac cycle. Images collected with suboptimal physiological parameters or inadequate visualization of the endocardial border were excluded.

MRI was performed with a 7 T Pharmascan (Bruker) system equipped with a mouse cardiac coil in birdcage design (Rapid Biomedical) to obtain cine volumetric short axis^13^. To reduce motion artifacts and heart rate, animals were anesthetized with isoflurane by nose cone and placed into the coil in prone position. Electrodes were attached to the front left paw and right leg for electocardiographic gating and monitoring of vital signs using a gradient echo sequence (echo time 2.6 ms, 16 frames per RR interval; flip angle 30°; in-plane resolution 200 × 200 μm; slice thickness 1 mm). Animals were kept warm by blowing hot air into the magnet. The heat flow and the anaesthesia level were manually adjusted to maintain heart rate. Anatomical and functional parameters were quantitated from six to eight cine short axis imaging slices covering the LV.

ECGs were recorded under isoflurane anaesthesia by inserting needle electrodes s.c. into the mouse limbs (lead II). Electrical signals were amplified (Animal Bio Amp, ADInstruments), digitized using a 4 kHz A/D converter (MPVS-400, Millar Instruments) and recorded using LabChart software (ADInstruments), with low and high-pass filtering at 100 Hz and 3 Hz, respectively. Surface ECG intervals were measured using LabChart7 or LabChart8. Spontaneous cycle length was determined by averaging 10 consecutive RR intervals. PR and QT intervals were measured by determining the earliest onset and latest offset of atrial and ventricular deflections from the averaged cycles (Supplementary Fig. 30)^53^.

To record ECGs in freely roaming, unanaesthetized animals, telemetric biopotential transmitters (ETA-F10, Data Science International) were implanted surgically in anaesthetized mice^54^. Surgery was performed in anaesthetized mice. The peritoneum was exposed with a midline incision, the implant was secured within the peritoneal cavity, and leads were implanted s.c. corresponding to position II. Subsequently, signals were transmitted from implants to RPC-1 receivers and Data Exchange Matrix (Data Science International), and stored in a computer. The telemetric ECG was analysed during normal activity and following pharmacological treatments. All recordings were digitized at 2 kHz and analysed off-line with Ponemah 5.10 software.

LV haemodynamics and PV loops were obtained in anaesthetized mice (isoflurane, 1.5%) in the closed chest preparation with a MPVS-400 system for small animals equipped with a PVR-1045 catheter^32^,^55^,^56^,^57^,^58^. The mouse was intubated and ventilated (MiniVent Type 845, Hugo Sachs Elektronik-Harvard Apparatus) with isoflurane anaesthesia and warmed with a heat lamp; the right carotid artery was then exposed and the pressure transducer was inserted and advanced in the LV cavity. Data were acquired with Chart 5 or LabChart8 software. Baseline PV loops and loops following inferior vena cava or aortic arch occlusions were collected (Supplementary Fig. 31) to compute the slope of end-diastolic PV relations, which is an indicator of LV stiffness^57^. During data collection, ventilation was interrupted to acquire signals without motion artifacts. Inferior vena cava occlusion was achieved in the closed chest preparation by compression of the inferior vena cava accessed immediately below the diaphragm via a small abdominal incision, with a cotton tip applicator^58^. For aortic arch occlusion, a thoracotomy through the sternoclavicular articulation provided exposure of the aortic arch allowing its transient constriction with a bulldog clamp^59^. For calibration of PVR-1045 catheter and evaluation of LV blood volume, manufacturer’s instructions were followed. Cuvette calibration with fresh heparinized, warm blood and *in vivo* bolus infusion of hypertonic saline solution were performed to compute slope and intercept and to assess parallel conductance. Analysis was performed using LabChart and PVAN (Millar) software. Systolic duration was computed by LabChart as the elapsed time between the start of the cycle at LV end-diastolic pressure and the time of maximal −*d*P/*d*t.

For combined autonomic blockade, atropine (0.5 mg per kg body weight, i.p., Hospira) plus propranolol (1 mg per kg body weight, i.p., Fluka) were administered to animals^14^,^60^,^61^. *In vivo* blockade of the late Na^+^ current (*I*_NaL_) was achieved with administration of ranolazine (30 mg per kg body weight, i.p. or 2.5–5 mg per kg body weight, i.v., Sigma)^62^ or mexiletine (5 mg per kg body weight, i.p., Sigma)^63^. Compounds were dissolved in United States Pharmacopeia (USP) saline solution. Ranolazine bolus was delivered via left jugular vein using a syringe pump (PHD Ultra, Harvard Apparatus).

### *Ex vivo* properties of the mouse heart

To assess *ex vivo* electrical properties hearts were perfused through the aorta in a Langendorff apparatus (Radnoti) at a constant pressure of 80 mm Hg with Krebs–Henseleit buffer (KHB; Sigma), containing in mM: 118 NaCl, 4.7 KCl, 11 glucose, 1.2 MgSO_4_, 1.2 KH_2_PO_4_, 1.8 CaCl_2_ and 25 NaHCO_3_, gassed with 95% O_2_ and 5% CO_2_ (pH 7.4) at 37 °C (refs 12, 64). The temperature was maintained by immersing the heart in a water-heated glassware reservoir (Radnoti), containing preheated KHB. Hearts were stimulated with a 2-ms square pulse at 1.5-fold its threshold level (four channels stimulator, BMS 414, Crescent Electronics), using a mini-coaxial electrode (Harvard Apparatus). A two-lead mini ECG system (Harvard Apparatus), in which electrodes were placed on the right atrium and apex of the heart, was used to obtain pseudo-ECG. MAPs were recorded using a micro MAP-Tip electrode (Harvard Apparatus) positioned on the LV free wall^12^,^64^. ECG and MAP signals were amplified (Animal Bio Amp), digitized by a 4 kHz A/D converter (PowerLab 8/30, ADInstruments) and recorded with LabChart software, with low and high-pass filtering at 1 kHz and 0.3 Hz, respectively. A protocol of PES was introduced to assess the propensity of the mouse heart to develop ventricular arrhythmia. PES consisted of a train of pacing stimuli (S1) applied at 125 ms cycle length, with an extra stimulus (S2) inserted every eight beats. The S1–S2 interval was progressively reduced until the S2 stimulus either failed to generate an AP or induced arrhythmic events. The appearance of ventricular tachycardia (three or more consecutive ectopic beats characterized by atria-ventricular dissociation) and/or ventricular fibrillation was established^12^,^64^. Data were analysed with LabChart software.

### Myocyte isolation

Hearts were excised and LV myocytes were enzymatically dissociated using a methodology that was previously employed^11^,^12^,^32^,^55^,^56^. The heart was connected to a plastic cannula for retrograde perfusion through the aorta in a Langendorff system (Radnoti) at 37 °C. Perfusate consisted of a Ca^2+^-free solution gassed with 85% O_2_ and 15% N_2_. After 5 min, 0.1 mM CaCl_2_, 274 U ml^−1^ collagenase (type-2, Worthington Biochemical Corp) and 0.57 U ml^−1^ protease (Type XIV, Sigma) were added to the solution, which contained in mM: NaCl 126, KCl 4.4, MgCl_2_ 5, HEPES 5, Glucose 22, Taurine 20, Creatine 5, Na Pyruvate 5 and NaH_2_PO_4_ 5 (pH 7.4). At completion of digestion, atria and right ventricle were dissected and LV myocardium was cut in small pieces and these fragments were shaken in re-suspension solution and filtered using a 200 μm nylon mesh (Spectrum Labs). Aliquots of cell suspensions were centrifuged for 5 min at 100*g* (Eppendorf 5702R) at 4 °C and frozen for biochemical assays and fixed in paraformaldehyde (4%, Electron Microscopy Sciences). For electrophysiological and mechanical studies, only rod-shaped myocytes exhibiting cross striations, and showing no spontaneous contractions or contractures were selected; cells were used within 8 h following enzymatic digestion.

### Myocyte volume

LV myocytes fixed in paraformaldehyde were stained with the nuclear dye Hoechst (10 μM, Sigma). Images were collected with a fluorescent inverted microscope (Olympus IX71) equipped with a CCD camera (Hamamatsu ORCA-R2). Cell length and area were evaluated using ImageJ software. Myocyte volume was then computed assuming an elliptical cross-section, in which the major axis corresponds to the average cell width, and the minor axis was computed after establishing the ratio between the major and minor axis, using three-dimensional optical section reconstruction by two-photon microscopy (BX51WI Olympus microscope coupled with a Bio-Rad Radiance 2100MP system) and image analysis (ImageJ). Three-dimensional reconstructions were obtained using second-harmonic generation signal of sarcomeric structures^65^.

### Histological analysis

Mouse hearts were fixed in phosphate-buffered formalin (10%, Sigma) and embedded in paraffin. Sections 4-μm thick of the LV were trichrome-stained for detection of connective tissue (Gomori’s One Step Trichrome Method, Poly Scientific R&D Corp.) following manufacturer’s instructions. Images were acquired using an upright microscope (Olympus BX60) with × 20 objective equipped with a colour camera (Olympus DP73). Interstitial fibrosis was quantified with respect to total tissue area using ImageJ software.

### Cell shortening and Ca^2+^ transients

Isolated LV myocytes were placed in a bath on the stage of an Axiovert (Zeiss), IX71 and BH-2 (Olympus) microscopes for contractility and Ca^2+^ transients^11^,^12^,^32^,^55^,^56^. Experiments were conducted at room temperature. Cells were bathed continuously with a Tyrode solution containing (in mM) NaCl 140, KCl 5.4, MgCl_2_ 1, HEPES 5, Glucose 5.5 and CaCl_2_ 1.0 (pH 7.4, adjusted with NaOH). Measurements were collected in field-stimulated (S88 and SD9 Grass stimulators) cells by IonOptix fluorescence and contractility systems (IonOptix), or by video edge detection (VED-205, Crescent Electronics; PowerLab 8/35, ADInstruments). Contractions were elicited by rectangular depolarizing pulses, 2 ms in duration and 1.5 times threshold in intensity, with platinum electrodes. Ca^2+^ transients were measured by epifluorescence after loading the myocytes with 10 μM Fluo-3 AM or 2 μM Fluo-4 AM (Invitrogen). Excitation length was 480 nm with emission collected at 535 nm using a × 40 objective. Fluo signals were expressed as normalized fluorescence (*F*/*F*_0_), where *F*_0_ is the diastolic fluorescent level subtracted by the background signal measured in the region adjacent to the cell^12^.

Unless otherwise specified, the late Na^+^ current (*I*_NaL_) was blocked with a low dose of ranolazine (10 μM, Sigma) or mexiletine (10 μM, Sigma), or enhanced with anemonia toxin-II (1 nM, Sigma)^25^,^26^,^28^,^29^,^39^,^66^,^67^,^68^. Rapidly activating Kv outward currents were blocked with 0.5 mM 4-AP (Sigma)^11^,^22^,^24^.

### Caffeine-induced Ca^2+^ transients

To evaluate SR Ca^2+^ load in field-stimulated myocytes, a 2 s caffeine (20 mM, Sigma) spritz was delivered using glass pipettes and a microinjector (FemtoJet, Eppendorf AG, or Pico-liter Injector, Warner Instruments), and Ca^2+^ transients recorded by epifluorescence microscope and IonOptix system, or two-photon microscope^11^,^12^,^32^,^55^. For two-photon imaging, myocytes were loaded with 5 μM Fluo-4 AM and placed on the stage of an upright microscope (BX51WI Olympus microscope coupled with a Bio-Rad Radiance 2100MP system). Cells were bathed with Tyrode solution and field stimulated using platinum electrodes. Fluo-4 was excited at 920 nm wavelength with mode-locked Ti/sapphire femtosecond laser (Tsunami, Spectra-Physics), and the emission signal was collected at 535 nm. Images were acquired in line scan mode with the myocyte oriented in the long axis, at 6 ms sampling rate^12^,^55^. Fluo signals were expressed as normalized fluorescence (*F*/*F*_0_), where *F*_0_ is the diastolic fluorescent level subtracted by the background signal measured in the region adjacent to the cell^12^. Due to potential leak of caffeine from the glass pipette and undesired facilitation of Ca^2+^ release, cells displaying paced-induced Ca^2+^ transients larger than 5 *F*/*F*_0_ (corresponding to values above average+2·s.d.) were excluded from the analysis.

Na^+^/Ca^2+^ exchange (NCX) activity was evaluated by the decay of the caffeine-induced Ca^2+^ transient, which was fitted with a mono-exponential function^33^,^69^. Cells presenting prolonged Ca^2+^ transient decay (>4.8 s, corresponding to values >average+2·s.d.) and/or poor fitting (R^2^<0.96) were excluded from the study, without affecting statistical results.

### Whole-cell patch-clamp studies

Isolated LV myocytes were placed in a bath on the stage of an IX51, IX53, and IX71 microscopes for patch-clamp measurements^11^,^12^,^32^,^55^,^56^,^66^. Experiments were conducted at room temperature. Data were acquired by means of the whole-cell patch-clamp technique in voltage- and current-clamp modes using Multiclamp 700A and 700B, and Axoclamp 900A amplifiers (Molecular Devices). Electrical signals were digitized using 250 kHz 16-bit resolution A/D converters (Digidata 1322, 1440A, and 1550, Molecular Devices) and recorded using pCLAMP 9.0 and 10 software (Molecular Devices) with low-pass filtering at 2 kHz. Membrane capacitance (*C*_m_) was measured in voltage-clamp mode using a 5 mV voltage step and pCLAMP software algorithm. For each cell, current density was obtained by dividing transmembrane currents recorded in voltage-clamp by the measured *C*_m_, providing a normalization of ionic currents with respect to cell size. Current density was expressed in pA pF^−1^ (refs 11, 12, 32, 55, 56). Pipettes were pulled by means of a vertical (PB-7, Narishige), or horizontal (P-1000, Sutter Instrument) glass microelectrode pullers; when filled with intracellular solution pipettes had a resistance of 1–2 MΩ.

### Action potential measurements

For AP measurements, current-clamp mode was employed^11^,^12^,^66^. Cells were stimulated with current pulses 1.5 times threshold. Myocytes were bathed with Tyrode solution. The composition of the pipette solution was (in mM): NaCl 10, KCl 113, MgCl_2_ 0.5, K_2_-ATP 5, glucose 5.5, HEPES 10, EGTA 10 and CaCl_2_ 1 (pH 7.2 with KOH). To test the effects of ion channels modulators on the electrical properties of myocytes, APs were continuously recorded for the same cell before and after exposure to agonist or inhibitors. The following compound were used in current-clamp experiments: ranolazine (10 μM), mexiletine (10 μM), anemonia toxin-II (1 nM)^25^,^26^,^28^,^29^,^39^,^66^,^67^,^68^ and 4-AP (0.5 mM)^11^,^22^,^24^.

### Measurements of voltage-gated K^+^ currents

Voltage-gated outward K^+^ Kv currents were assessed in voltage-clamp mode with a previously reported protocol^24^, to dissect K^+^ currents with rapid activation and fast (*I*_to_), intermediate (*I*_K,slow1_) and slow (*I*_K,slow2_) kinetics of inactivation, together with a sustained (non-inactivating) component (*I*_ss_)^21^,^24^. Cells were bathed with a modified Tyrode solution containing (in mM) NaCl 140, KCl 4, MgCl_2_ 1, HEPES 10, Glucose 10, CaCl_2_ 1.2 and 0.3 mM CdCl_2_ to block L-type Ca^2+^ current (pH 7.4, adjusted with NaOH)^11^,^24^. The composition of the pipette solution was (in mM): NaCl 5, KCl 20, K-aspartate 120, MgCl_2_ 1, Mg-ATP 5, EGTA 10, HEPES 10 (pH 7.2 with KOH)^24^. Following membrane capacitance and series resistance compensation, Kv currents were activated using depolarizing voltage steps from −80 mV to +60 mV, 25 s in duration. Interpulse interval was 60 s. *I*_to_, *I*_K,slow1_, *I*_K,slow2_ and *I*_ss_ amplitude and time constants were obtained by fitting the current decay during the depolarizing step with a tri-exponential function using Clampfit 10 software^24^. Currents were normalized by *C*_m_.

### Measurements of voltage-gated Na^+^ currents

The late Na^+^ current (*I*_NaL_) was measured in voltage-clamp mode^25^,^67^,^68^. Myocytes were bathed with a modified Tyrode solution in which KCl was replaced with CsCl and 4 μM nicardipine was added to block L-type Ca^2+^ current. Composition of the pipette solution was (in mM): NaCl 10, CsCl 113, MgCl_2_ 0.5, Tris-ATP 5, glucose 5.5, HEPES 10, EGTA 5 and tetraethylammonium chloride 20 (pH 7.2 with CsOH). Myocytes were held at *V*_h_ of −120 mV, and *I*_NaL_ was elicited using 500-ms depolarizing pulses to −30 mV. The pulse was preceded by a 5 ms prepulse to +50 mV to optimize voltage control^67^. Interpulse interval was 5 s. *I*_NaL_ was measured as inward current decaying during the 500-ms depolarizing step to −30 mV, excluding the initial 20 ms of the depolarizing step. *I*_NaL_ was normalized by *C*_m_. *I–V* relations were determined applying depolarizing steps 500 ms in duration from *V*_h_ −120 mV in 5 mV increments. The family of depolarizing steps was preceded by a 20-ms preconditioning step to −50 mV to activate the fast Na^+^ current. Interpulse interval was 5 s. *I*_NaL_ amplitude was measured as inward current decaying during the 500-ms depolarizing at various test potentials, excluding the initial 20 ms. *I*_NaL_ was normalized by *C*_m_.

Equilibrium potential for Na^+^ at 25 °C was calculated by the Nernst equation:




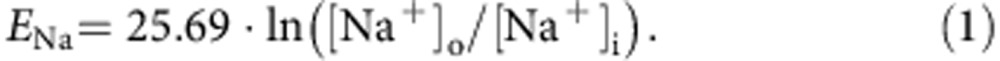




At each potential tested (*V*_m_), Na^+^ conductance (*g*) was calculated as




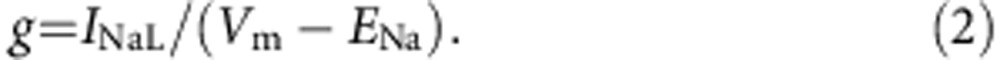




where *I*_NaL_ is the amplitude of the late Na^+^ current at *V*_m_.

Conductance–voltage relations were plotted and fitted with Boltzmann function:




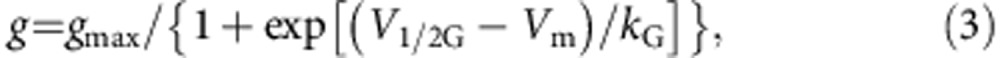




where *g*_max_ corresponds to maximal conductance, *V*_1/2G_ the potential at which conductance is halfway between 0 and *g*_max_ and *k*_G_ the slope of the conductance curve.

Values of normalized Na^+^ conductance (*G*=*g* · *g*_max_^−1^) were plotted to obtain activation curves; half maximal activation potential (*V*_1/2G_) and slope of the activation curve (*k*_G_) were derived by fitting the data with Boltzmann equation:




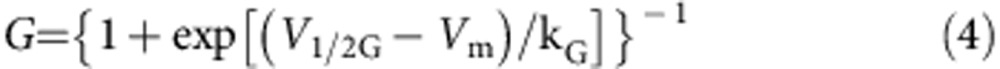




A two-pulse protocol was utilized to assess the voltage dependence of steady-state inactivation of *I*_NaL_. Prepulses were introduced to depolarize the cell to different membrane voltages ranging from −120 to +50 mV for 500 ms, in 10-mV increments. Each prepulse was followed by a single 500-ms test pulse, which depolarized the cell to −30 mV. Different prepulses were applied at 5-s intervals. Values of normalized Na^+^ conductance (*G*=*g* · *g*_max_^−1^) were plotted with *V*_m_ relative to the preconditioning steps to determine steady-state inactivation curves, fitted with a Boltzmann equation, as indicated above (equation (4)).

In addition, *I*_NaL_ was assessed as TTX-sensitive current using depolarizing steps to −40 mV, 520 ms in duration from holding potential of −80 mV. The protocol was recorded for the same cell in Tyrode solution and following perfusion with 10 μM TTX (Sigma, Enzo Life Sciences). Scout depolarizations 10 mV in increments from *V*_h_ −80 mV and 5-s interpulse interval were applied every 1–2 min to evaluate the effects of TTX and experimental conditions. Difference current was obtained by subtracting traces recorded in TTX from those measured in Tyrode. The slowly decaying TTX-sensitive current was evaluated by quantifying the inward current decaying over the 520-ms depolarization, excluding the initial 20 ms of the depolarizing step. Currents were normalized by *C*_m_. For these measurements, pipette solution was comparable to the one employed for AP measurements.

The fast Na^+^ current (*I*_Na_) was measure in voltage-clamp mode^70^. Myocytes were bathed with a modified Tyrode solution of the following composition (in mM): NaCl 5, MgCl_2_ 1, CaCl_2_ 1, CdCl_2_ 0.1, HEPES 20, glucose 11 and CsCl 132.5 (pH 7.4 with CsOH). The composition of the pipette solution was (in mM): NaCl 5, CsF 135, EGTA 10, Mg-ATP 5 and HEPES 5 (pH 7.2 with CsOH)^70^. *I–V* relations were determined applying depolarizing steps 200 ms in duration from *V*_h_ −120 mV in 5-mV increments. Interpulse interval was 3 s. *I*_Na_ amplitude was measured as the current difference between the inward peak current and the current at the end of the 200-ms step. *I*_Na_ was normalized by *C*_m_.

A two-pulse protocol was utilized to assess the voltage dependence of steady-state inactivation of *I*_Na_. Prepulses were introduced to depolarize the cell to different membrane voltages starting from −140 mV for 300 ms, in 5-mV increments. Each prepulse was followed by a single 30-ms test pulse, which depolarized the cell to −40 mV. Values of normalized Na^+^ conductance (*G*=*g* · *g*_max_^−1^) were plotted with *V*_m_ relative to the preconditioning steps to compute the steady-state inactivation curves, which were fitted with a Boltzmann equation, as indicated above (equation (4)).

*I*_Na_ reactivation was studied using a two-pulse protocol with variable inter-pulse duration, ranging from 100 to 5ms, in decrements of 5ms. With each pulse, *I*_Na_ was activated by depolarizing cells from *V*_h_ −100 mV to −20 mV for 20ms. The double pulse protocol was repeated every 3s. For each interpulse duration (*t*), *I*_Na_ reactivation was calculated by dividing the amplitude of the current measured during the second pulse (*I*_(*t*)_) by the amplitude of the current measured during the first pulse (*I*_max_). Values of *I*_Na_ reactivation were plotted to obtain reactivation curves and fitted with a mono-exponential function:




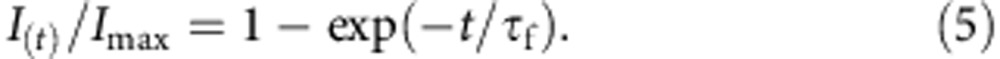




In voltage-clamp experiments in which Na^+^ currents were modulated pharmacologically with mexiletine (10 μM for quantification of the effects on *I*_Na_; 30 μM for qualitative effects on *I*_NaL_) or anemonia toxin-II (1 nM), depolarizing pulses between −60 mV and 0 mV, 10 mV in increments, 5-s interpulse interval, from *V*_h_ −70 mV or −90 mV (see legend to figure for details) were tested every 1–2 min to evaluate the effects of drugs and experimental conditions.

### Measurements of L-type Ca^2+^ current

The L-type Ca^2+^ current (*I*_CaL_) was measured in voltage-clamp mode^11^,^32^,^56^. Myocytes were bathed with a modified Na^+^–K^+^-free Tyrode solution of the following composition (in mM): N-methyl-D-glucamine (NMDG) 140, CsCl 4, MgCl_2_ 1, HEPES 5, glucose 5.5, CaCl_2_ 1 and 4-AP 2 (pH 7.4 with CsOH). The composition of the pipette solution was (in mM): NMDG 10, CsCl 113, MgCl_2_ 0.5, Tris-ATP 5, glucose 5.5, HEPES 10, EGTA 5 and TEA-Cl 20 (pH 7.2 with CsOH). *I*_CaL_ *I–V* relation, conductance and activation properties were determined applying depolarizing steps 300 ms in duration from *V*_h_ −70 mV in 10-mV increments. *I*_CaL_ amplitude was measured as the current difference between the peak inward current at the beginning of the step and the current at the end of the 300-ms pulse. *I*_CaL_ was normalized by *C*_m_.

Equilibrium potential for Ca^2+^ was defined by the intercept of the *I–V* relation to the zero-current axis. A linear fitting of the positive potential portion of the *I–V* relation was employed for this determination. At each potential tested (*V*_m_), Ca^2+^ conductance (*g*) was calculated as




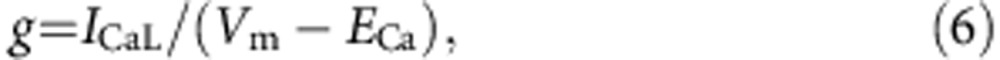




where *I*_CaL_ is the amplitude of the L-type Ca^2+^ current at *V*_m_.

Conductance–voltage relations were plotted and fitted with Boltzmann function (equation (3)). Values of normalized Ca^2+^ conductance (*G*=*g* · *g*_max_^−1^) were plotted to obtain activation curves; half maximal activation potential (*V*_1/2G_) and slope of the activation curve (*k*_G_) were derived by fitting the data with Boltzmann equation (equation (4)).

A two-pulse protocol was utilized to assess the voltage dependence of steady-state inactivation of *I*_CaL_. Prepulses were introduced to depolarize the cell to different membrane voltages starting from −80 mV for 300 ms, in 10-mV increments. Each prepulse was followed by a single 300-ms test pulse, which depolarized the cell to 0 mV. Values of normalized Ca^2+^ conductance (*G*=*g* · *g*_max_^−1^) were plotted with *V*_m_ relative to the preconditioning steps to determine steady-state inactivation curves, fitted with a Boltzmann equation, as indicated above (equation (4)).

Inactivation time course of *I*_CaL_ was studied with a 300-ms depolarizing pulse at 0 mV (ref. 11). Traces were fitted between the inward peak and the end of the 300-ms pulse to 0 mV with a bi-exponential function^66^:




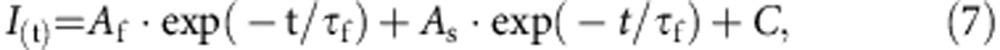




where *A*_f_ and *A*_s_ are, respectively, the fraction of the fast- and slow-inactivating components, and *C* is the offset constant.

### AP-clamp and Ca^2+^ transients

For AP-clamp experiments, Ca^2+^ levels in patch-clamped myocytes were assessed as fluorescence signal intensity of Fluo-loaded cells using a photomultiplier and a photon to voltage converter (IonOptix) connected to the patch-clamp A/D converter^11^,^12^. Cells were bathed in Tyrode solution and pipette solution was comparable to the one employed for AP measurements with the omission of EGTA and CaCl_2_.

Fluo signals were expressed as normalized fluorescence (*F*/*F*_0_), where *F*_0_ is the diastolic fluorescent level subtracted by the background signal measured in the region adjacent to the cell^12^. Duration of Ca^2+^ transient decay was evaluated at 10–90%, and obtained results from different cells were averaged and fitted with a mono-exponential function:




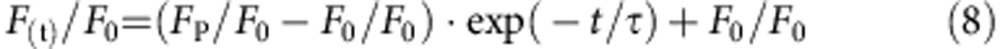




where *F*_P_ and *F*_0_ are, respectively, the fluorescence relative to the peak and diastolic phase of the Ca^2+^ transient.

### Isometric force in papillary muscles

Papillary muscles were dissected from the mouse LV and mounted in a horizontal tissue bath (Steiert, Hugo Sachs Elektronik-Harvard Apparatus) connected to a force transducer (F10, Harvard Apparatus)^12^. Muscles were superfused with KHB solution at 37 °C. The myocardium was stimulated by two platinum electrodes employing field stimulation (isolated stimulator output: frequency 6 Hz; pulse duration 2 ms; intensity 1.5-fold threshold; UISO, Hugo Sachs Elektronik-Harvard Apparatus). Each muscle was stretched to the length at which force of contraction was maximal. Muscle preparations were allowed to equilibrate for at least 30 min. Developed tension was measured isometrically with the force transducer attached to a Bridge Amp (ADInstruments) and a 4 kHz A/D converter (PowerLab 4/30, ADInstruments). Tension signal was recorded using LabChart 7 Pro software and analysed with LabChart8. Time to 50 and 90% relaxation are expressed with respect to the beginning of the twitch. Based on the premise that each muscle had a cylindrical shape, force measurements were normalized by the cross-sectional area of the muscle (mN mm^−2^). Digital images of sections were acquired using micro-zoom system microscopes (MVX10 and SZX16, Olympus) and a sCMOS camera (pco.edge) or a CCD camera (DP73), and analysed by ImageJ software^12^.

Passive diastolic tension was examined by progressively stretching muscles stimulated at 6 Hz, beginning from near slack length (*L*_0_) to the length at which the muscle developed maximal twitch tension (*L*_max_). Diastolic length–tension relations were plotted and fitted with a second-order polynomial equation:




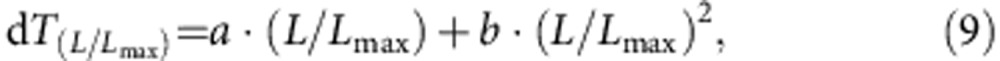




where d*T* is diastolic tension and *L* is the length of the muscle.

The late Na^+^ current was blocked with ranolazine (10 μM, Sigma) or mexiletine (10 μM, Sigma), or enhanced with anemonia toxin-II (10 nM, Sigma)^25^,^29^,^39^,^67^. 4-AP (1 mM, Sigma) was employed to inhibit Kv currents and induce prolongation of myocyte AP^11^,^22^,^24^. Twitch properties were evaluated in young and old muscles at baseline and following superfusion with agonists or inhibitors. Muscles exposed to excessive stretching and displaying unstable twitches were excluded from the analysis. For evaluation of diastolic tension-length relations, muscles were superfused with KHB, mexiletine or anemonia toxin-II, and then progressively stretched under continuous electrical stimulation.

### QRT–PCR

Total RNA was extracted from isolated mouse LV myocytes utilizing TRIZOL reagent (Invitrogen). Complementary DNA was obtained from 1 μg total RNA using MultiScribe reverse transcriptase kit (Applied Biosystems). Real-time RT–PCR was performed with primers designed using the Vector NTI Advance 11 (Invitrogen) software^12^,^55^,^64^. The sequences of primers are indicated in Supplementary Table 1. The StepOnePlus Real-Time PCR system (Applied Biosystems) was employed for quantitative RT–PCR. In each case, complementary DNA was combined with Power SYBR Green Master Mix (Applied Biosystems) in a 10-μl reaction. Cycling conditions were as follow: 95 °C for 10 min followed by 40 cycles of amplification (95 °C denaturation for 15 s, 60 °C annealing and extension for 1 min). The melting curve was then obtained. *C*_t_ values were normalized with respect to β-2-microglobulin (*β2m*). To avoid the influence of genomic contamination, forward and reverse primers for each gene were located in different exons. PCR products were run on 3% agarose/1 × TBE gel to confirm the specificity of the reaction. Total RNA extracted from mouse brain and skeletal muscle was employed as controls. For fibrotic markers^18^, RNA was extracted from LV tissue. *C*_t_ values were normalized with respect to hypoxanthine guanine phosphoribosyl transferase (*Hprt*)^18^.

### Western blotting

Whole protein extracts from pellets of isolated LV myocytes or LV myocardium were prepared using RIPA buffer (Sigma), supplemented with a cocktail of protease inhibitors (Roche) and phosphatase inhibitors (Sigma). Equivalent of 15 μg of proteins were separated on SDS–PAGE, transferred onto polyvinylidene fluoride membrane, blocked with 5% BSA and exposed to the antibodies listed in Supplementary Table 2. Dilution is also indicated in Supplementary Table 2. Horseradish peroxidase-conjugated secondary antibodies (Cell Signaling Technology, 1:5,000 dilution) and Pierce ECL 2 western Blotting chemiluminescent substrate (Thermo Scientific) were utilized for signal detection^12^. Western blotting protocols with various antibodies were optimized before quantitative analysis. For molecular weight identification, Precision Plus Protein Kaleidoscope Prestained Protein Standards (Bio-Rad) was employed. Optical density of bands was measured using ImageJ and normalized by the expression of GAPDH.

For Na^+^ channel β1 subunit protein levels, samples were mechanically homogenized and sonicated in Tris-EGTA buffer (50 mM Tris, 10 mM EGTA) (for LV samples) or in RIPA buffer (Thermo) plus octyl β-D-glucopyranoside (Sigma-Aldrich) and protease inhibitors, with a brief low speed centrifuge spin (5 min, 5,000*g*) to remove debris and loaded with the addition of SDS-sample buffer. Approximately 150 μg of LV or myocyte lysate and 25 μg of brain membrane protein per lane were loaded. Brain membrane protein from wild-type and *Scn1b* null mice^40^ were employed as positive and negative controls, respectively. To obtain membrane proteins, a protocol previously used was employed here^51^. Brain tissue was immersed in 2 ml of Tris-EGTA buffer with Complete protease inhibitors (Roche). The brain was homogenized using a polytron followed by a dounce homogenizer and then centrifuged at 2,500*g* for 10 min at 4 °C. The supernatant was then centrifuged at 100,000*g* in a Sorvall TFT 80.4 rotor for 55 min at 4 °C and the pellet resuspended and sonicated in Tris-EGTA plus protease inhibitors. Proteins were separated on 12% acrylamide SDS–PAGE gels, transferred onto nitrocellulose, probed overnight with a commercially available rabbit monoclonal Na^+^ channel β1 subunit antibody developed by Cell Signaling Technology for L.L.I. (D9T5B, 1:750 dilution), then probed with a goat anti-rabbit secondary antibodies conjugated to horseradish peroxidase (Thermo Scientific, 1:1,000 dilution). Blots were incubated with West Femto enhanced chemiluminescent substrate (Thermo Scientific) and imaged using a LI-COR Odyssey system (LI-COR Biotechnology). Full images of western blots are presented in Supplementary Figs 32–34.

### Data analysis

Data are presented as median and interquartile ranges or mean±s.e.m. Linear and nonlinear regressions were calculated with Prism 6.0c software, and parameters are reported in Supplementary Table 3. Statistical analysis was performed using SigmaPlot 11.0 software. Data were initially tested for normality (Shapiro–Wilk) and equal variance for assignment to parametric or non-parametric analysis. Parametric test included Student’s *t*-test or analysis of variance followed by Bonferroni test for non-paired comparison between two or among multiple groups, respectively. For paired statistical analysis, paired *t*-test or one-way repeated measures analysis of variance followed by Bonferroni test was employed for two groups or multiple comparisons, respectively. When normality or equal variance were not met, non-parametric analysis was performed using Mann–Whitney Rank sum test or Kruskal–Wallis one-way analysis of variance on ranks followed by Dunn’s method, for non-paired comparison between two or among multiple groups, respectively. Wilcoxon signed rank test or Friedman repeated measures analysis of variance on ranks was employed for paired comparison between two or among multiple groups, respectively. For categorical data analysis, Fisher’s exact or *χ*^2^-tests was used. *P*<0.05 was considered significant.

## Additional information

**How to cite this article:** Signore, S. *et al*. Late Na^+^ current and protracted electrical recovery are critical determinants of the aging myopathy. *Nat. Commun*. 6:8803 doi: 10.1038/ncomms9803 (2015).

## Supplementary Material

Supplementary Information Supplementary Figures 1-34 and Supplementary Tables 1-3
