# Supplementary material for: Late Na+ current and protracted electrical recovery are critical determinants of the aging myopathy
Source: Nat Commun. 2015 Nov 6;6:8803. doi: 10.1038/ncomms9803 (PMC4638135; doi:10.1038/ncomms9803)
Supplement: Supplementary Information — Supplementary Figures 1-34 and Supplementary Tables 1-3 [file ncomms9803-s1.pdf]

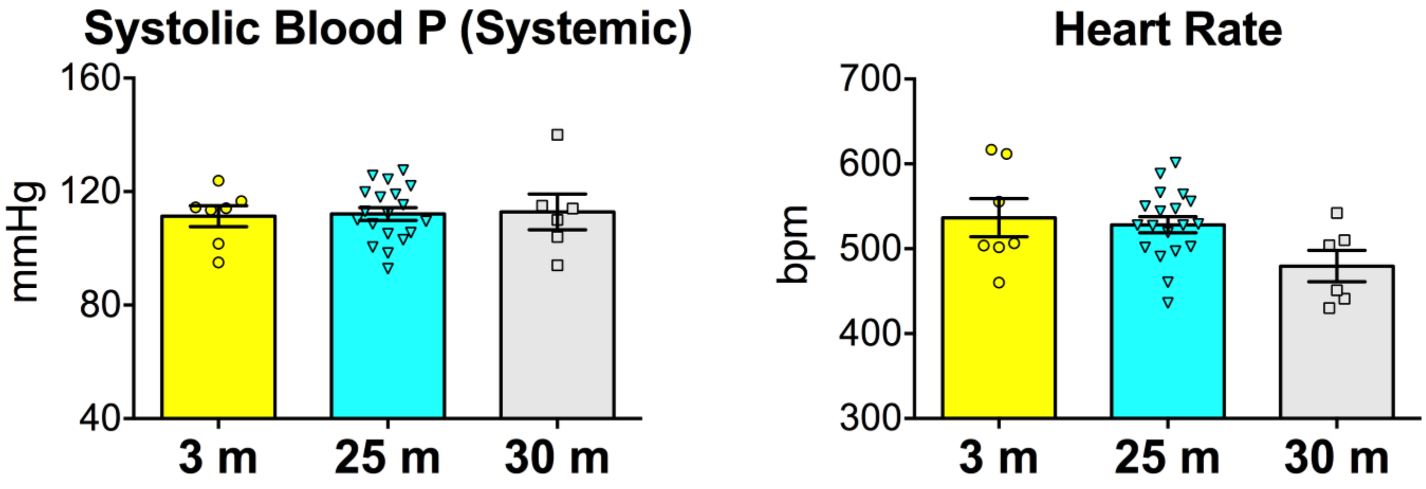

Supplementary Figure 1. Systemic pressure is preserved in aging mice. Quantitative data for non-invasive systolic blood pressure (left) and heart rate (right) evaluated in conscious male mice at 3 months (3 m, n = 7), 25 months (25 m, n = 19), and 30 months (30 m, n = 6) are shown as mean ± s.e.m. and scatter plots. Data was not significantly different (one-way ANOVA).

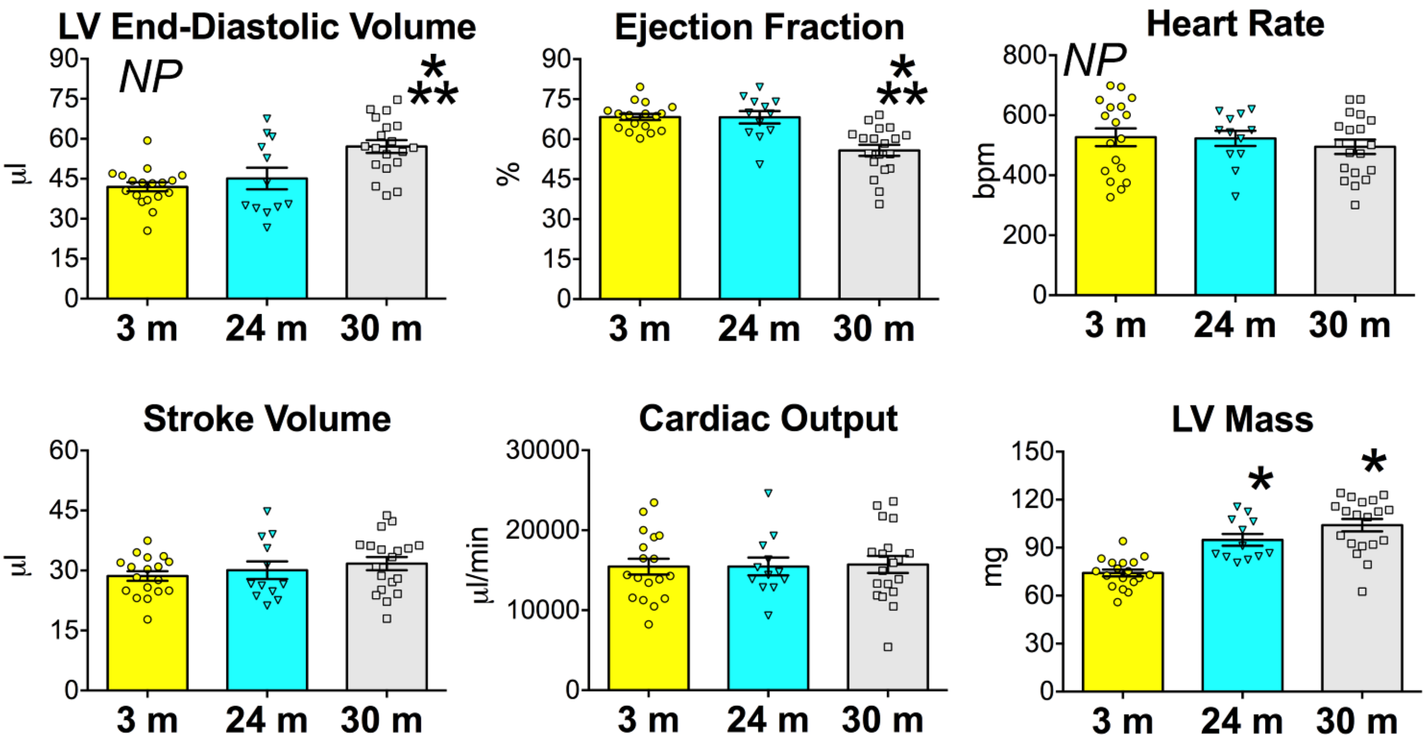

Supplementary Figure 2. Cardiac function is depressed in senescent mice. Anatomical and functional parameters evaluated by echocardiography in aging mice. Data for male mice at 3 months (3 m, *n* = 18), 24 months (24 m, *n* = 12), and 30 months (30 m, *n* = 19) are shown as mean ± s.e.m. and scatter plots. **P*<0.05 versus 3 m; ***P*<0.05 versus 24 m (one-way ANOVA with Bonferroni's *post hoc* test and Kruskal-Wallis one-way ANOVA on ranks with Dunn's *post hoc* test); *NP*: non-parametric analysis.

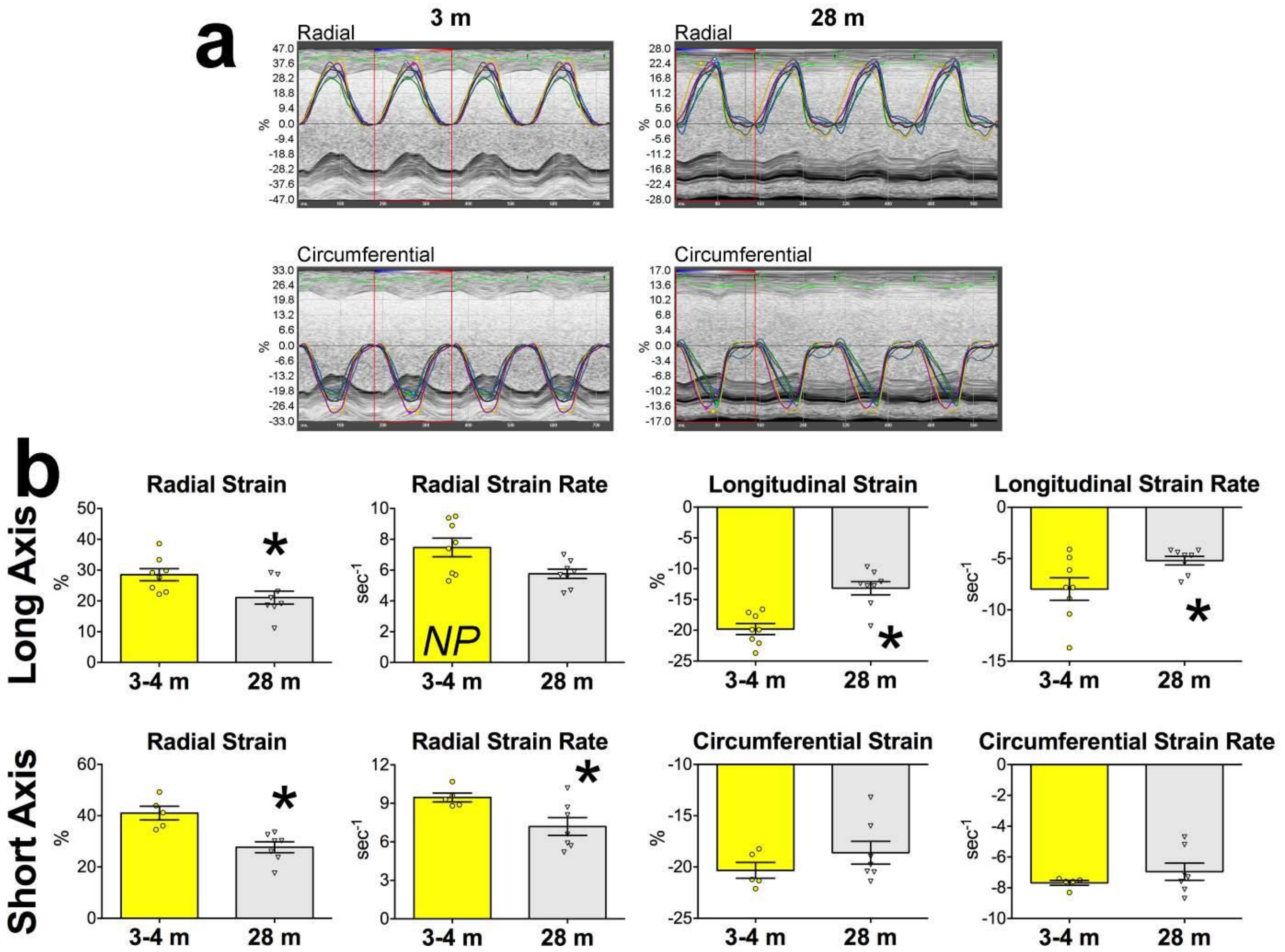

Supplementary Figure 3. Speckle tracking-based strain analysis for the evaluation of cardiac function in the aging heart. (**a**) Radial and circumferential strain curves for parasternal short axis images in male mice at 3 months (3 m) and 28 months (28 m) of age. (**b**) Quantitative data of speckle tracking-based analysis for male mice at 3-4 months (3-4 m, *n* = 5-8) and 28 months (28 m, *n* = 7-8) are shown as mean ± s.e.m. and scatter plots. *$P<0.05$ versus 3-4 m (Student's *t*-test and Mann-Whitney rank sum test); *NP*: non-parametric analysis.

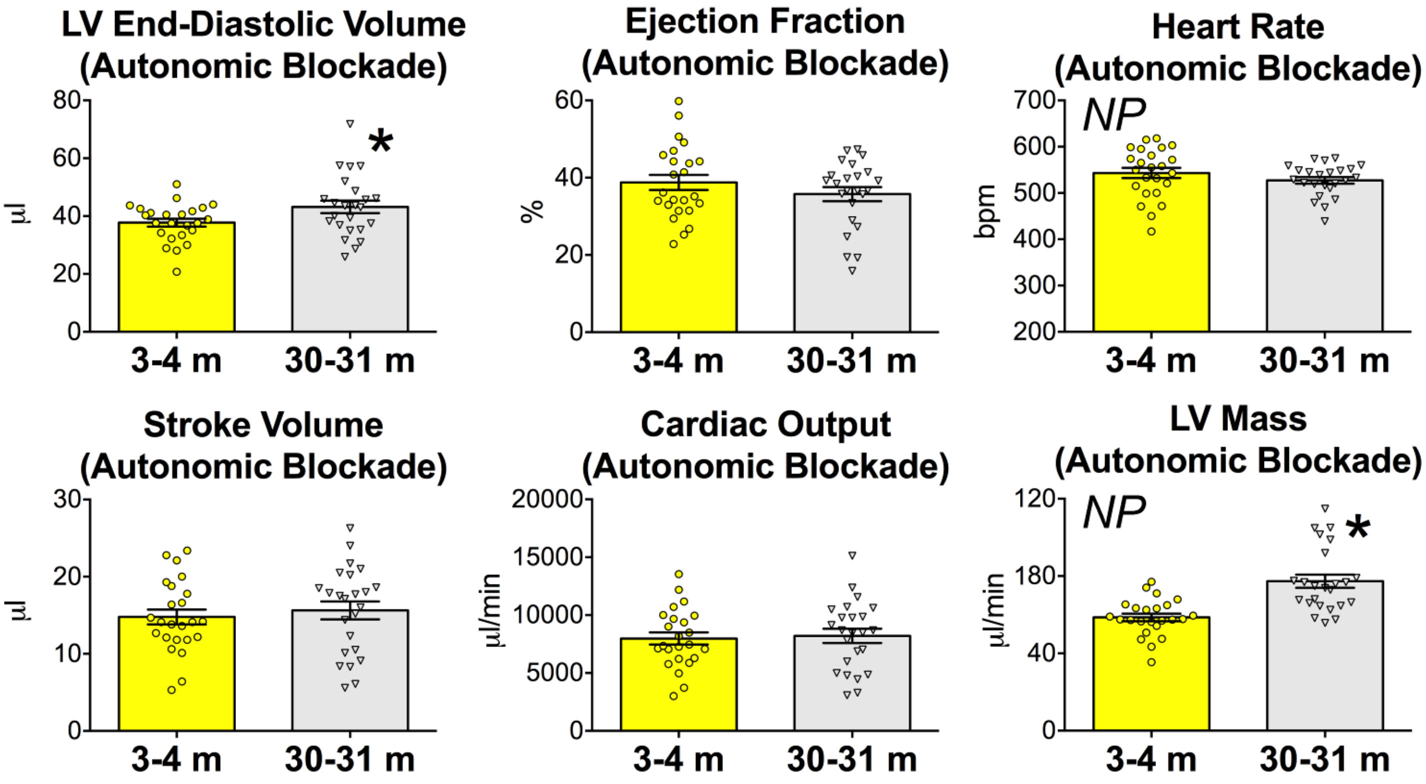

Supplementary Figure 4. Blockade of the autonomous nervous system attenuates defects in cardiac function in aged mice. Anatomical and functional parameters evaluated by echocardiography in aging mice following complete block of the autonomic nervous system with atropine (0.5 mg kg^{-1} body weight, i.p.) plus propranolol (1 mg kg^{-1} body weight, i.p.). Data for male mice at 3-4 months (3-4 m, n = 24) and 30-31 months (30-31 m, n = 24) are shown as mean ± s.e.m. and scatter plots. *P<0.05 versus 3-4 m (Student's t-test and Mann-Whitney rank sum test); NP: non-parametric analysis.

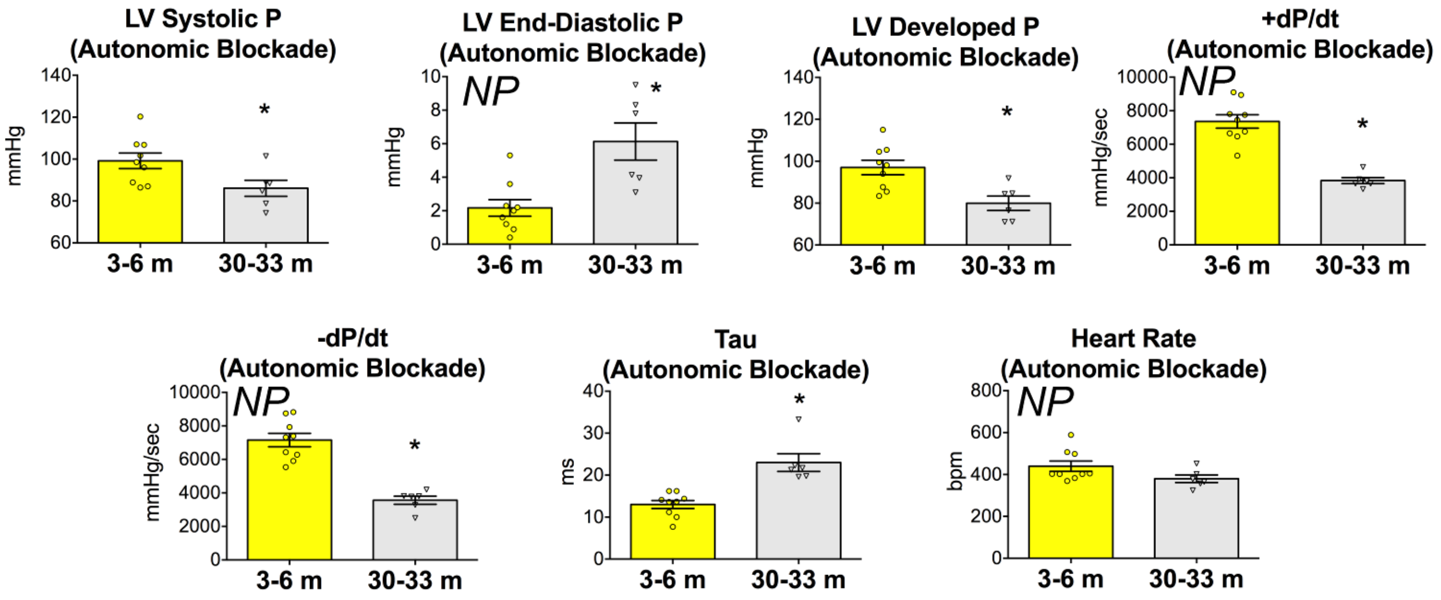

Supplementary Figure 5. Blockade of the autonomous nervous system does not abrogate diastolic and systolic defects in aged mice. Functional parameters evaluated by LV hemodynamics in aging mice following complete block of the autonomic nervous system with atropine(0.5 mg kg^{-1} body weight, i.p.) plus propranolol (1 mg kg^{-1} body weight, i.p.). Data for male mice at 3-4 months (3-6 m, n = 9) and 30-33 months (30-33 m, n = 6) are shown as mean ± s.e.m. and scatter plots. *P<0.05 versus 3-6 m (Student's t-test and Mann-Whitney rank sum test); *NP*: non-parametric analysis.

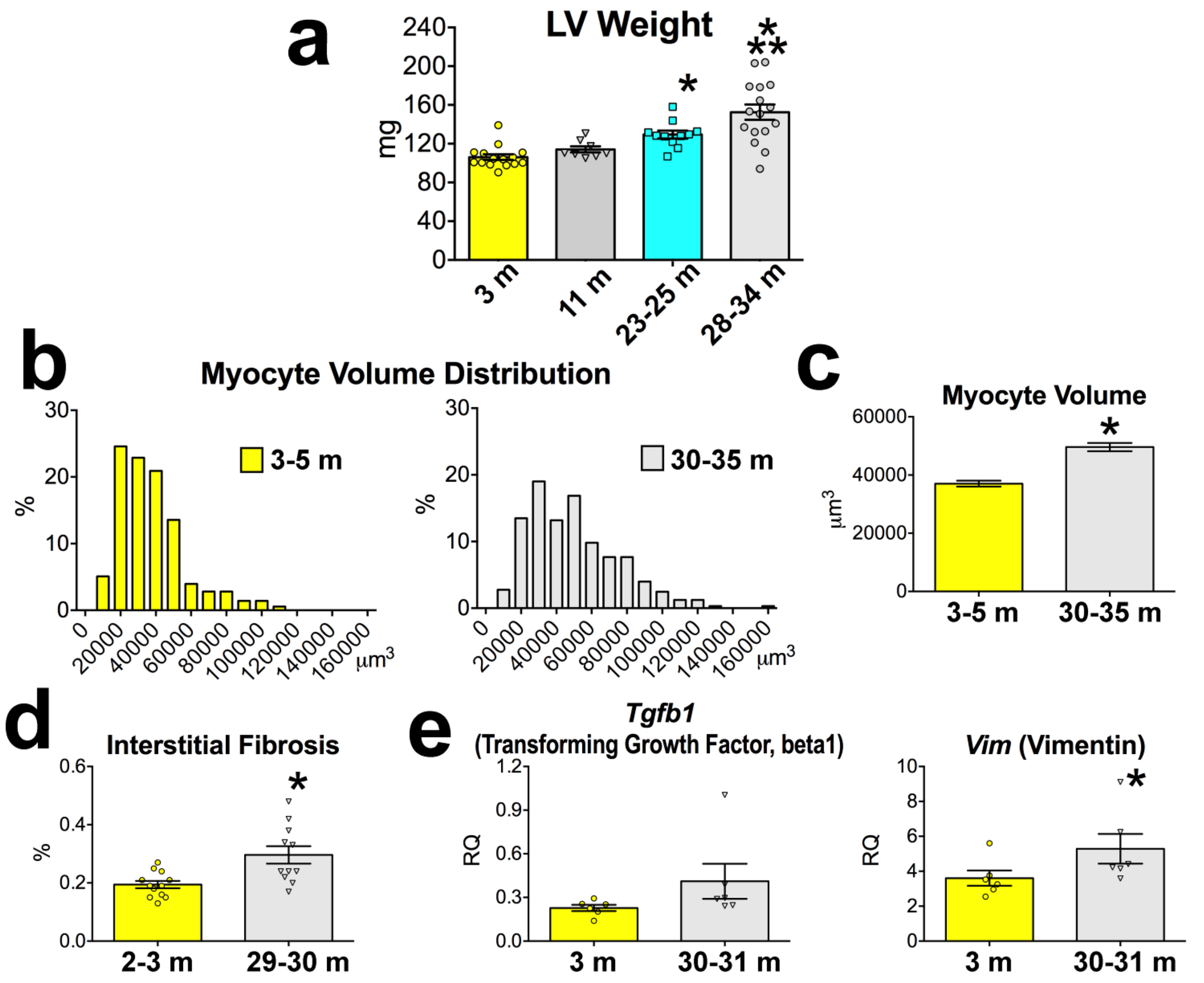

Supplementary Figure 6. Aging and cardiac structure. (**a**) LV weights for male mice at 3 months (3 m, n = 15), 11 months (11 m, n = 8), 23-25 months (23-25 m, n = 11), and 28-34 months (28-34 m, n = 16) are shown as mean ± s.e.m. and scatter plots. *P<0.05 versus 3 m; **P<0.05 versus 11 m; **P<0.05 versus 23-25 m (Kruskal-Wallis one-way ANOVA on ranks with Dunn's *post hoc* test). (**b**) Volume distribution of cardiomyocytes from male mice at 3-5 months (3-5 m, n = 354 cells from 5 hearts) and 30-35 months (30-35 m, n = 326 cells from 5 hearts). (**c**) Data presented in **b** are shown as mean ± s.e.m. and scatter plots. *P<0.001 versus 3-5 m (Mann-Whitney rank sum test). (**d**) Quantitative measurements of interstitial fibrosis in male mice at 2-3 months (2-3 m, n = 12), 29-30 months (29-30 m, n = 11) are shown as mean ± s.e.m. and scatter plots. *P<0.01 versus 2-3 m (Mann-Whitney rank sum test). (**e**) Quantitative data for the transcript levels of transforming growth factor, beta1 (Tgfb1) and vimentin (Vim) in the LV of male mice at 3 months (3 m, n = 6) and 30-31 months (30-31 m, n = 6) are shown as mean ± s.e.m. and scatter plots. *P<0.05 versus 3 m (Mann-Whitney rank sum test).

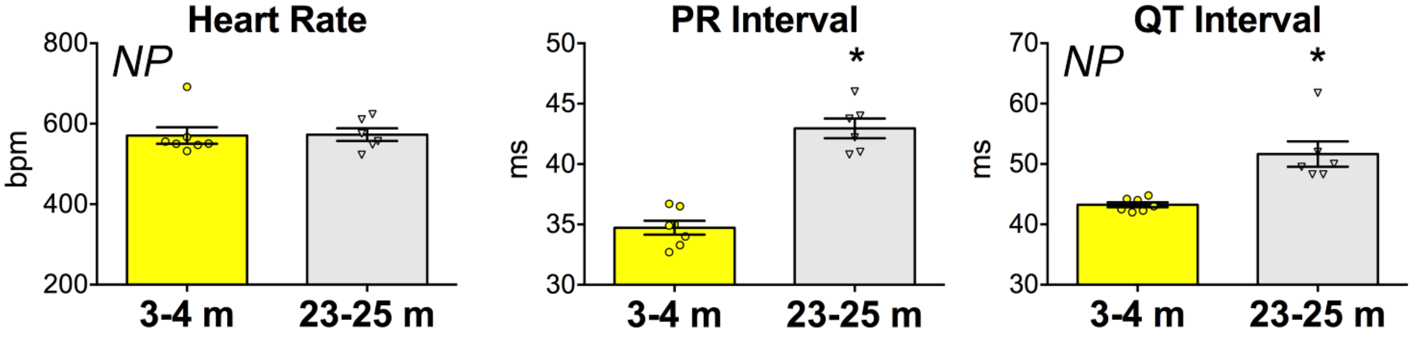

Supplementary Figure 7. Electrical remodeling of the aging heart. Quantitative data for electrocardiographic parameters evaluated by telemetry in conscious male mice 3-4 months (3-4 m, *n* = 7) and 28-34 months (28-34 m, *n* = 6) are shown as mean ± s.e.m. and scatter plots. **P*<0.01 versus 3-4 m (Student's *t*-test and Mann-Whitney rank sum test); *NP*: non-parametric analysis.

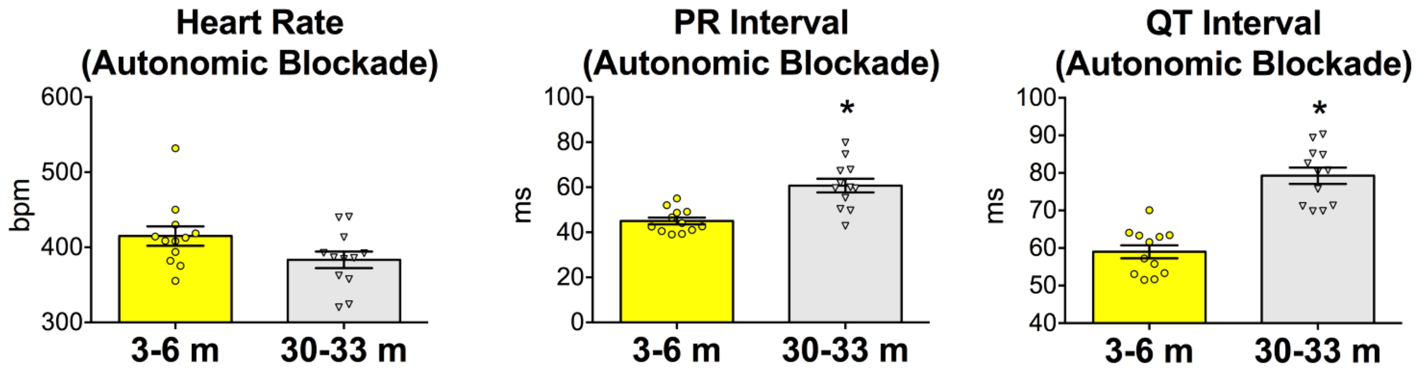

Supplementary Figure 8. Blockade of the autonomous nervous system does not abrogate the electrical remodeling of the aging heart. Quantitative data for electrocardiographic parameters in anesthetized male mice 3-6 months (3-6 m, *n* = 12) and 30-33 months (30-33 m, *n* = 12) following complete block of the autonomic nervous system with atropine (0.5 mg kg^{-1} body weight, i.p.) plus propranolol (1 mg kg^{-1} body weight, i.p.). Data are as mean ± s.e.m. and scatter plots. *P<0.05 versus 3-6 m (Student's *t*-test).

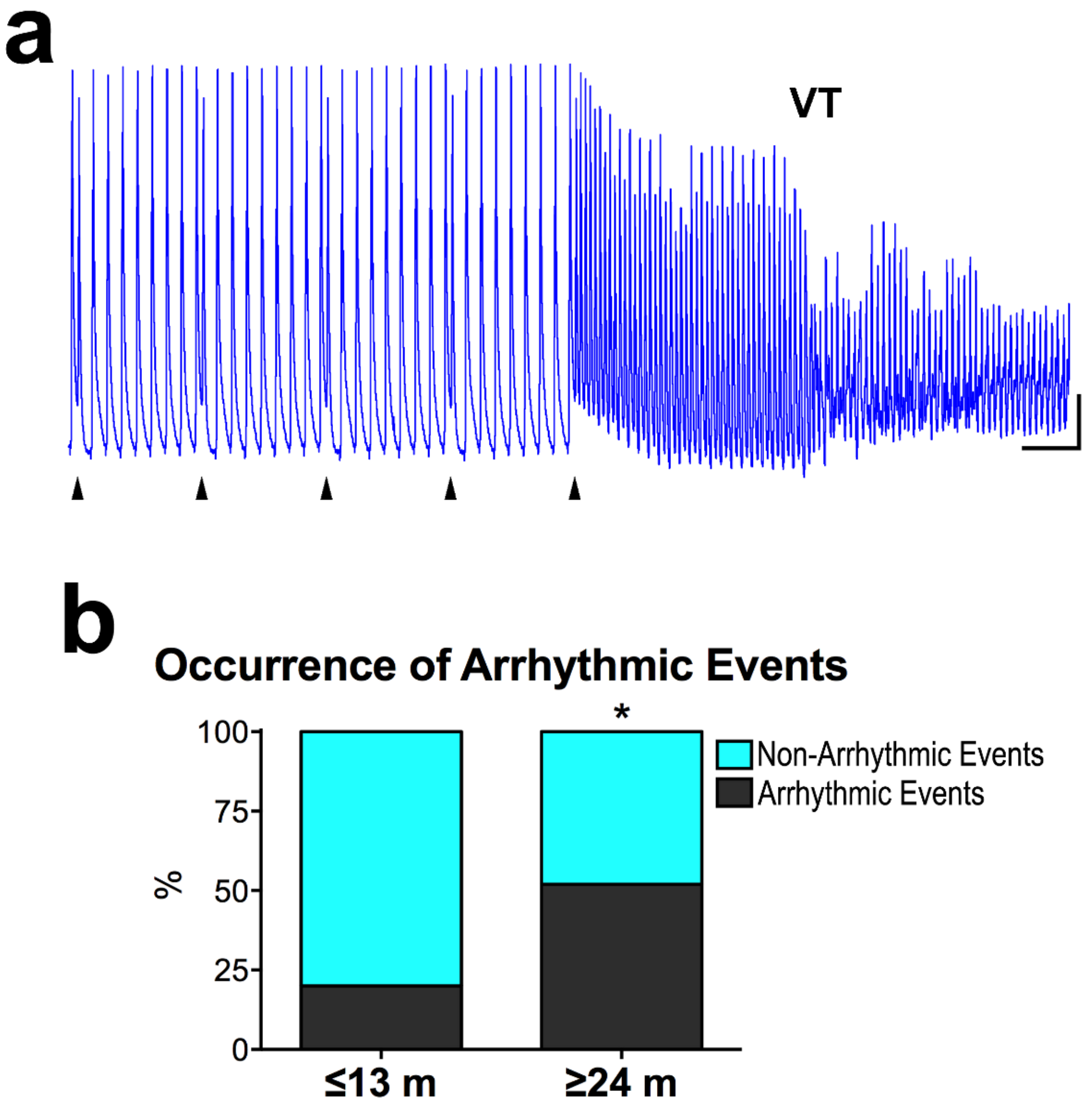

Supplementary Figure 9. Aging is associated by electrical disturbances. (**a**) Monophasic action potentials (MAPs) during a protocol of programmed electrical stimulation (PES) in an old mouse. Reduced time interval for the stimulated premature beats (arrowheads) induces arrhythmia. VT: ventricular tachycardia. Scale bars: 500 ms, 2 mV. (**b**) Quantitative data for hearts obtained from male mice at ≤13 months (≤13 m, n = 25) and ≥24 months (≥24 m, n = 27) are expressed as percentage of organs displaying arrhythmic events following PES. *P<0.05 versus ≤13 m (Fisher exact test).

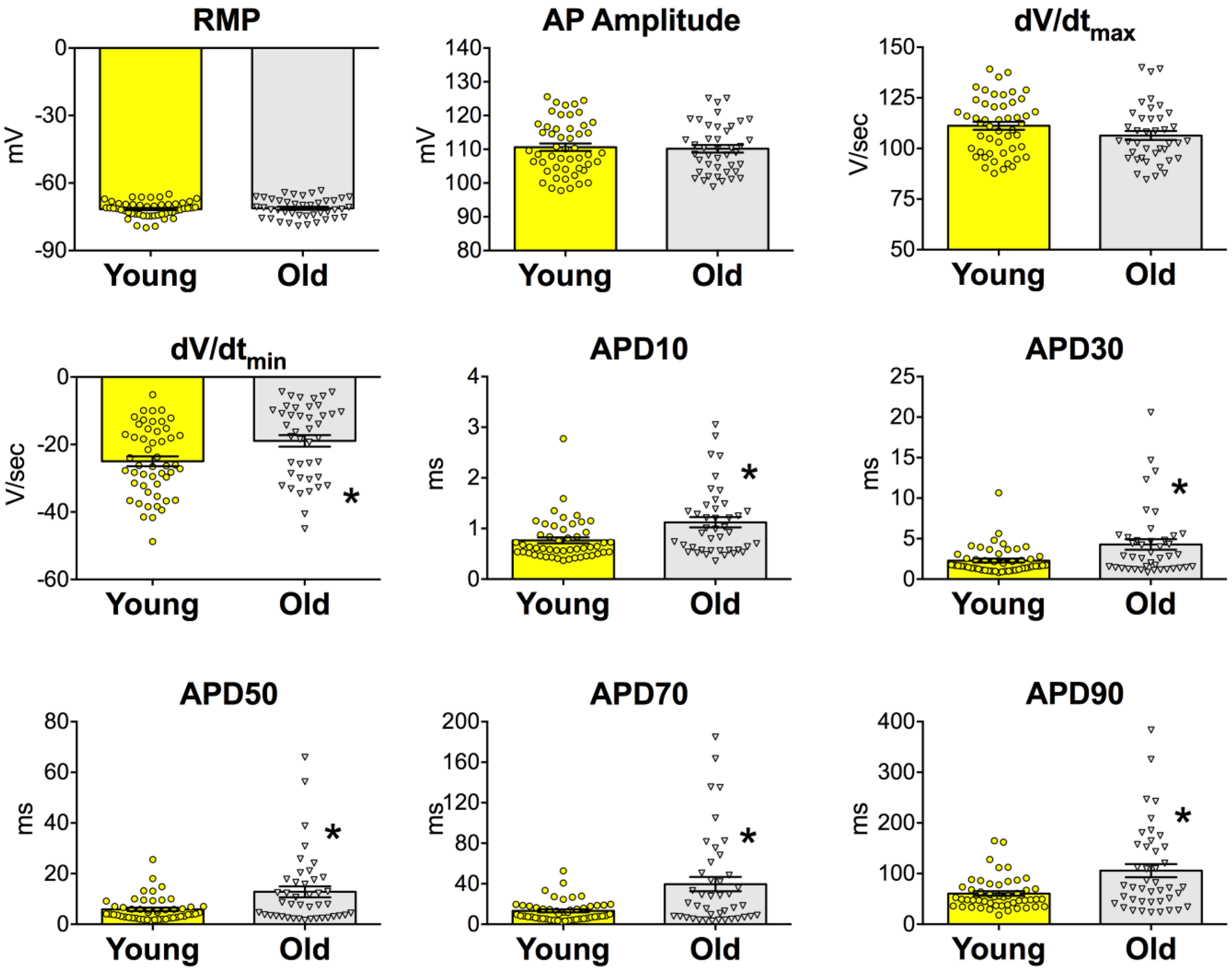

Supplementary Figure 10. Old myocytes present protracted repolarization of the action potential. (**a**) Quantitative data reported in Fig. 2g are shown as mean ± s.e.m and scattered plots. APs were recorded in myocytes isolated from male mice at 3 months (Young, *n* = 50 cells from 18 hearts) and 26-30 months (Old, *n* = 42 cells from 23 hearts) RMP: resting membrane potential. *P<0.05 versus young (Mann-Whitney rank sum test).

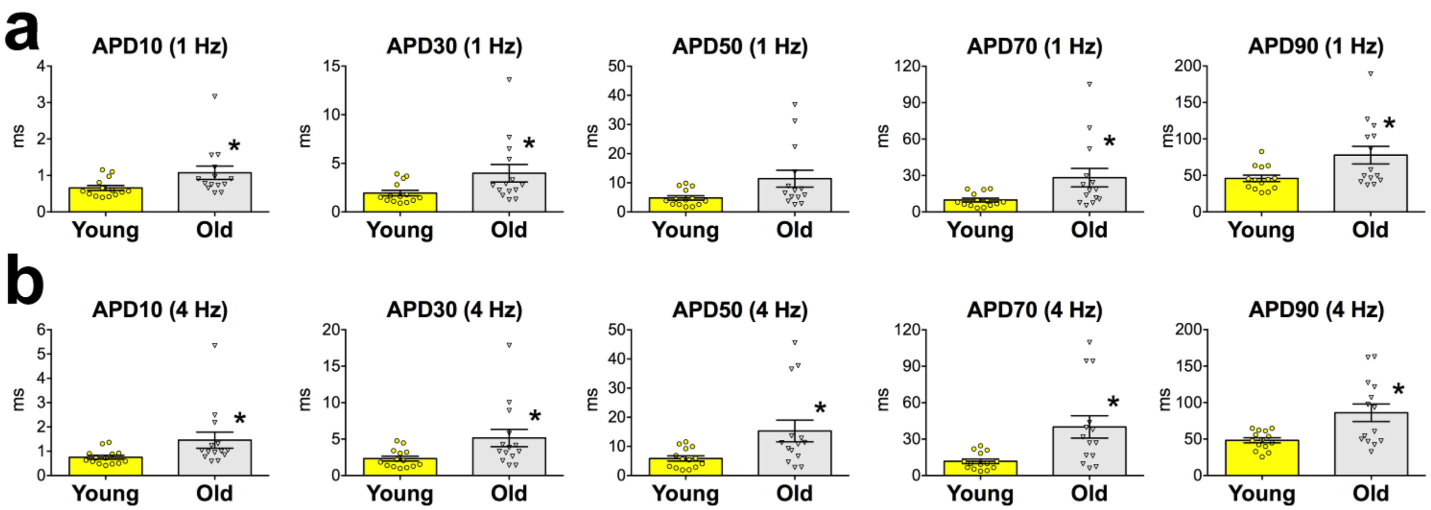

Supplementary Figure 11. Old myocytes present protracted repolarization of the action potential. (**a**,**b**) Action potential repolarization time of myocytes isolated from male mice at 3 months (Young, *n* = 14 cells from 7 hearts) and 30-35 months (Old, *n* = 14 cells from 8 hearts) obtained at 1 Hz (**a**) and 4 Hz (**b**) pacing rate. Data are shown as mean ± s.e.m and scattered plots. *P<0.05 versus Young (Mann-Whitney rank sum test).

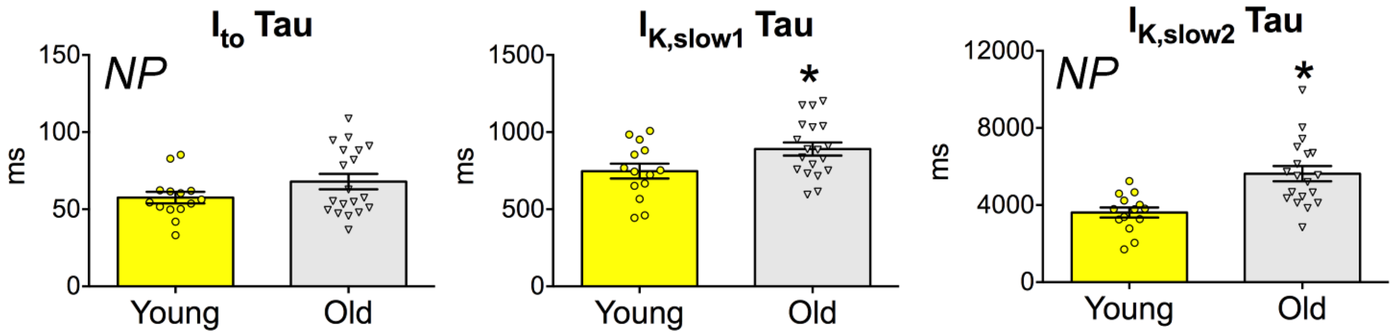

Supplementary Figure 12. Time constant of Kv current components. Expanded parameters of voltage-clamp results reported in Figure 3b. Quantitative data for the time constants of Kv current components in myocytes from mice at 3-4 months (Young, *n* = 14 cells from 5 hearts) and 31-32 months (Old, *n* = 19 cells from 4 hearts) are shown as mean ± s.e.m. **P*<0.05 versus Young (Student's *t*-test and Mann-Whitney rank sum test); *NP*: non-parametric analysis.

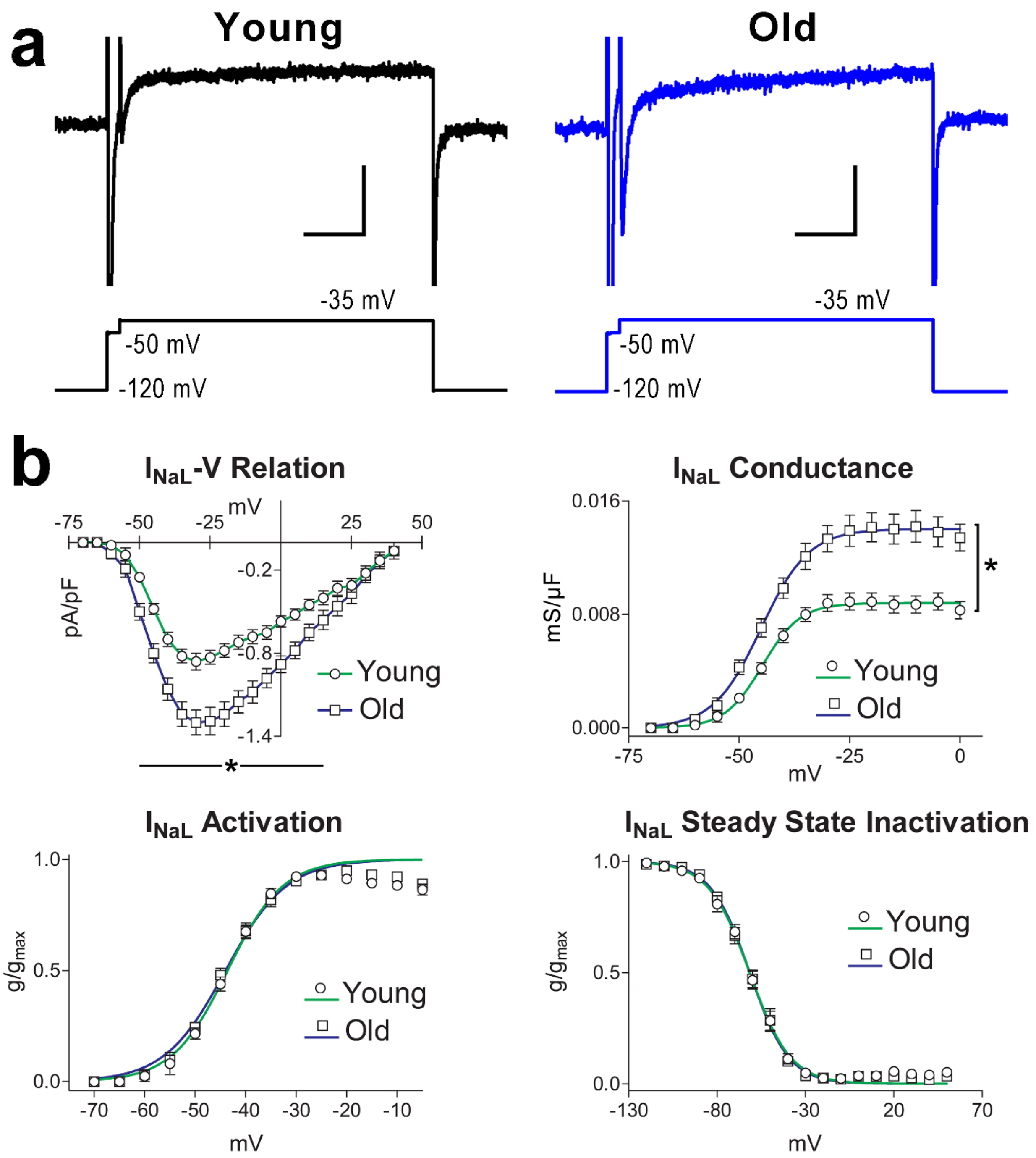

Supplementary Figure 13. The late Na^+ current I_{NaL} is enhanced in old myocytes. (**a**) Whole-cell voltage-gated Na^+ currents in a young (black traces) and an old (blue traces) LV myocyte. Scale bars: 100 ms, 2 pA/pF. The voltage-command protocol is shown in the lower traces. (**b**) Quantitative data for I_{NaL} properties obtained in myocytes from male mice at 3 months (Young, *n* = 16-27 cells from 5 hearts) and 30-31 months (Old, *n* = 9-18 cells from 5 hearts) shown as mean ± s.e.m. **P*<0.05 versus Young in I_{NaL}-V Relation (Student's *t*-test and Mann-Whitney rank sum test). Conductance, activation, and inactivation plots were fitted with Boltzmann functions (Young, solid green line; Old, solid blue line). **P*<0.0001 between fittings (Student's *t*-test). Parameters are reported in Supplementary Table 3.

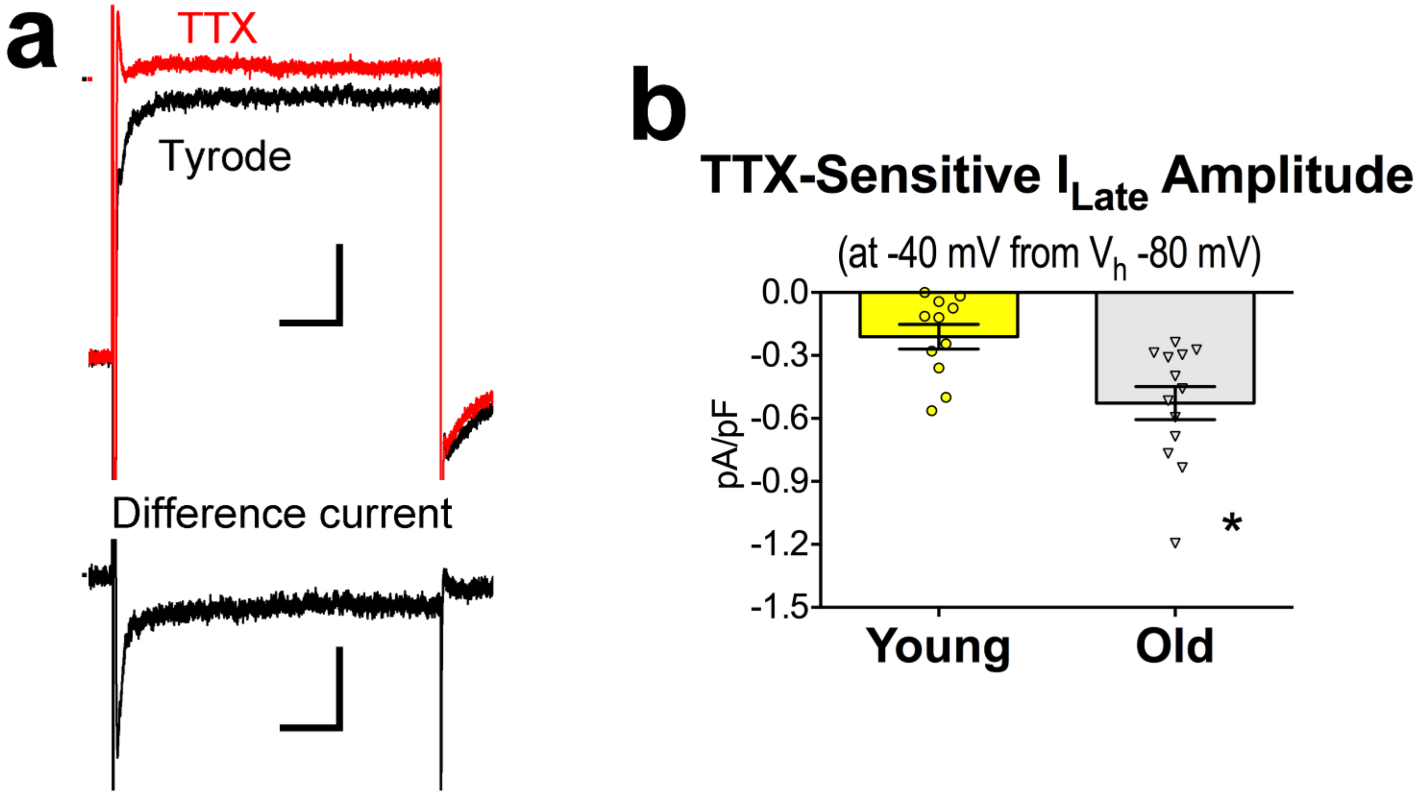

Supplementary Figure 14. A slowly inactivating TTX-sensitive current is enhanced in old myocytes. (**a**) Whole-cell voltage-gated currents recorded in voltage-clamp in an old myocyte with a depolarizing step from -80 mV to -40 mV before (Tyrode, black traces) and after exposure to 10 µM tetrodotoxin (TTX, red traces). Difference current is reported in the lower panel. Scale bars: 100 ms, 200 pA. (**b**) Quantitative data of the time dependent component of the TTX-sensitive difference current obtained in myocytes from male mice at 3-6 months (Young, *n* = 11 cells from 9 hearts) and 31-35 months (Old, *n* = 13 cells from 7 hearts) are shown as mean ± s.e.m. and scatter plots. **P*<0.01 versus Young (Mann-Whitney rank sum test). With the protocol employed, peak I_{Na} was reduced following TTX exposure. Due to specific amplifier gain settings, I_{Na} was saturated in Tyrode solution, but its amplitude was not saturated and comparable between the two cell populations in TTX (74 ± 9 pA/pF in young and 64 ± 9 pA/pF in old myocytes).

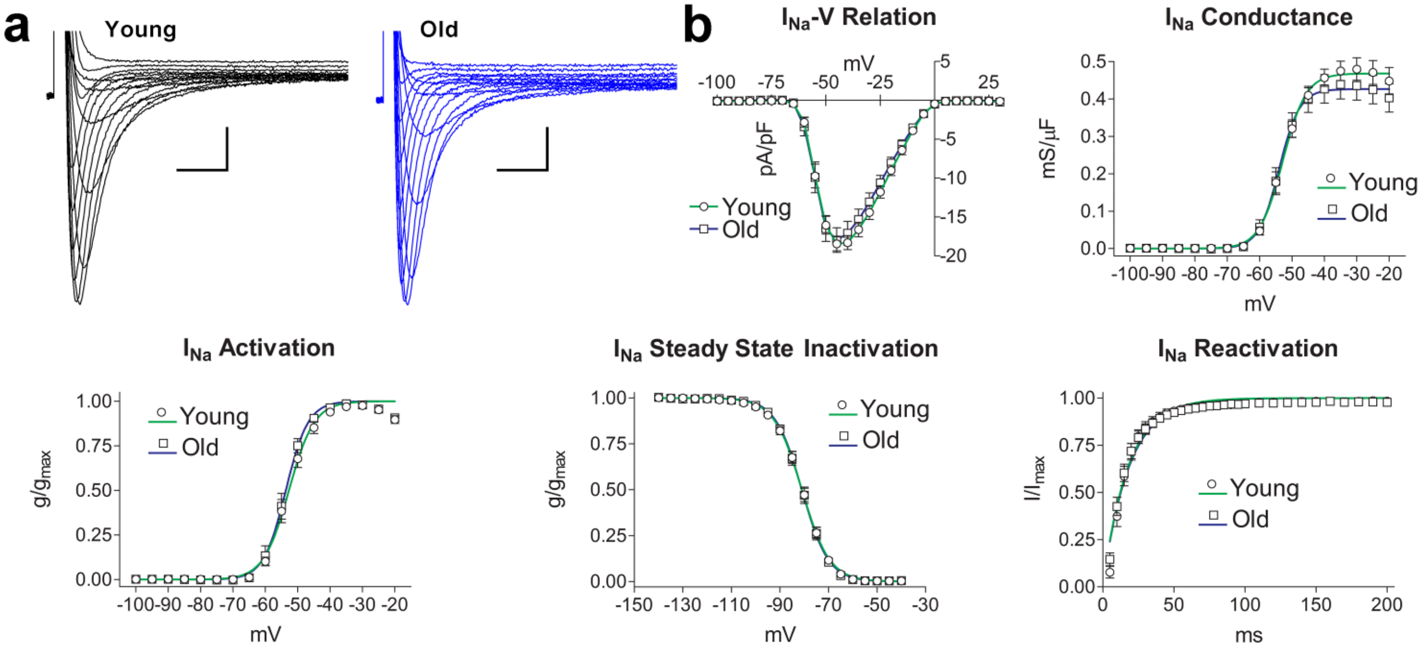

Supplementary Figure 15. The fast Na^+ current I_{Na} is not affected by aging. (**a**) Whole-cell voltage-gated Na^+ currents recorded in voltage-clamp in a young (black traces) and an old (blue traces) LV myocyte. Scale bars: 6 ms, 3 pA/pF. (**b**) I-V relations, voltage-dependency of conductance, steady state activation, and steady state inactivation, time dependency of reactivation for I_{Na} in myocytes from male mice at 3 months (Young, *n* = 17-26 cells from 4 hearts) and 29-31 months (Old, *n* = 16-19 cells from 4 hearts) are shown as mean ± s.e.m. Conductance, activation, and inactivation plots were fitted with Boltzmann functions, and reactivation plots with an exponential function (Young, solid green line; Old, solid blue line). Parameters are reported in Supplementary Table 3.

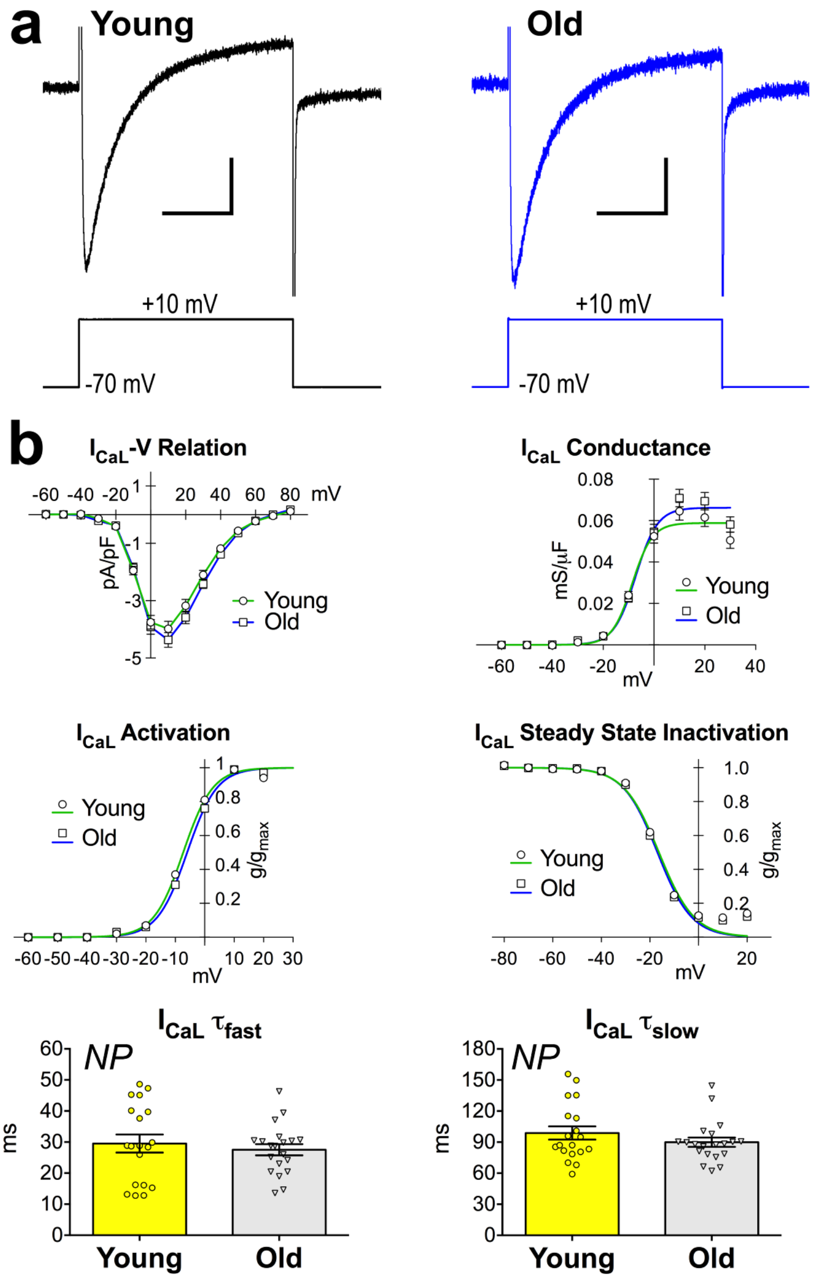

Supplementary Figure 16. The L-type Ca^{2+} current I_{CaL} is not affected by aging. (**a**) Whole-cell voltage-gated Ca^{2+} currents recorded in voltage-clamp in a young (black traces) and an old (blue traces) LV myocyte. Scale bars: 100 ms, 1 pA/pF. (**b**) L-type Ca^{2+} current properties obtained in myocytes from male mice at 3 months (Young, n = 19 cells from 5 hearts) and 27 months (Old, n = 17-20 cells from 5 hearts). I-V relations, voltage-dependency of conductance, steady state activation, and steady state inactivation for I_{CaL} are shown as mean ± s.e.m. Fast and slow time constants (τ) for I_{CaL} were measured at 0 mV and are shown as mean ± s.e.m. and scatter plots. Conductance, activation, and inactivation plots were fitted with Boltzmann functions (Young, solid green line; Old, solid blue line). Parameters are reported in Supplementary Table 3. *NP*: non-parametric analysis.

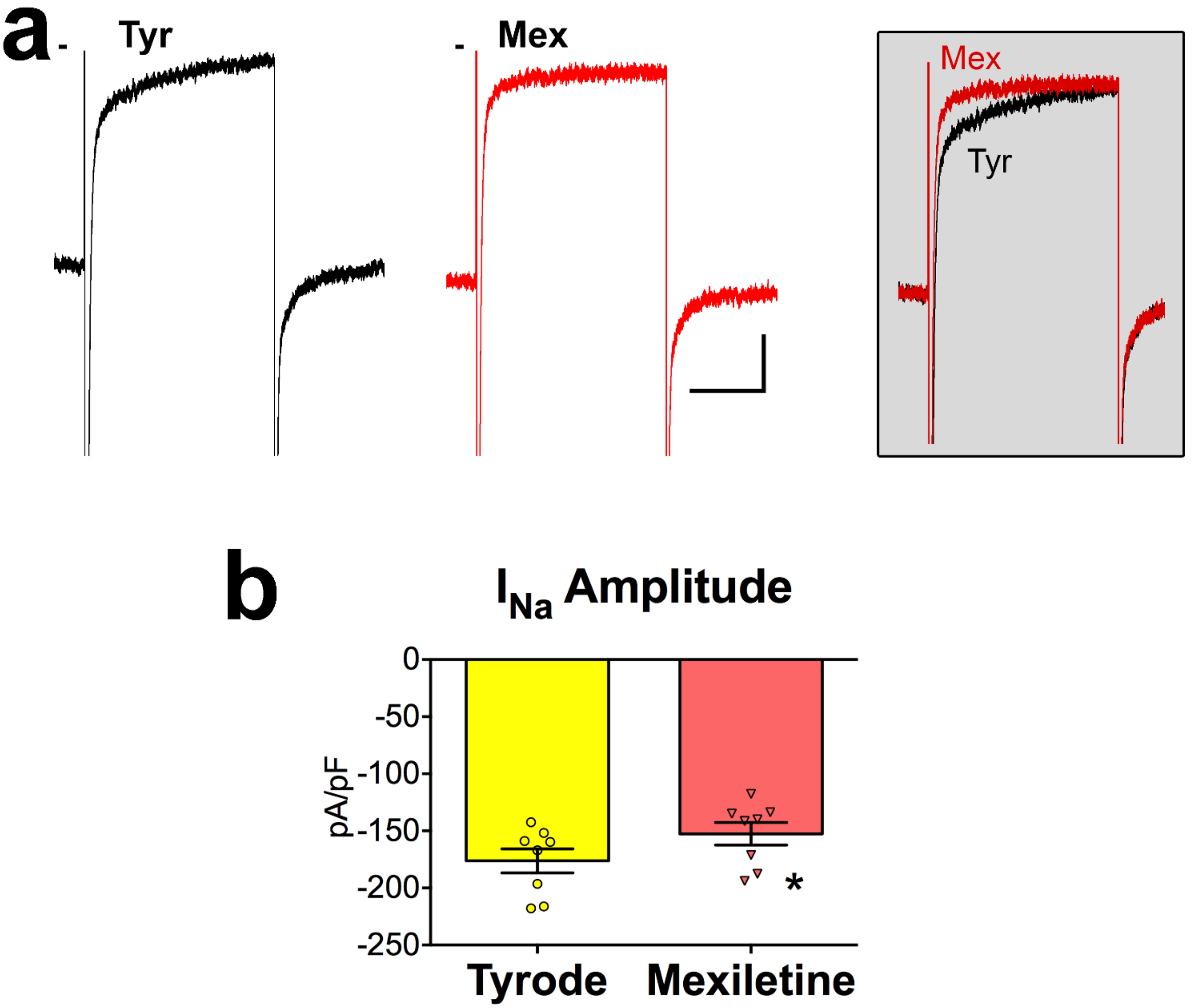

Supplementary Figure 17. Mexiletine inhibits I_{NaL}. (**a**) Current traces in voltage-clamp mode showing the effects of mexiletine on I_{NaL} in a myocytes obtained from a male mouse at 27 month of age. I_{NaL} was elicited by a depolarizing step from V_h -90 mV to -30 mV. Traces recorded before (black trace) and in the presence of 30 µM mexiletine (Mex, red trace) are superimposed in the inset. Scale bars: 200 ms, 40 pA. (**b**) Quantitative data for peak I_{Na} density elicited by depolarizing pulses to -40 mV from V_h -70 in myocytes from male mice at 5-6 months (n = 6 cells from 3 hearts) before (Tyrode) and after exposure to the 10 µM mexiletine are shown as mean ± s.e.m. and scatter plots. *P<0.01 versus Tyrode (Wilcoxon signed rank test).

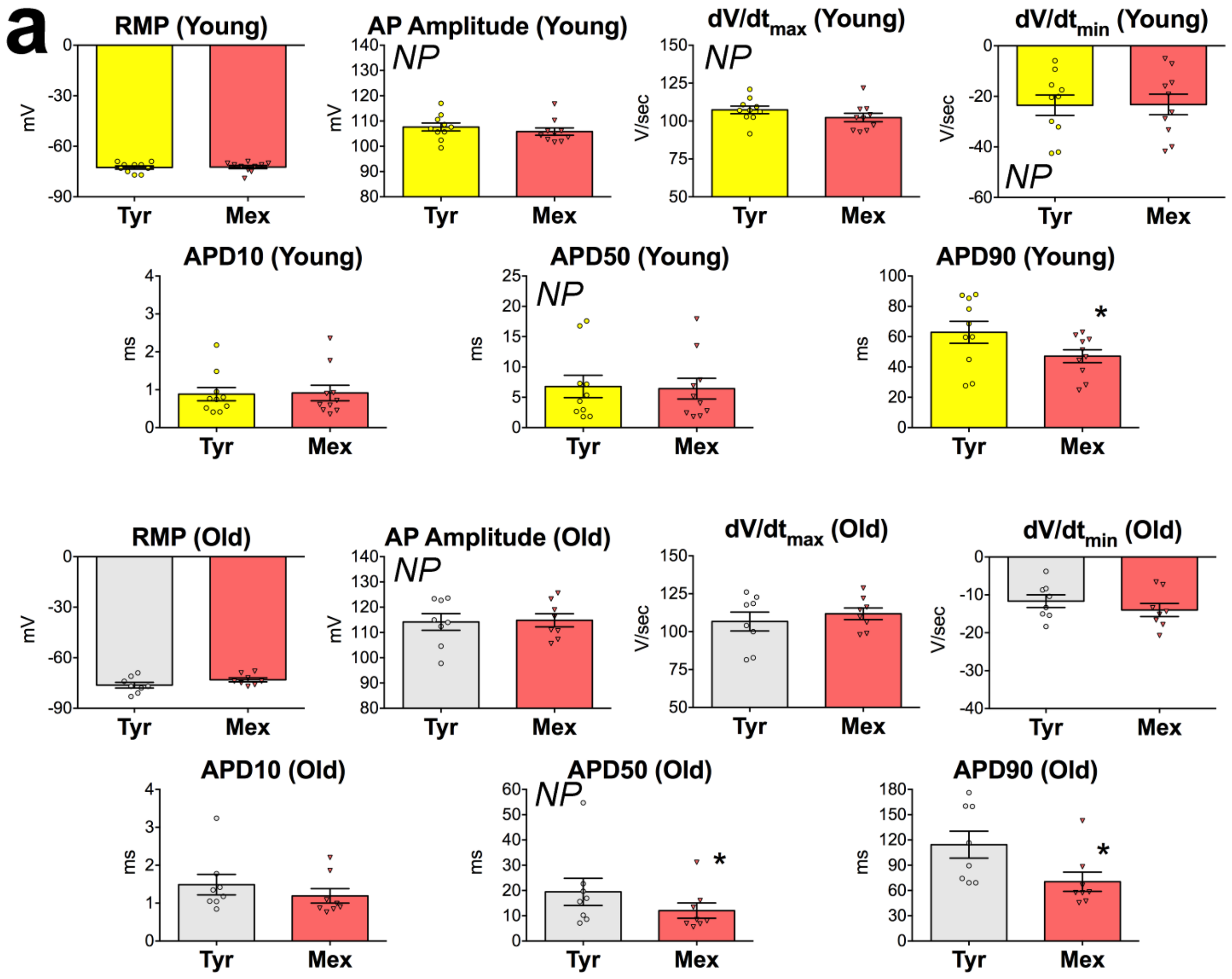

Supplementary Figure 18a. Inhibition of I_{NaL} shortens the duration of the action potential. (**a**) Expanded AP parameters of data shown in Figure 4b. Data obtained in myocytes from male mice at 3 months (Young, *n* = 10 cells from 5 hearts) and 27-30 months (Old, *n* = 8 cells from 5 hearts) before (Tyr) and after exposure to 10 µM mexiletine (Mex) are shown as mean ± s.e.m. and scatter plots. Tyr: Tyrode solution. *P<0.05 versus Tyr (paired *t*-test and Wilcoxon signed rank test); NP: non-parametric analysis.

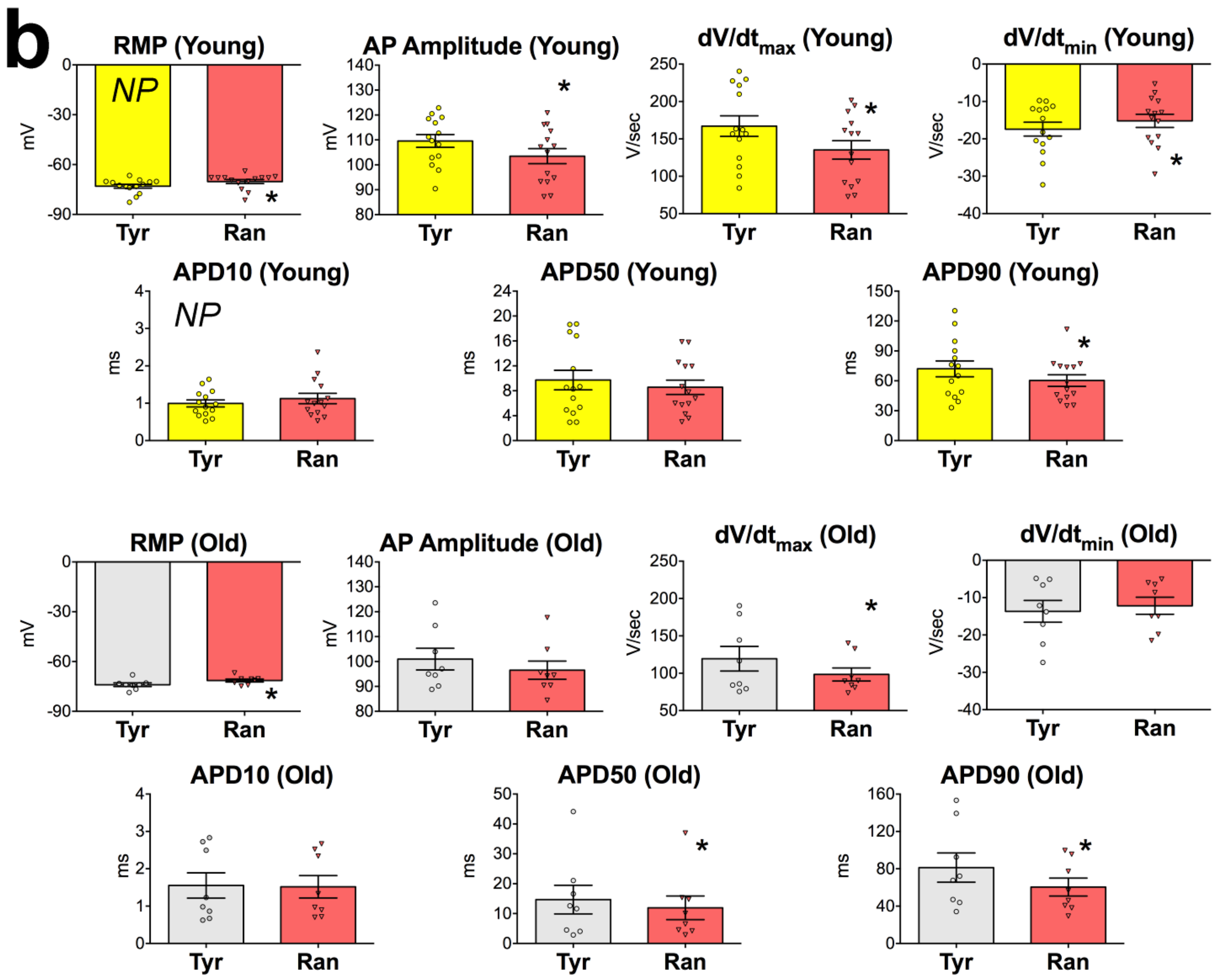

Supplementary Figure 18b. Inhibition of I_{NaL} shortens the duration of the action potential. (**b**) Expanded AP parameters of data shown in Figure 4d. Data obtained in mice at 3-6 months (Young, *n* = 14 cells from 4 hearts) and 27-33 months (Old, *n* = 8 cells from 5 hearts) before (Tyr) and after exposure to 10 µM ranolazine (Ran) are shown as mean ± s.e.m. and scatter plots. *P<0.05 versus Tyr (paired *t*-test and Wilcoxon signed rank test); NP: non-parametric analysis.

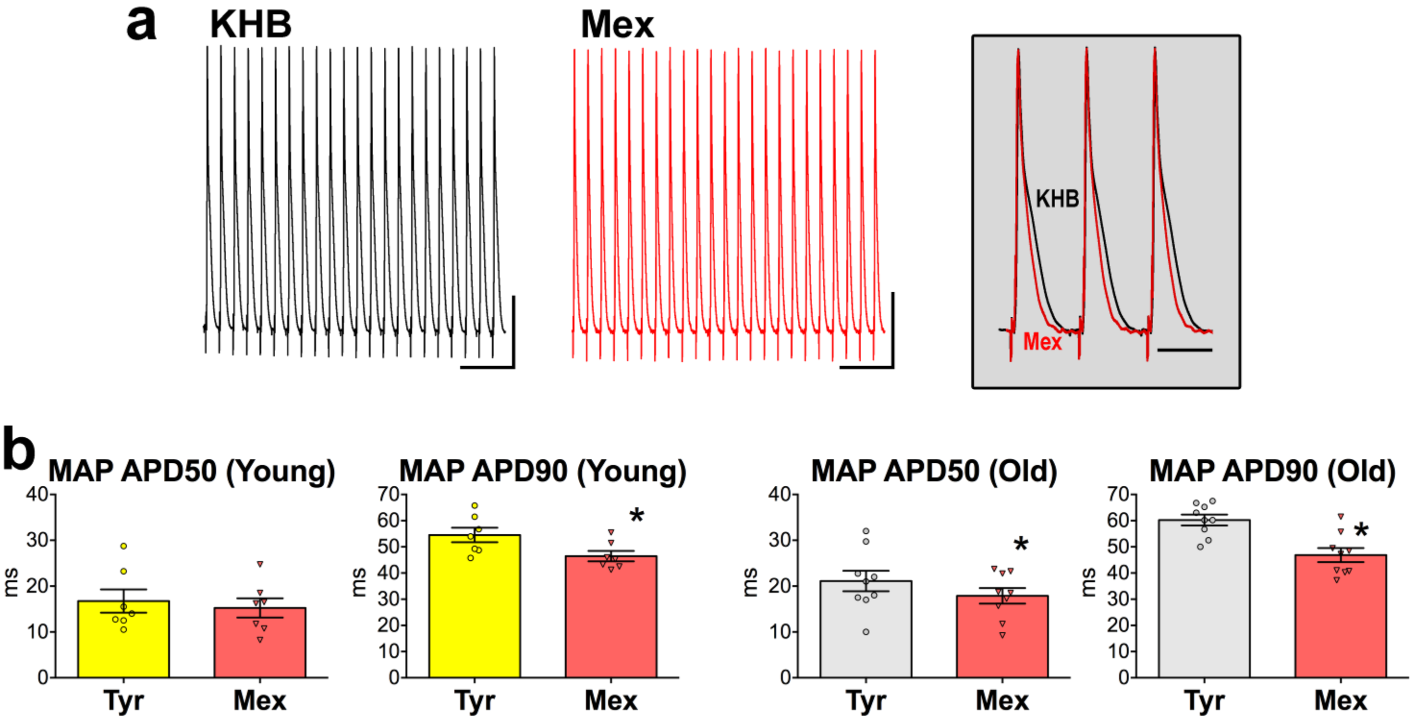

Supplementary Figure 19. Inhibition of I_{NaL} shortens local epicardial monophasic APs. (**a**) Monophasic AP recorded before (KHB, black traces) and after perfusion of 10 µM mexiletine (Mex, red traces) in an old mouse heart. Scale bars: 500 ms, 2 mV. Traces are superimposed in the inset. Scale bar: 100 ms. (**b**) Quantitative data of repolarization times for hearts from female mice at 5 months (Young, *n* = 7) and male mice at 24-30 months (*n* = 9) before (KHB, Krebs–Henseleit buffer) and after exposure to the I_{NaL} inhibitor mexiletine (Mex) are shown as median and IQR. *P<0.05 versus KHB (paired *t*-test).

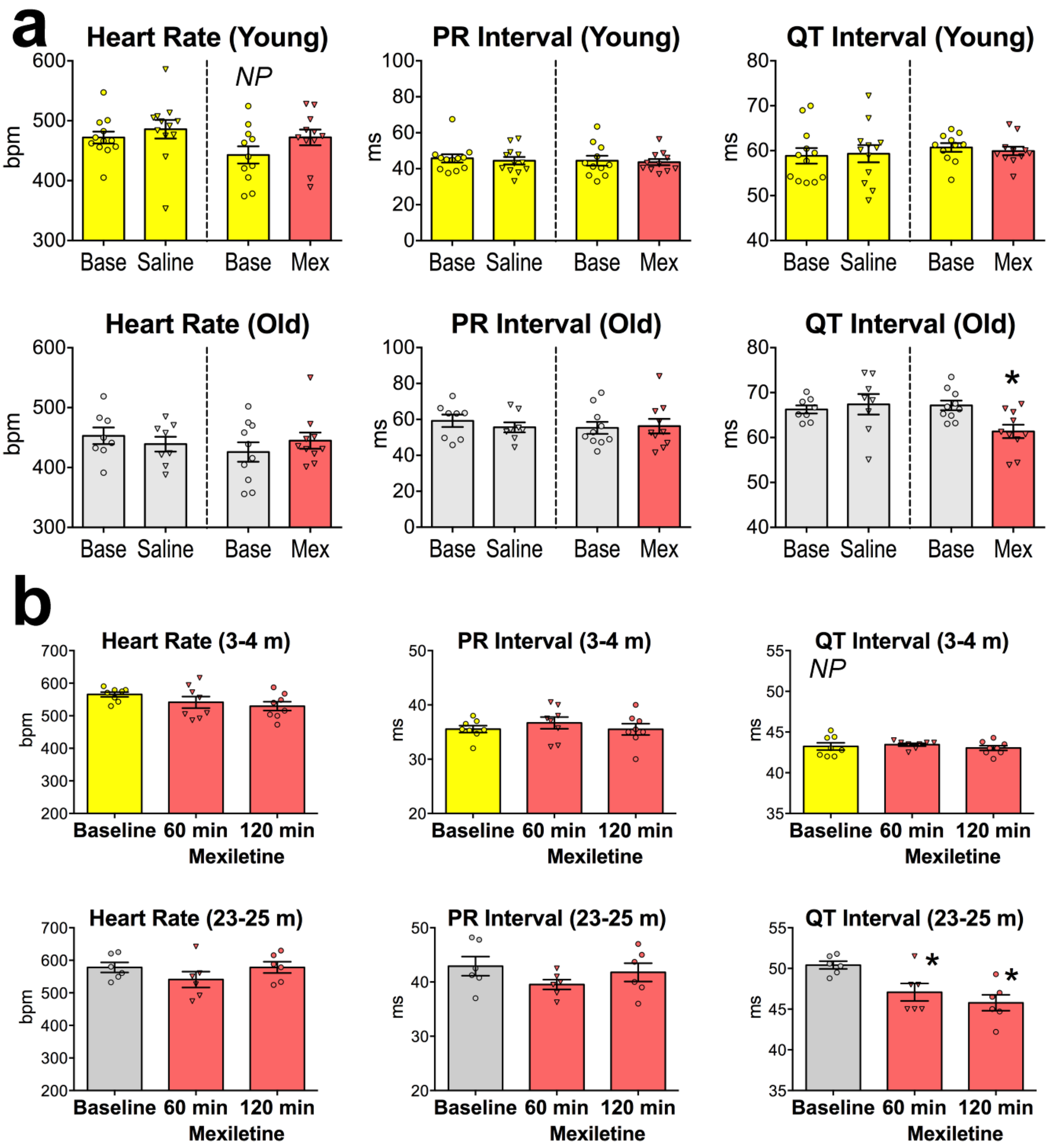

Supplementary Figure 20. Inhibition of I_{NaL} shortens the electrical recovery of the old heart. (**a**) Quantitative data for electrocardiographic parameters obtained in male mice at 3 months (Young) and at 30 months (Old) at baseline (Base) and 1 hour after treatment with saline (Young, *n* = 12; Old *n* = 8) or mexiletine (5 mg kg^{-1} body weight, i.p.) (Young, *n* = 11; Old *n* = 10). Data are shown as mean ± s.e.m and scatter plots. **P*<0.05 versus Base (paired *t*-test and Wilcoxon signed rank test); *NP*: non-parametric analysis. (**b**) Quantitative data for electrocardiographic parameters obtained in conscious male mice at 3-4 months (3-4 m, *n* = 8) and 23-25 months (23-25 m, *n* = 6) treated with mexiletine (5 mg kg^{-1} body weight) and followed for a two-hour period. Data are shown as mean ± s.e.m and scatter plots. **P*<0.05 versus baseline (before administration of the inhibitor, one-way ANOVA with Bonferroni's *post hoc* test); *NP*: non-parametric analysis.

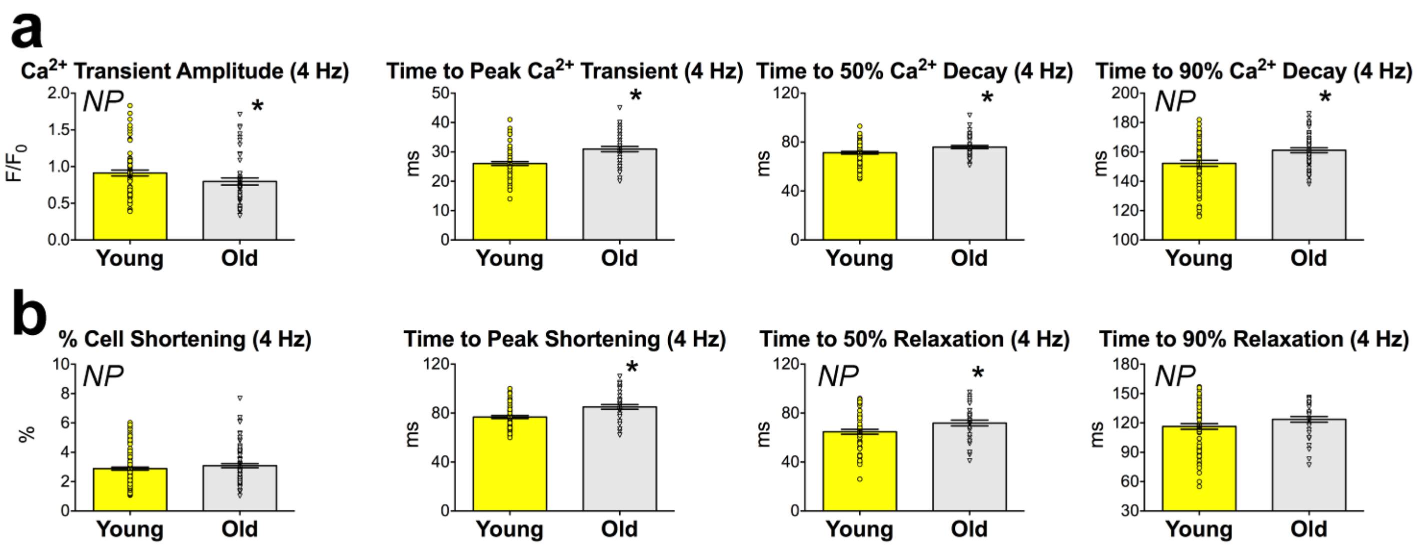

Supplementary Figure 21. Aging delays the kinetics of Ca^{2+} transients and cell shortening in LV myocytes. (**a**) Ca^{2+} transient properties assessed at 4 Hz pacing rate in myocytes from male mice at 3-6 months (Young, n = 69 cells from 4 hearts) and 30-35 months (Old, n = 49 cells from 4 hearts) are shown as mean ± s.e.m and scatter plots. *P<0.05 versus Young (Student's t-test and Mann-Whitney rank sum test); *NP*: non-parametric analysis. (**b**) Quantitative data for cell shortening properties obtained in myocytes from mice at 3 months (Young, n = 69 cells from 7 hearts) and 29-30 months (Old, n = 41 cells from 6 hearts) stimulated at 4 Hz pacing rate; data are shown as mean ± s.e.m and scatter plots. *P<0.05 versus Young (Student's t-test and Mann-Whitney rank sum test); *NP*: non-parametric analysis.

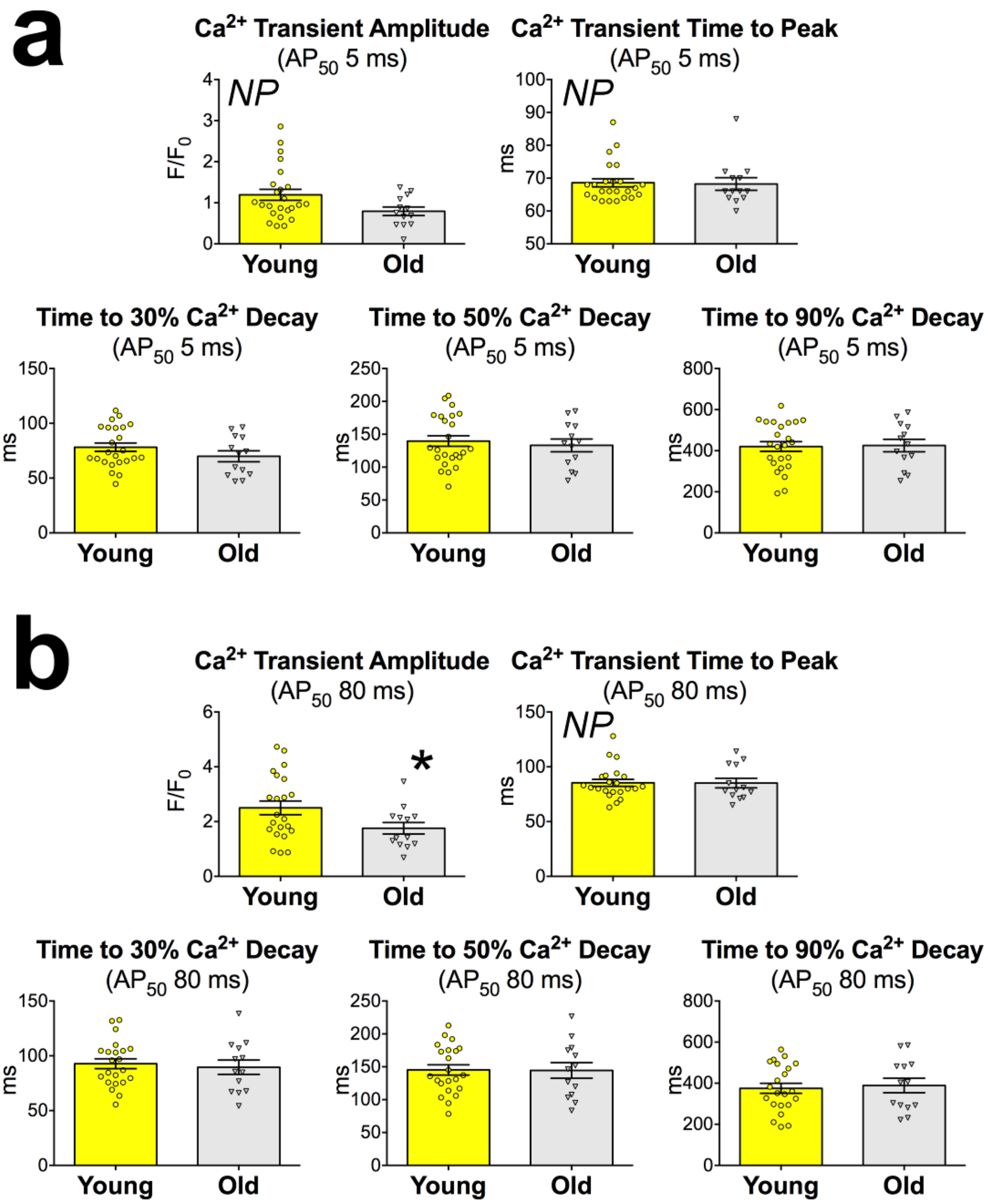

Supplementary Figure 22. I_{NaL} alters the kinetics of contraction of LV myocytes. (**a**,**b**) Quantitative properties of Ca^{2+} transients elicited in young and old myocytes by AP-clamp protocols with short (**a**) and long (**b**) AP shape. Data for myocytes from male mice at 3-5 months (Young) and 28-33 months (Old) are shown as mean ± s.e.m. (**a**) Young, *n* = 24 cells from 5 hearts; Old, *n* = 13 cells from 2 hearts; (**b**) Young, *n* = 22 cells from 5 hearts; Old, *n* = 13 cells from 2 hearts. *P<0.05 versus Young (Student's t-test and Mann-Whitney rank sum test); *NP*: non-parametric analysis. For Ca^{2+} Transient Amplitude in **b**, *P = 0.049.

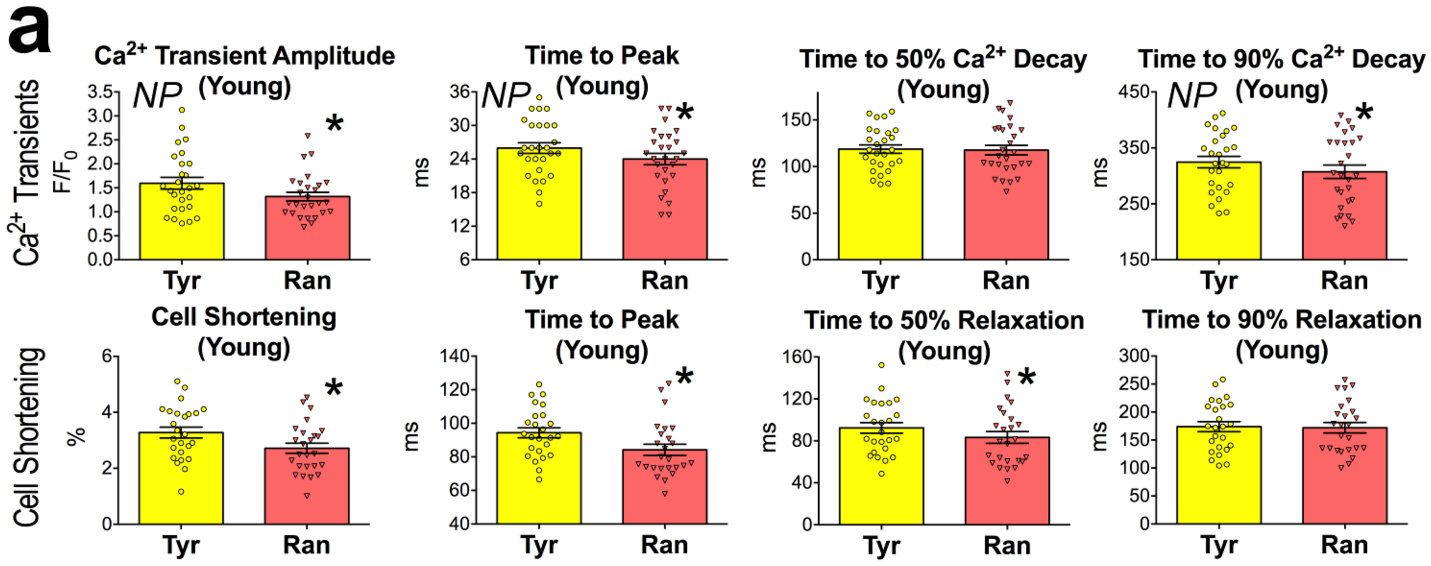

Supplementary Figure 23a. I_{NaL} alters the amplitude and kinetics of Ca^{2+} transients and contraction of LV myocytes. (**a**) Quantitative data for Ca^{2+} transient and cell shortening properties in myocytes from male mice at 3 months, before (Tyr) and after exposure to 10 µM ranolazine (Ran) (Ca^{2+} transients: *n* = 27 cells from 10 hearts; cell shortening *n* = 26 cells from 5 hearts); data are shown as mean ± s.e.m. and scatter plots. *P<0.05 versus Tyr (paired *t*-test and Wilcoxon signed rank test); *NP*: non-parametric analysis.

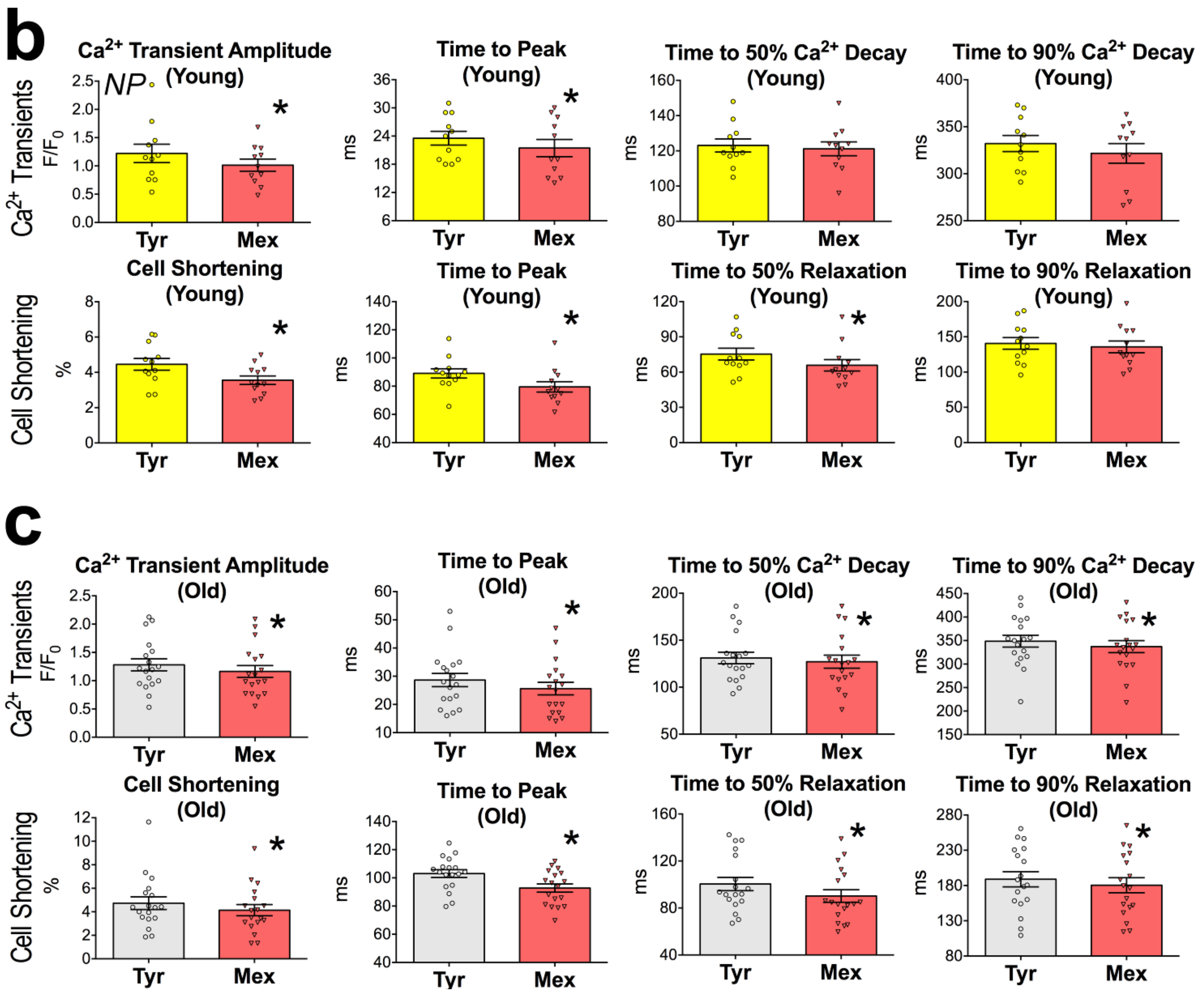

Supplementary Figure 23b and c. I_{NaL} alters the amplitude and kinetics of Ca^{2+} transients and contraction of LV myocytes. (**b**) Quantitative data for Ca^{2+} transient and cell shortening properties in myocytes from male mice at 3 months, before (Tyr) and after exposure to 10 µM mexiletine (Mex) (Ca^{2+} transients: *n* = 11 cells from 2 hearts; cell shortening *n* = 12 cells from 2 hearts); data are shown as mean ± s.e.m. and scatter plots. *P<0.05 versus Tyr (paired *t*-test and Wilcoxon signed rank test); *NP*: non-parametric analysis. (**c**) Quantitative data for Ca^{2+} transient and cell shortening properties in myocytes from male mice at 30-31 months, before (Tyr) and after exposure to 10 µM mexiletine (Mex) (Ca^{2+} transients: *n* = 18 cells from 4 hearts; cell shortening *n* = 18 cells from 3 hearts); data are shown as mean ± s.e.m. and scatter plots. *P<0.05 versus Tyr (paired *t*-test).

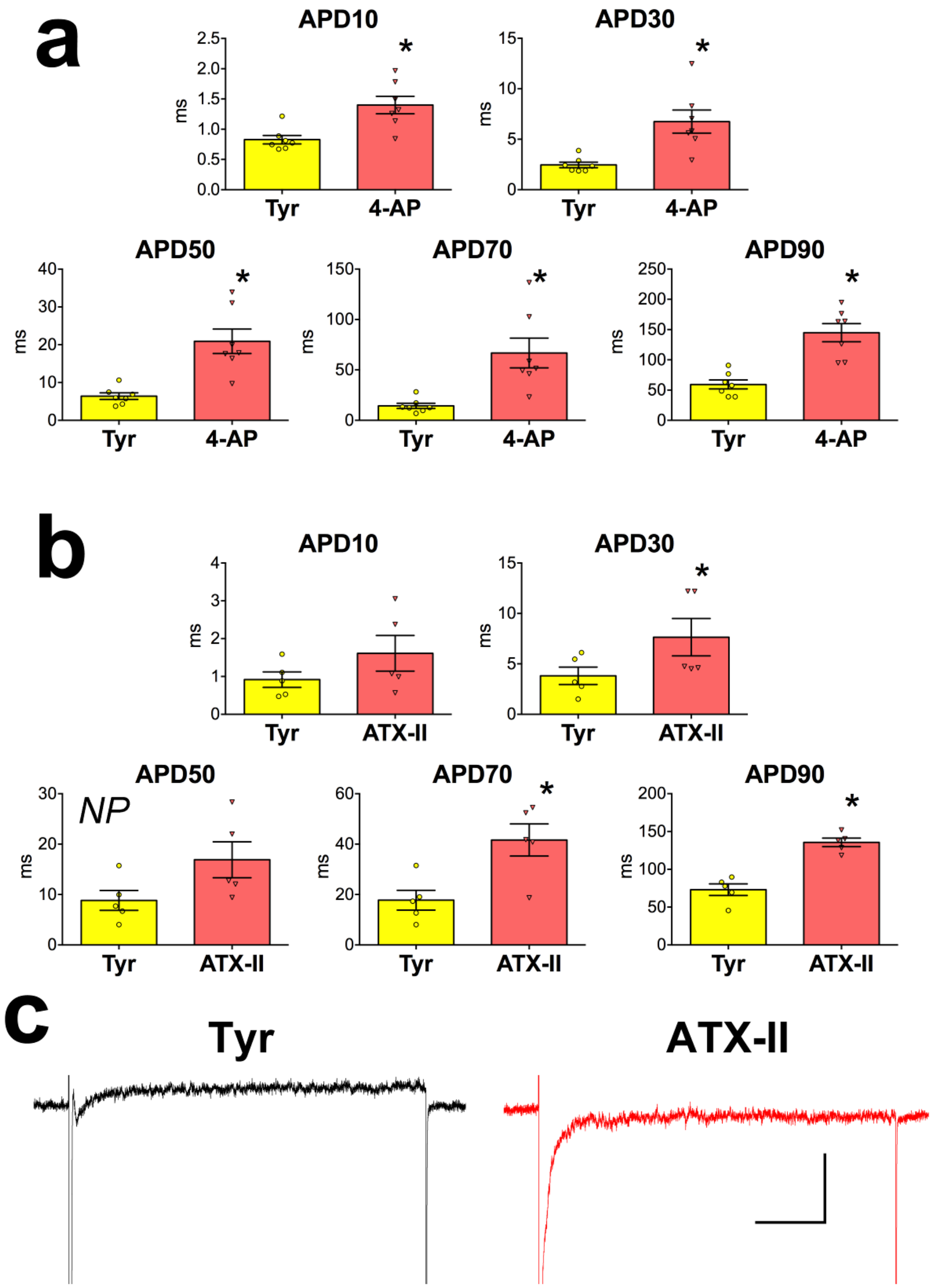

Supplementary Figure 24. Prolongation of the AP with pharmacological interventions. (**a**) Quantitative AP repolarization properties in myocytes from female mice at 3 months, before (Tyr) and after exposure to 0.5 mM 4-aminopyridine (4-AP) (*n* = 7 cells from 3 hearts); data are shown as mean ± s.e.m. and scatter plots. **P*<0.05 versus Tyr (paired *t*-test). (**b**) Quantitative AP repolarization properties in myocytes from mice at 3 months, before (Tyr) and after exposure to 1 nM anemonia toxin-II (ATX-II) (*n* = 5 cells from 4 hearts); data are shown as mean ± s.e.m. and scatter plots. **P*<0.05 versus Tyr (paired *t*-test and Wilcoxon signed rank test); *NP*: non-parametric analysis. (**c**) Current traces in voltage-clamp mode showing the effects of 1 nM ATX-II on I_{NaL} in a young myocytes. I_{NaL} was elicited by a depolarizing step from V_h -70 mV to -40 mV. Scale bars: 100 ms, 400 pA.

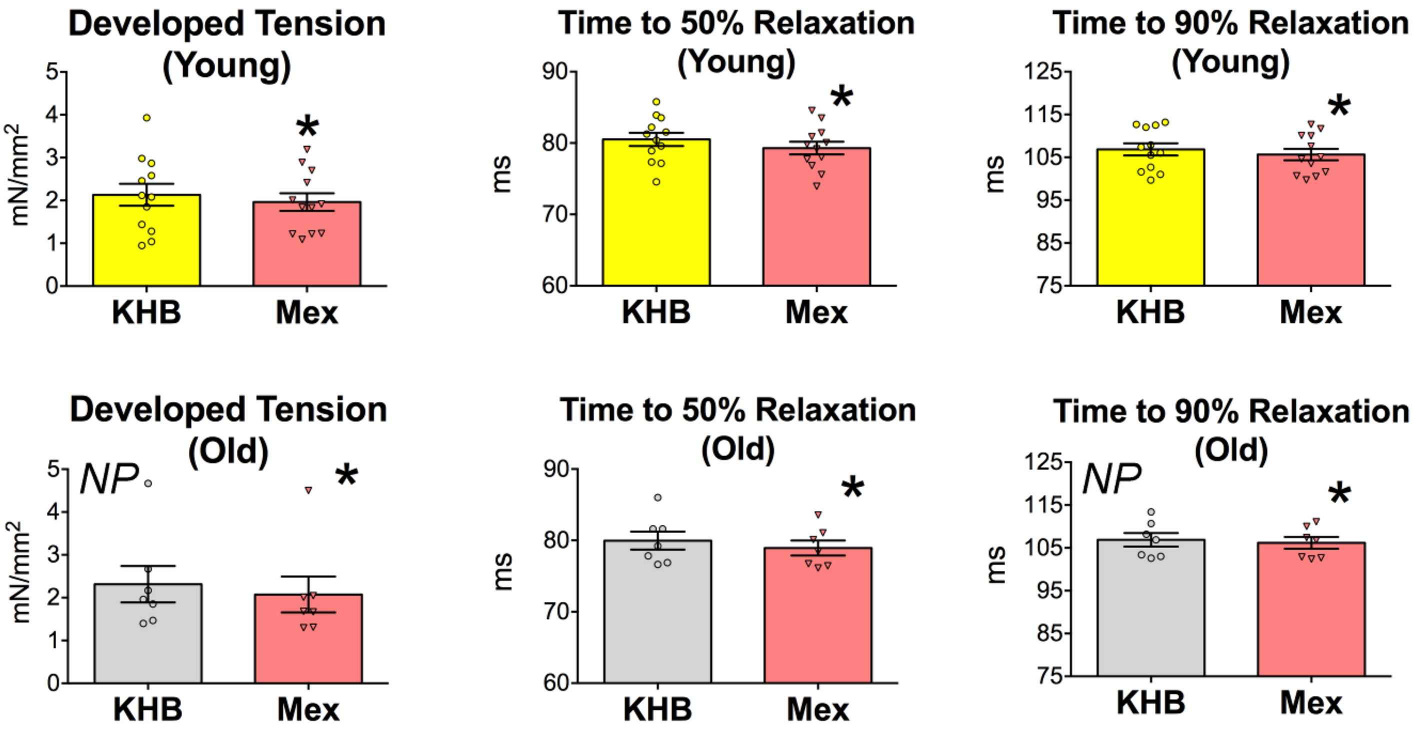

Supplementary Figure 25. I_{NaL} modulates myocardial contractility. Data obtained from muscles of male mice at 3-6 months (Young, n = 12) and 30-33 months (Old, n = 7) before and after exposure to 10 µM mexiletine are shown as mean ± s.e.m. and scatter plots. *P<0.05 versus KHB (paired t-test and Wilcoxon signed rank test); *NP*: non-parametric analysis.

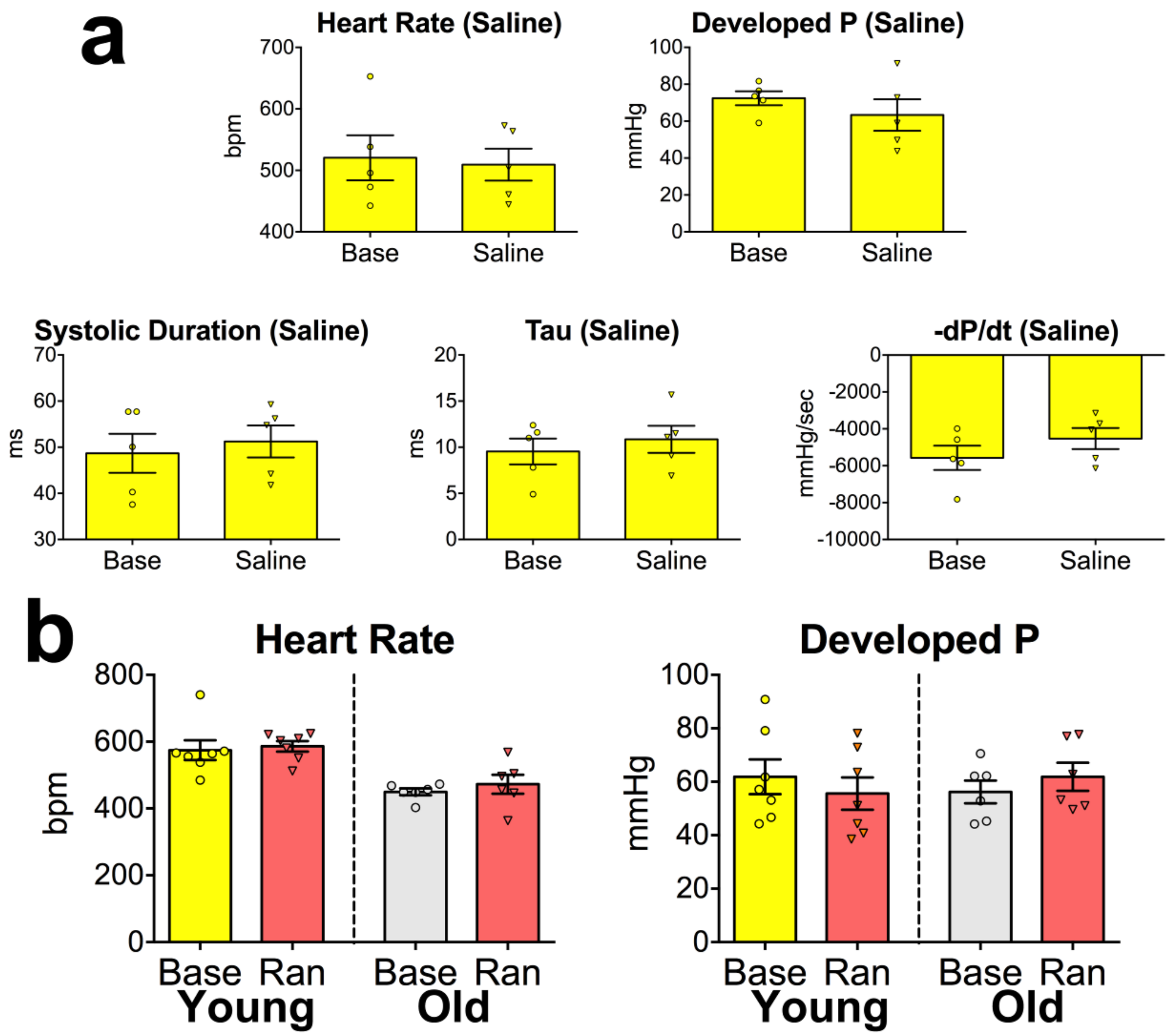

Supplementary Figure 26. Inhibition of I_{NaL} and cardiac function. (**a**) Quantitative data for LV hemodynamic parameters obtained in female mice at 7-11 months at baseline (Base) and ~10 min following bolus infusion of saline (*n* = 5). Data are shown as mean ± s.e.m and scatter plots. This intervention did not alter ventricular function. (**b**) Additional LV hemodynamic parameters for experiments reported in Fig. 9a. Quantitative data was obtained in male mice at 5-6 months (Young) and at 29-33 months (Old) at baseline (Base) and ~10 min following bolus infusion of ranolazine (2.5-5 mg kg^{-1} body weight, i.v.) (Young, *n* = 7; Old *n* = 6). Data are shown as mean ± s.e.m and scatter plots. *P<0.05 versus Base (paired *t*-test).

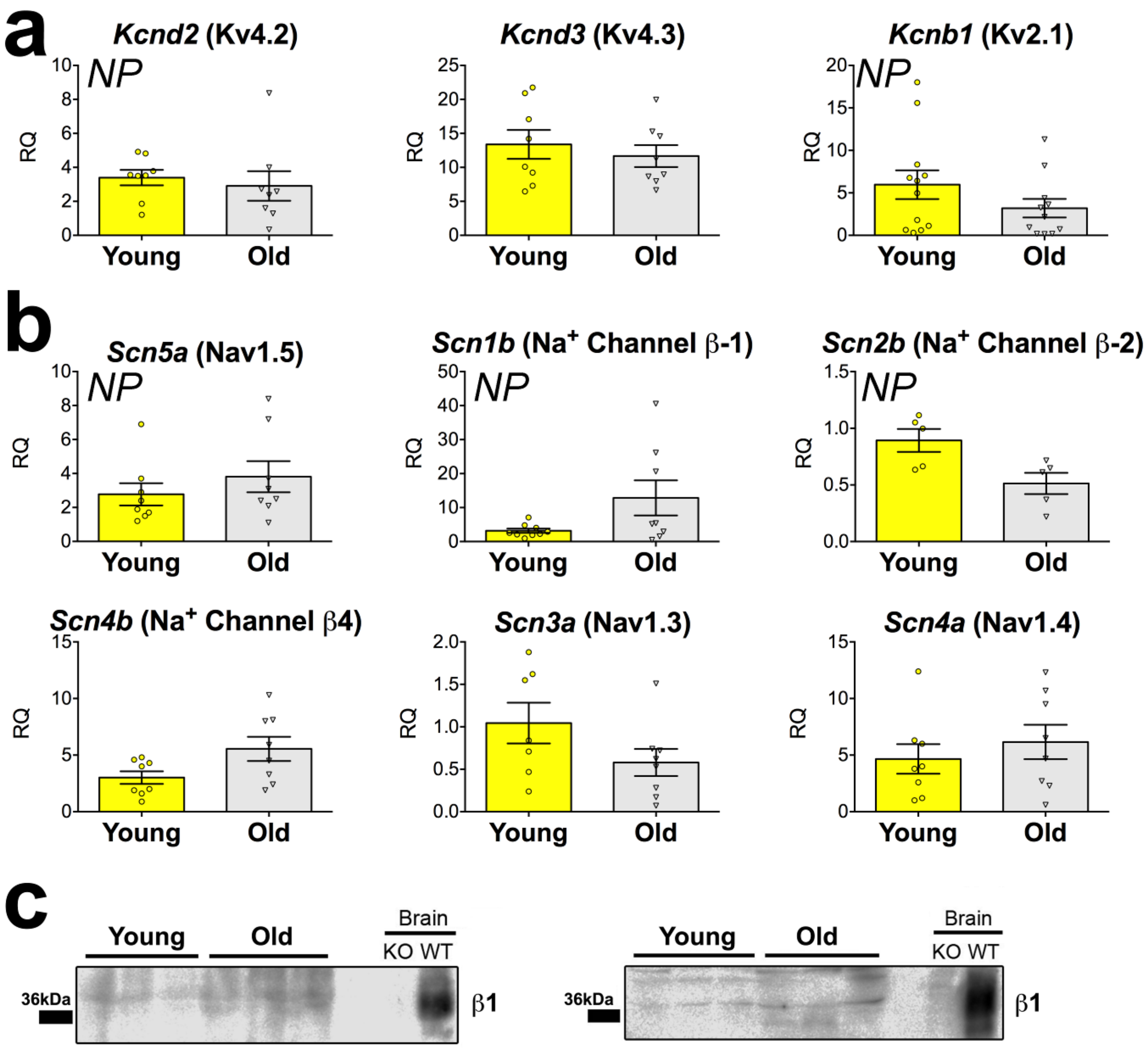

Supplementary Figure 27. Aging and K^+ and Na^+ channel subunits. (**a**) Quantitative data for expression of genes related to channels mediating I_{to} (*Kcnd2* and *Kcnd3*) and I_{ss} (*Kcnb1*) in myocytes from male mice at 3-4 months (Young, *n* = 8-12) old and 27-33 months (Old, *n* = 8-11) are shown as mean ± s.e.m and scatter plots. RQ: relative quantity with respect to β-2-microglobulin; *NP*: non-parametric analysis. (**b**) Quantitative data for expression of genes related to Na^+ channels in myocytes from male mice at 3-4 months (Young, *n* = 5-8) old and 27-33 months (Old, *n* = 5-8) are shown as mean ± s.e.m and scatter plots. Transcripts for Nav1.1, and Nav1.6 were poorly detectable (data not shown). RQ: relative quantity with respect to β-2-microglobulin; *NP*: non-parametric analysis. (**c**) Expression of Na^+ channel β1 subunit protein by Western blotting in LV myocyte (left) and LV myocardium (right) of mice at 3-4 months (Young) and old 30-31 months (Old). β1 levels in the brains of *Scn1b* null (KO) and wild type (WT) mice were used as negative and positive controls, respectively. A total of 150 µg of LV myocytes or LV myocardial protein lysate per lane and 25 µg of brain membrane protein per lane were loaded.

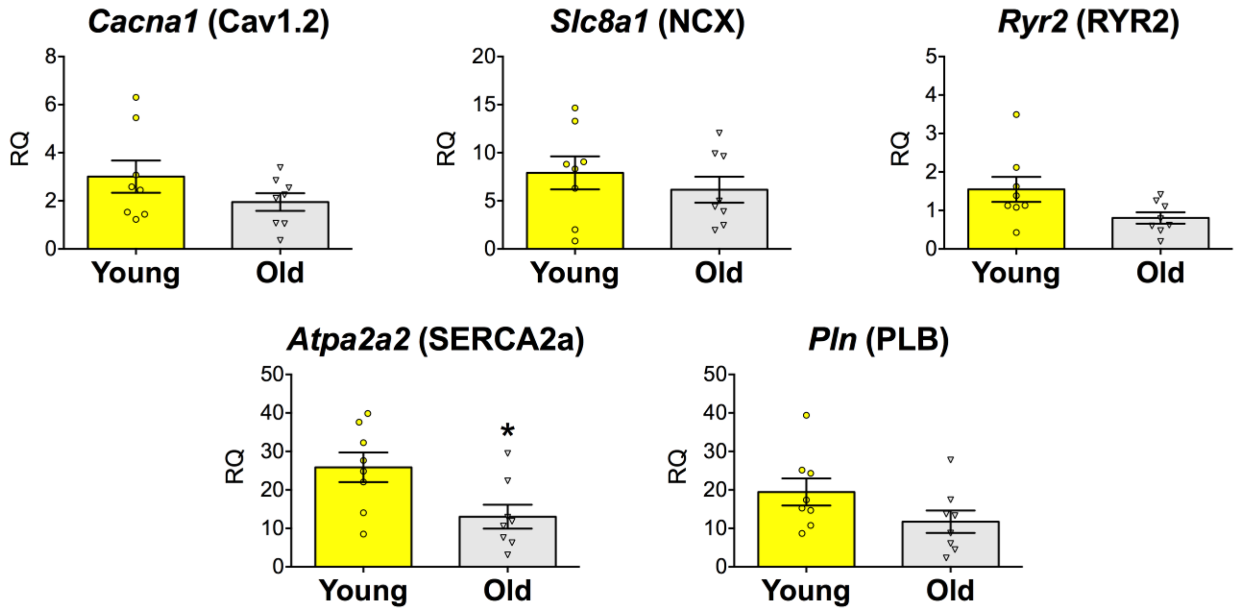

Supplementary Figure 28. Aging and gene expression profile for Ca^{2+} handling molecules. Quantitative data for expression of genes related to L-type Ca^{2+} current (*Cacna1*), NCX (*Slc8a1*), Ryrs, (*Ryr2*), SERCA2a (*Atpa2a2*), and phospolamban (*Pln*) in myocytes from male mice at 3 months (Young, n = 6) old and 27-28 months (Old, n = 6) are shown as mean ± s.e.m and scatter plots. RQ: relative quantity with respect to β-2-microglobulin; *P<0.05 versus Young (Student's *t*-test).

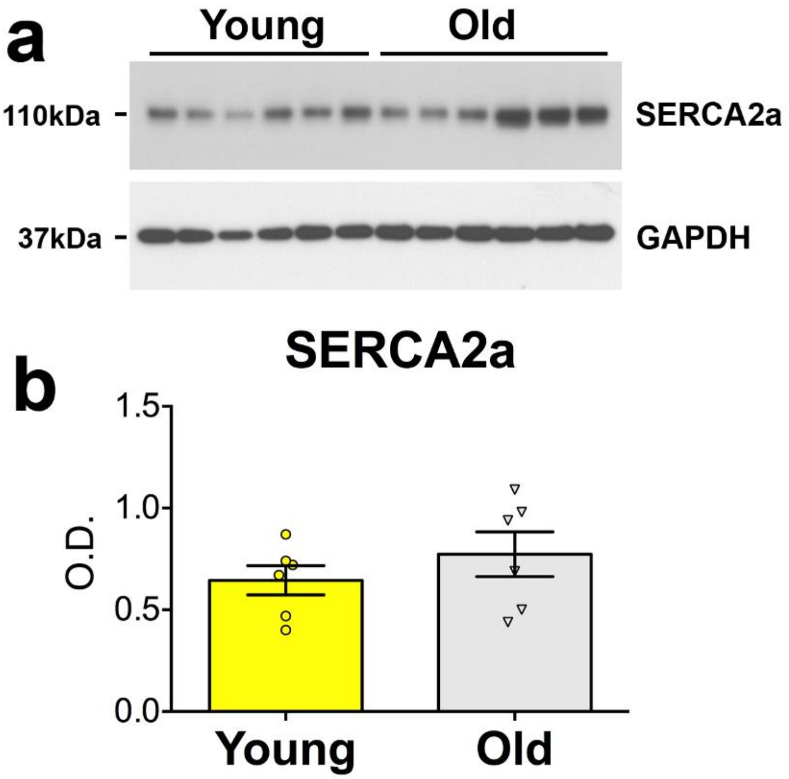

Supplementary Figure 29. Aging and SERCA expression. (**a**) Expression of SERCA proteins in LV myocytes from male mice at 3 months (Young, *n* = 6) and old 27-28 months (Old, *n* = 6), by Western blotting. GAPDH is the loading condition. (**b**) Quantitative data for SERCA expression in myocytes shown in **a** are reported as mean ± s.e.m and scatter plots.

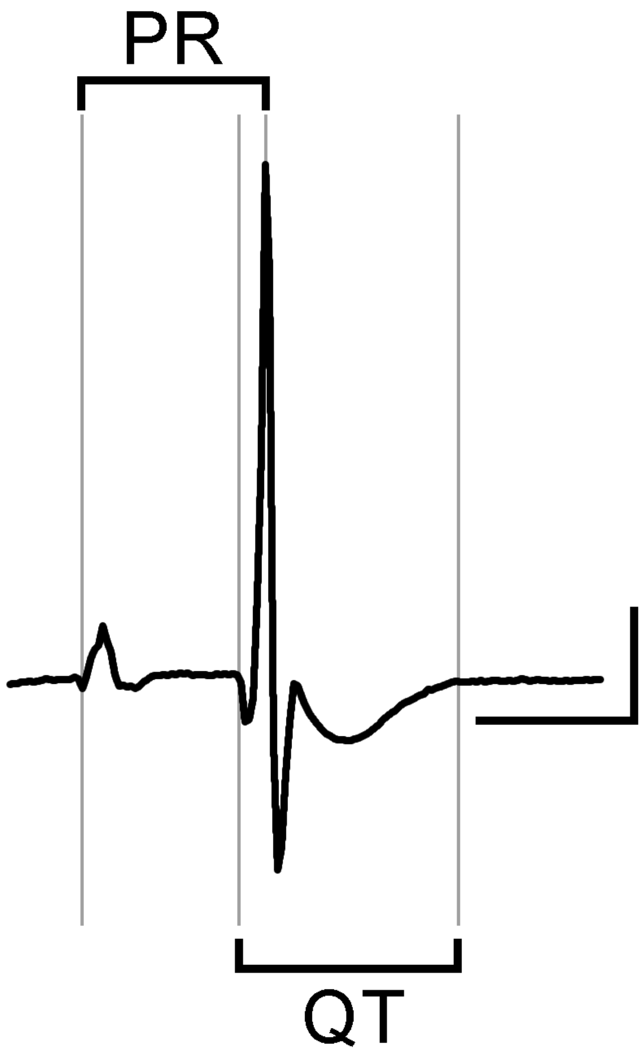

Supplementary Figure 30. ECG parameters. PR and QT intervals are defined on the electrocardiogram obtained from a young mouse. Scale bars: 40 ms, 0.2 mV.

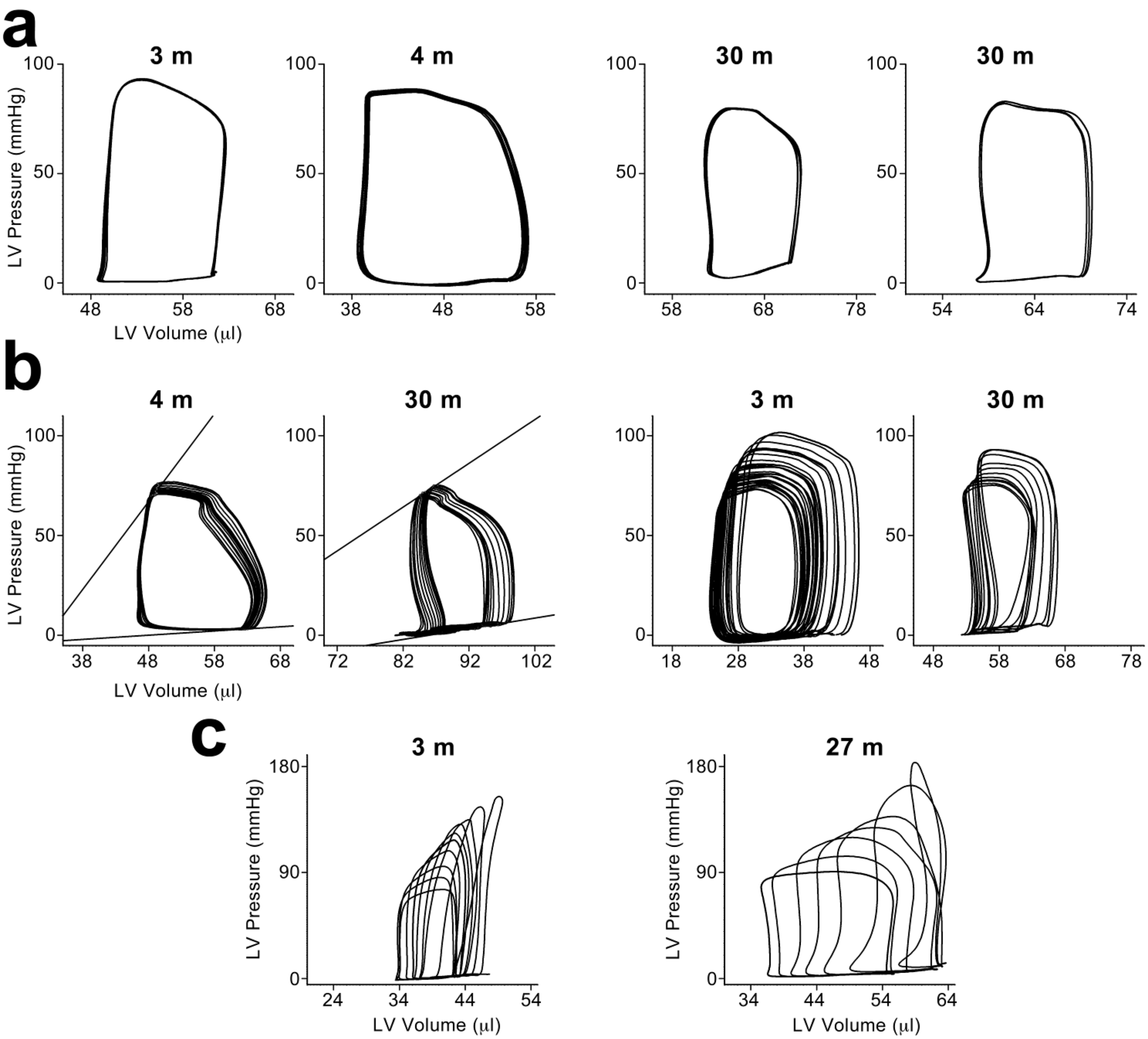

Supplementary Figure 31. Pressure-volume (PV) loops. (**a**) PV loops at baseline obtained from young and old mice (m, months of age). (**b**) PV loops following occlusion of the inferior vena cava in young and old mice (m, months of age). In the left two panels, slopes of the LV end-diastolic PV relation and end-systolic PV relation are reported. (**b**) PV loops following occlusion of the aortic arch in young and old mice (m, months of age).

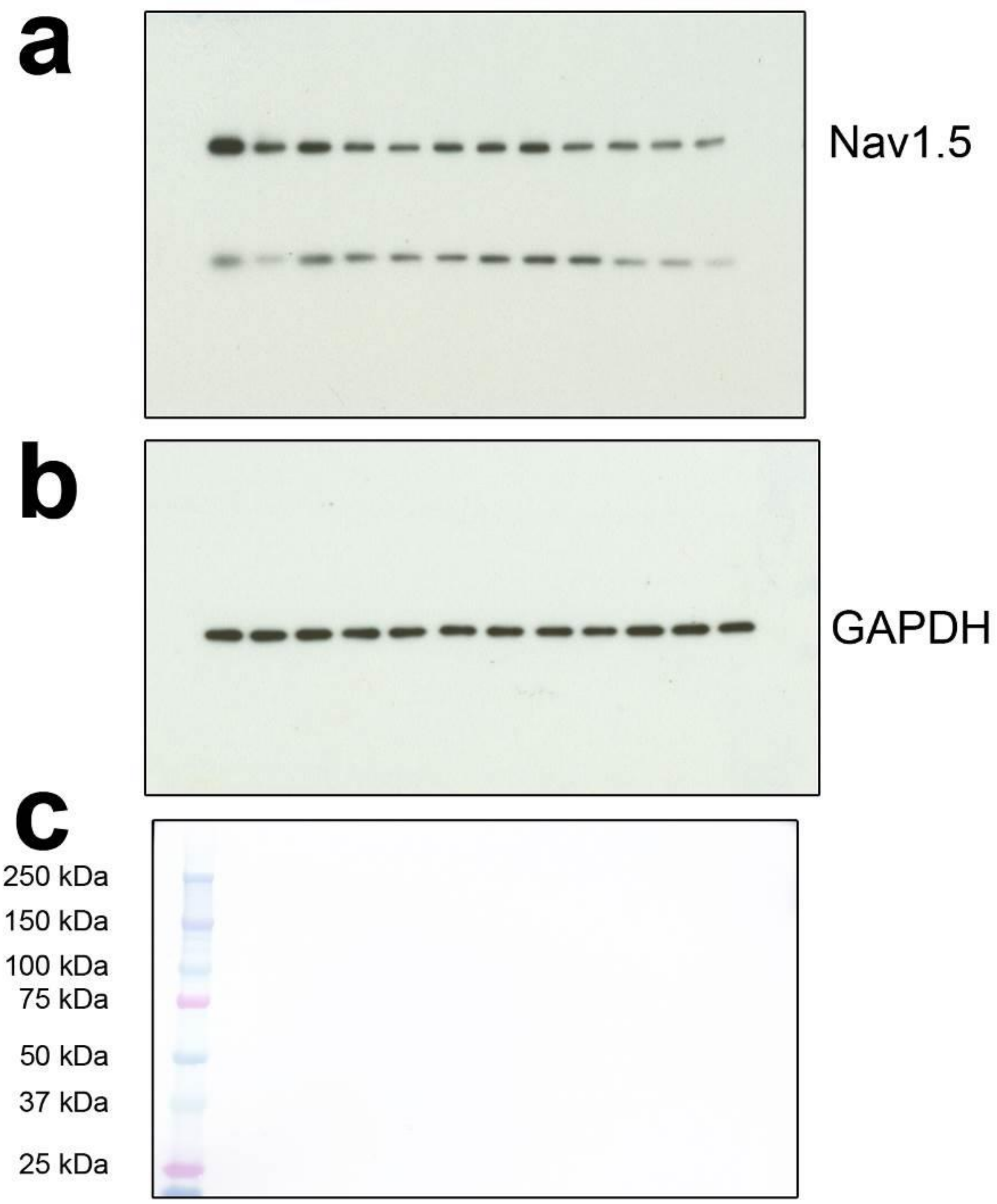

Supplementary Figure 32. Uncropped scans for data shown in Fig. 10b. (**a**) Scan of film with Western blot bands relative to proteins bound to Nav1.5 antibody. (**b**) Scanned image of film with Western blot bands relative to proteins bound to GAPDH antibody. (**c**) Scan of polyvinylidene difluoride (PVDF) membrane with molecular weight markers employed in Nav1.5 and GAPDH analysis.

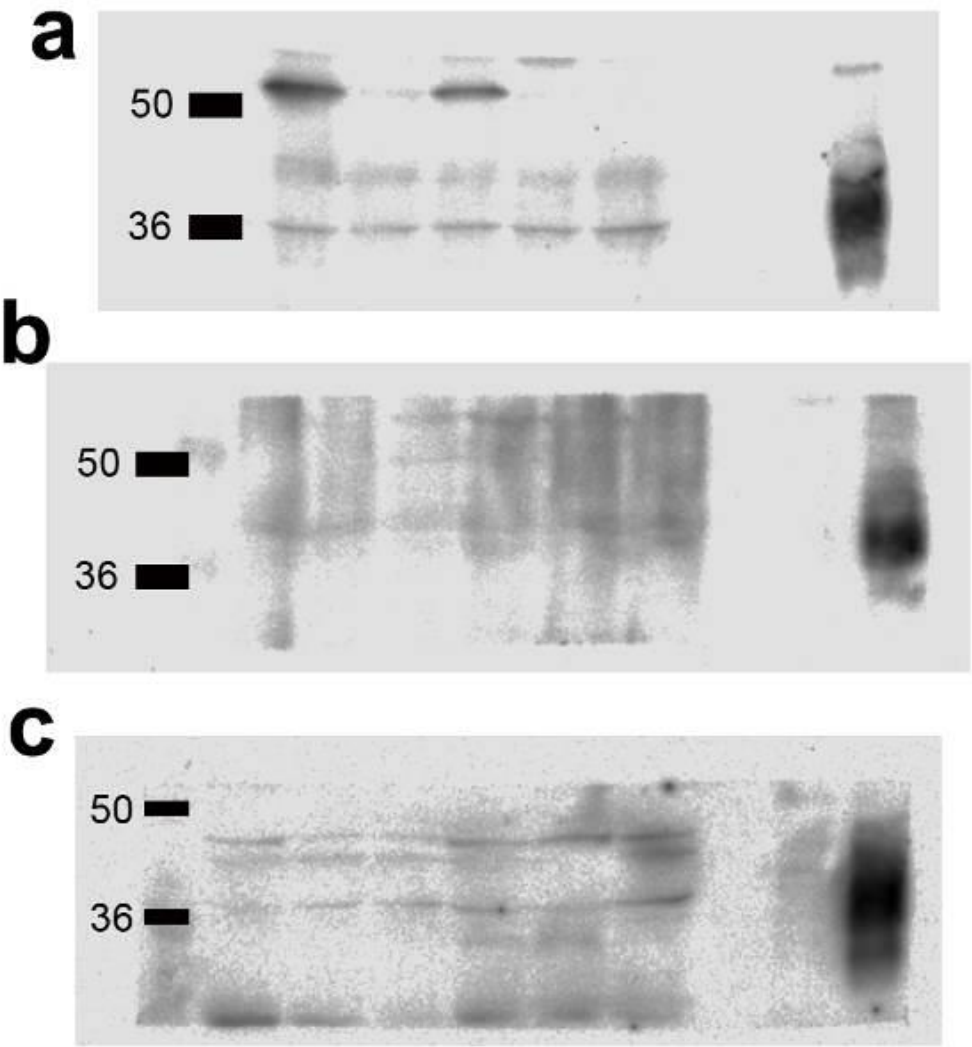

Supplementary Figure 33. Uncropped digital images obtained using LI-COR system shown in Fig. 10c and Supplementary Fig. 27c. (**a**) Digital image relative to Western blot bands of proteins bound to Na^+ channel β1-subunit antibody shown in Fig. 10c. (**b**) Digital image relative to Western blot bands of proteins bound to Na^+ channel β1-subunit antibody shown in Supplementary Fig. 27c, left panel. (**c**) Digital image relative to Western blot bands of proteins bound to Na^+ channel β1-subunit antibody shown in Supplementary Fig. 27c, right panel.

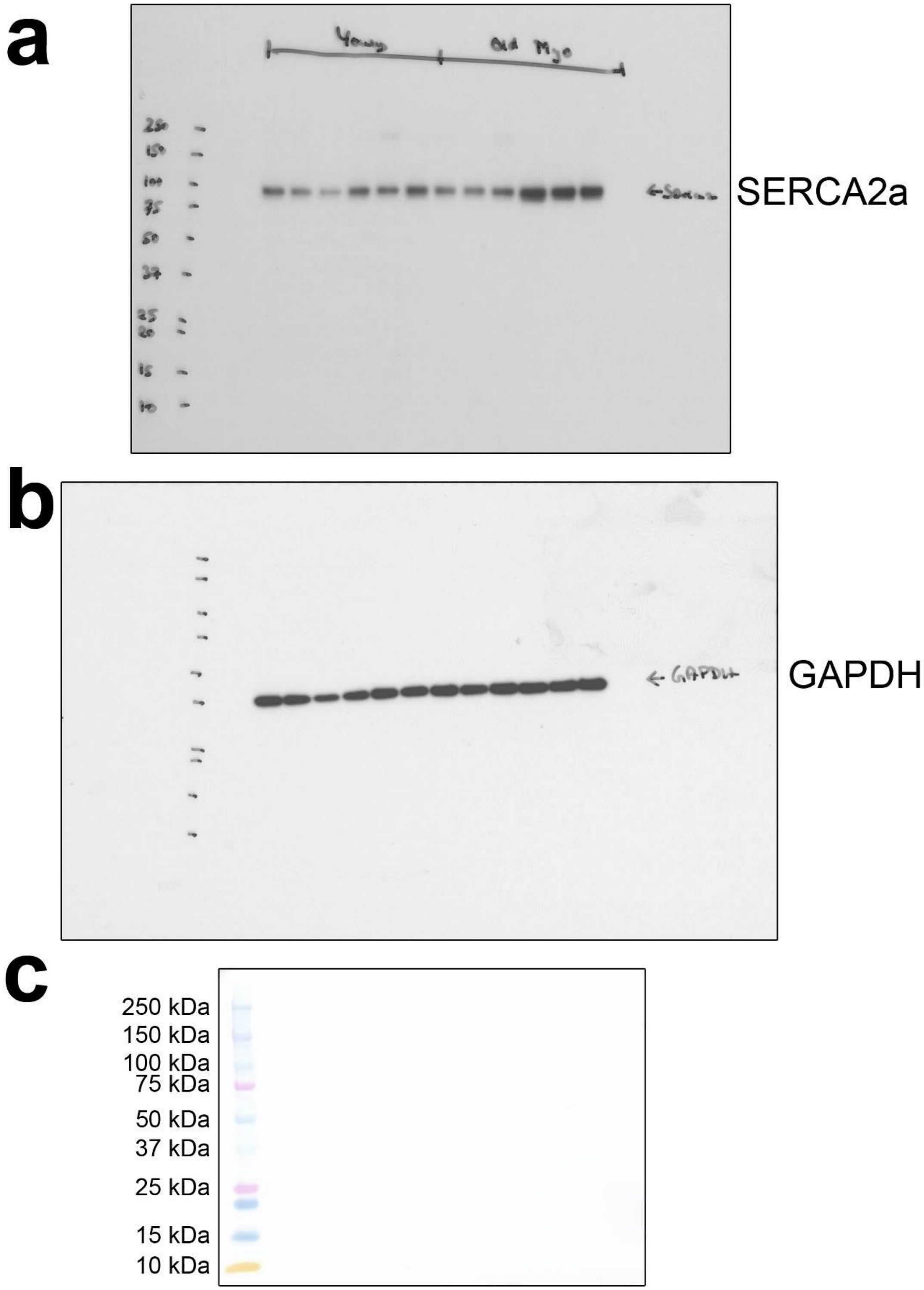

Supplementary Figure 34. Uncropped scans for data shown in Supplementary Fig. 39a. (**a**) Scan of film with Western blot bands relative to proteins bound to SERCA2a antibody. (**b**) Scanned image of film with Western blot bands relative to proteins bound to GAPDH antibody. (**c**) Scan of polyvinylidene difluoride (PVDF) membrane with molecular weight markers employed in SERCA2a and GAPDH analysis.

Supplementary Table 1. Primers for PCR Analysis.

Gene	Primers
Mouse Tgfb1	F: 5'-GAAGGACCTGGGTTGGAAGT-3'
	R: 5'-TGGTTGTAGAGGGCAAGGAC-3'
Mouse Vim	F: 5'-AGAGAGAGGAAGCCGAAAGC-3'
	R: 5'-TCCACTTTCCGTTCAAGGTC-3'
Mouse Hprt	F: 5'-AGCCCCAAAATGGTTAAGGT-3'
	R: 5'-CAAGGGCATATCCAACAACA-3'
Mouse Scn1a	F: 5'-AGTAACCCTCCCGACTGGACAAAG-3'
	R: 5'-CCATGGGTCGCGAAGGAAAG-3'
Mouse Scn3a	F: 5'-GAGAGCGACGCAACAGTAACG-3'
	R: 5'-CCAGCATGGTGGACACTTCT-3'
Mouse Scn4a	F: 5'-CCTGGAACTGGCTGGACTTCAGTG-3'
	R: 5'-GATCAGGGCTCCCACGATCG-3'
Mouse Scn5a	F: 5'-AGTACCCAGAAGACCTTCATCGTG-3'
	R: 5'-GGTGCACATGATGAGCATGC-3'
Mouse Scn8a	F: 5'-GACTTTGACCCGTACTATTTG-3'
	R: 5'-ATCATGCTGAAGACTGAATG-3'
Mouse Scn1b	F: 5'-GCGTCGTCAAGAAGATCCAC-3'
	R: 5'-CATCTCTGCCACAAGCCATA-3'
Mouse Scn2b	F: 5'-CAACTCCTGCTACACCGTGA-3'
	R: 5'-TCATTCGGAACTGGAGGAAC-3'
Mouse Scn4b	F: 5'-GGCAATACTCAGGCGAGATG-3'
	R: 5'-ATAGCGTAGATGGTGGTGGC-3'
Mouse Kcna4	F: 5'-AAAGGGGAAACAAATCACCG-3'
	R: 5'-GCAGGAAATGAAGAGCATCC-3'
Mouse Kcna5	F: 5'-ATGAGGCCCATCACTGTAGG-3'
	R: 5'-AAAATTGGAGACGATGACGG-3'

Mouse Kcnb1	F: 5'-CTGGAGAAGCCCAACTCATC-3'
	R: 5'-TGTTGAGTGACAGGGCAATG-3'
Mouse Kcnd2	F: 5'-TCAGCAAGCAAGTTCACCAG-3'
	R: 5'-TTCCCTGCTATGGTTTTTGG-3'
Mouse Kcnd3	F: 5'-TCTGCCAGCAAGTTCACAAG-3'
	R: 5'-TTCCCTGCGATTGTCTTAGG-3'
Mouse Kcnip2	F: 5'-GGAGAGTTTGTCCGAATCCC-3'
	R: 5'-AGCTTGAGGAAACGCTGCT-3'
Mouse Cacna1c	F: 5'-CACCAACTCCAACCTGGAAC-3'
	R: 5'-TGGAAGAGAAGTCCGTAGGC-3'
Mouse Ryr2	F: 5'-ATCTTGGTCAGCGTGTCCTC-3'
	R: 5'-TGCTTCACTTCCTGAGCTGAT-3'
Mouse Atpa2a	F: 5'-TGCAACTCGGTCATAAAGCA-3'
	R: 5'-TGGCTTGTTTGGGGTACAAT-3'
Mouse Slc8a1	F: 5'-AGGAGACAGACCAGCTTCCA-3'
	R: 5'-CAAAACCAGAGCCCCATCTA-3'
Mouse Pln	F: 5'-CCCAGCTAAGCTCCCATAAG-3'
	R: 5'-AACAGGCAGCCAAATGTG-3'
Mouse β2m	F: 5'-CTCGGTGACCCTGGTCTTTCTG-3'
	R: 5'-ATGTGAGGCGGGTGGAACTG-3'

Supplementary Table 2. Antibodies for Western Blot Analysis.

Antigen	Antibody	Dilution	Manufacturer
Anti-SCN5A (Nav1.5)	rabbit polyclonal	1:500	Gift from Dr. Hugues Abriel
Anti-SCN1B (Navβ1)	rabbit monoclonal	1:750	Cell Signaling Technology
Anti-SERCA2a	mouse monoclonal	1:500	Sigma-Aldrich
GAPDH	rabbit monoclonal	1:2000	Cell Signaling Technology

Supplementary Table 3. Fitting Parameters.

**I_{NaL}, Steady State Inactivation**		**$V_{1/2G}$**	**k_G**	**R^2**	**P**
Young		-61.5±0.7 mV	-11.6±0.7 mV	0.9974	0.9954
Old		-61.5±0.5 mV	-11.1±0.5 mV	0.9985	
**I_{NaL}, Recovery from Inactivation**			**τ**	**R^2**	**P**
Young			655±17 ms	0.9965	0.6254
Old			756±80 ms	0.9968	
**I_{Na}, Conductance**	**g_{max}**	**$V_{1/2G}$**	**k_G**	**R^2**	**P**
Young	0.468±0.004 mS·μF^{-1}	-52.9±0.2 mV	3.1±0.2 mV	0.9992	0.6270
Old	0.427±0.004 mS·μF^{-1}	-54.0±0.2 mV	3.2±0.2 mV	0.9992	
**I_{Na}, Activation**		**$V_{1/2G}$**	**k_G**	**R^2**	**P**
Young		-52.7±0.4 mV	3.9±0.3 mV	0.9970	0.8170
Old		-53.7±0.3 mV	3.5±0.3 mV	0.9979	
**I_{Na}, Steady State Inactivation**		**$V_{1/2G}$**	**k_G**	**R^2**	**P**
Young		-81.0±0.1 mV	-5.7±0.1 mV	0.9999	0.9979

Old		-81.0±0.1 mV	-5.5±0.1 mV	0.9999	
**I_{Na}, Recovery from Inactivation**			**τ**	**R^2**	**P**
Young			17.7±0.6 ms	0.9988	0.9169
Old			18.1±0.8 ms	0.9981	
**I_{CaL}, Conductance**	**g_{max}**	**$V_{1/2G}$**	**k_G**	**R^2**	**P**
Young	0.0589±0.002 $mS{\cdot}\mu F^{-1}$	-8.6±0.8 mV	3.95±0.8 mV	0.8408	0.0864
Old	0.0662±0.002 $mS{\cdot}\mu F^{-1}$	-7.2±0.8 mV	4.29±0.6 mV	0.8609	
**I_{CaL}, Activation**		**$V_{1/2G}$**	**k_G**	**R^2**	**P**
Young		-7.3±0.4 mV	5.0±0.3 mV	0.9552	0.4404
Old		-6.0±0.3 mV	5.1±0.3 mV	0.9650	
**I_{CaL}, Steady State Inactivation**		**$V_{1/2G}$**	**k_G**	**R^2**	**P**
Young		-16.3±0.3 mV	-7.2±0.3 mV	0.9722	0.7419
Old		-16.9±0.3 mV	-7.0±0.3 mV	0.9781	
**AP_{clamp}-Ca^{2+} Transient Decay**	**F_p/F_0 (peak fluorescence)**	**τ**	**R^2**		
Young – APD_{50} 5ms	2.163±0.1043 F/F_0	177.2 ms	0.3868		
Young – APD_{50} 7ms	2.204±0.1072 F/F_0	170.4 ms	0.3907		
Young – APD_{50} 17ms	2.504±0.1250 F/F_0	178.1 ms	0.4250		
Young – APD_{50} 26ms	2.628±0.1346 F/F_0	169.7 ms	0.4310		
Young – APD_{50} 80ms	3.591±0.1841 F/F_0	176.9 ms	0.5136		
Old – APD_{50} 5ms	1.842±0.05437 F/F_0	171.7 ms	0.6195		
Old – APD_{50} 7ms	1.967±0.06877 F/F_0	169.2 ms	0.5766		

Old – APD_{50} 17ms	2.181±0.07849 F/F_0	166.5 ms	0.6123	
Old – APD_{50} 26ms	2.323±0.08127 F/F_0	165.0 ms	0.6481	
Old – APD_{50} 80ms	2.960±0.1418 F/F_0	168.1 ms	0.5754	

**AP_{clamp}-Ca^{2+} Transient Decay**	**Slope**	**R^2**	**P**
Young - Ca^{2+} Transient Amplitude	0.01683±0.001 $F/F_0 \cdot ms^{-1}$	0.9925	0.044
Old - Ca^{2+} Transient Amplitude	0.01265±0.0014 $F/F_0 \cdot ms^{-1}$	0.9642	
Young - Ca^{2+} Transient Time to Peak	0.2370±0.03442 $ms \cdot ms^{-1}$	0.9405	0.5484
Old – Ca^{2+} Transient Time to Peak	0.2141±0.01071 $ms \cdot ms^{-1}$	0.9925	
Young - 30% Ca^{2+} Transient Decay	0.2270±0.031 $ms \cdot ms^{-1}$	0.9485	0.2712
Old - 30% Ca^{2+} Transient Decay	0.1878±0.011 $ms \cdot ms^{-1}$	0.9903	
Young - 90% Ca^{2+} Transient Decay	-0.5773±0.142 $ms \cdot ms^{-1}$	0.8459	0.3883
Old - 90% Ca^{2+} Transient Decay	-0.7359±0.094 $ms \cdot ms^{-1}$	0.9533	

**Diastolic Length-Tension Relation**		**a**	**b**	**R^2**	**P**
Young		20. 6±1.5 $mN \cdot mm^{-2}$	10.5±1.6 $mN \cdot mm^{-2}$	0.9090	<0.0001 ANOVA
Old		24.5±2.6 $mN \cdot mm^{-2}$	29.3±2.9 $mN \cdot mm^{-2}$	0.9646	
Young Mex		17.6±3.6 $mN \cdot mm^{-2}$	5.5±4.1 $mN \cdot mm^{-2}$	0.7829	

Old Mex		29.5±2.8 $mN \cdot mm^{-2}$	9.7±3.2 $mN \cdot mm^{-2}$	0.9152	
Young ATX-II		19.7±2.7 $mN \cdot mm^{-2}$	28.4±3.1 $mN \cdot mm^{-2}$	0.9435	<0.0001 vs. Young
